# PERK signaling promotes mitochondrial elongation by remodeling membrane phosphatidic acid

**DOI:** 10.15252/embj.2023113908

**Published:** 2023-06-12

**Authors:** Valerie Perea, Christian Cole, Justine Lebeau, Vivian Dolina, Kelsey R Baron, Aparajita Madhavan, Jeffery W Kelly, Danielle A Grotjahn, R Luke Wiseman

**Affiliations:** ^1^ Department of Molecular Medicine Scripps Research La Jolla CA USA; ^2^ Department of Chemistry Scripps Research La Jolla CA USA; ^3^ Skaggs Institute for Chemical Biology Scripps Research La Jolla CA USA; ^4^ Department of Integrative, Structural, and Computational Biology Scripps Research La Jolla CA USA

**Keywords:** endoplasmic reticulum (ER) stress, mitochondrial morphology, phosphatidic acid, unfolded protein response (UPR), Organelles, Translation & Protein Quality

## Abstract

Endoplasmic reticulum (ER) stress and mitochondrial dysfunction are linked in the onset and pathogenesis of numerous diseases. This has led to considerable interest in defining the mechanisms responsible for regulating mitochondria during ER stress. The PERK signaling arm of the unfolded protein response (UPR) has emerged as a prominent ER stress‐responsive signaling pathway that regulates diverse aspects of mitochondrial biology. Here, we show that PERK activity promotes adaptive remodeling of mitochondrial membrane phosphatidic acid (PA) to induce protective mitochondrial elongation during acute ER stress. We find that PERK activity is required for ER stress‐dependent increases in both cellular PA and YME1L‐dependent degradation of the intramitochondrial PA transporter PRELID1. These two processes lead to the accumulation of PA on the outer mitochondrial membrane where it can induce mitochondrial elongation by inhibiting mitochondrial fission. Our results establish a new role for PERK in the adaptive remodeling of mitochondrial phospholipids and demonstrate that PERK‐dependent PA regulation adapts organellar shape in response to ER stress.

## Graphical abstract

PERK regulates the composition of mitochondrial phospholipids, thus adapting their morphology to ER stress.

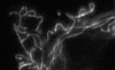

## Introduction

Endoplasmic reticulum (ER) and mitochondrial function are coordinated through the interorganellar transport of metabolites such as lipids and Ca^2+^ (Rowland & Voeltz, 2012; Csordas *et al*, 2018; Wu *et al*, 2018). As a consequence of this coordination, ER stress can be transmitted to mitochondria and promote mitochondrial dysfunction implicated in the pathophysiology of numerous diseases including diabetes, cardiovascular disorders, and many neurodegenerative diseases (Area‐Gomez *et al*, 2012; Brown & Naidoo, 2012; De Strooper & Scorrano, 2012; Schon & Area‐Gomez, 2013; Stutzbach *et al*, 2013; Liu & Dudley Jr., 2015; Smith & Mallucci, 2016; Rodriguez‐Arribas *et al*, 2017; Xiang *et al*, 2017; Morris *et al*, 2018; Hughes & Mallucci, 2019; Rocha *et al*, 2020; Ren *et al*, 2021). This pathologic relationship between ER stress and mitochondria has led to significant interest in identifying the stress‐responsive signaling pathways responsible for regulating mitochondria in response to ER insults.

**Figure 1:**
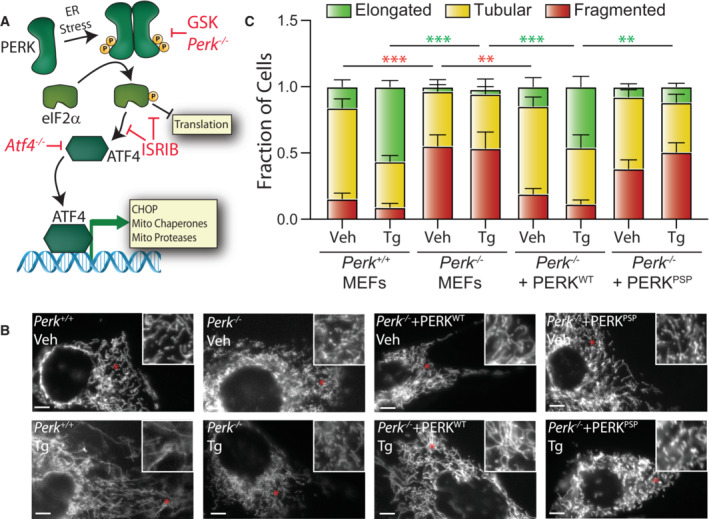
ER stress‐induced mitochondrial elongation is impaired in cells expressing a hypomorphic PERK variant Illustration showing the mechanism of PERK‐regulated transcriptional and translational signaling. Specific genetic and pharmacologic manipulations used to disrupt PERK signaling are shown. Adapted from Lebeau *et al* (2018).Representative images of *Perk* ^+/+^ MEFs, *Perk* ^−/−^ MEFs, or *Perk* ^−/−^ MEFs transfected with wild‐type PERK^WT^ or the PSP‐associated PERK allele (PERK^PSP^) treated for 6 h with thapsigargin (Tg; 500 nM). The inset shows twofold magnification of the image centered on the asterisk. Scale bars, 5 μm.Quantification of fragmented (red), tubular (yellow), or elongated (green) mitochondria from the images shown in (B). Error bars show SEM for *n* = 5 independent experiments. **P < 0.01, ***P < 0.005 for two‐way ANOVA (red indicates comparison between fragmented mitochondria fractions; green indicates comparisons between elongated mitochondria fractions).

The PERK arm of the unfolded protein response (UPR) has emerged as a prominent stress‐responsive signaling pathway involved in regulating mitochondria during ER stress (Rainbolt *et al*, 2014; Quintana‐Cabrera & Soriano, 2019; Cannon & Nedergaard, 2021; Almeida *et al*, 2022). PERK is an ER transmembrane protein that is activated in response to ER stress through a mechanism involving oligomerization and autophosphorylation of its cytosolic kinase domain (Fig 1A; Walter & Ron, 2011; Gardner *et al*, 2013; Hetz & Papa, 2018). Activated PERK selectively phosphorylates serine 51 of the α subunit of eukaryotic initiation factor 2 (eIF2α). Phosphorylated eIF2α prevents formation of ribosomal initiation leading to global mRNA translational attenuation, which functions to reduce the load of newly synthesized proteins during ER stress (Walter & Ron, 2011; Gardner *et al*, 2013; Hetz & Papa, 2018). PERK‐dependent eIF2α phosphorylation also leads to the selective translation and activation of transcription factors, such as ATF4, through upstream open reading frames (uORFs) in the 5' untranslated region of these mRNAs (Wek & Cavener, 2007; Walter & Ron, 2011; Gardner *et al*, 2013; Hetz & Papa, 2018). ATF4 regulates the expression of several stress‐responsive genes including redox factors, amino acid biosynthesis genes, the eIF2α phosphatase *PPP1R15A*/*GADD34*, and the pro‐apoptotic transcription factor *DDIT3*/*CHOP* (Harding *et al*, 2000; Wek & Cavener, 2007; Han *et al*, 2013). Through this combination of translational attenuation and transcriptional signaling, PERK promotes both adaptive and pro‐apoptotic signaling in response to varying levels and extents of ER stress (Haucke, 1999; Harding *et al*, 2000; Wek & Cavener, 2007; Lin *et al*, 2009; Walter & Ron, 2011; Gardner *et al*, 2013; Han *et al*, 2013; Sovolyova *et al*, 2014; Iurlaro & Munoz‐Pinedo, 2016; Halliday *et al*, 2017a; Hetz & Papa, 2018; Hetz *et al*, 2020).

PERK localizes to ER‐mitochondrial contact sites, positioning this protein to coordinate regulation of these two organelles in response to cellular insults (Verfaillie *et al*, 2012). Consistent with this, PERK signaling regulates diverse aspects of mitochondrial proteostasis and function (Almeida *et al*, 2022). PERK regulates mitochondrial protein import, biogenesis, and cristae remodeling in brown adipocytes in response to cold exposure or beta‐adrenergic stimulation (Kato *et al*, 2020; Latorre‐Muro *et al*, 2021). Furthermore, the PERK‐regulated transcription factor ATF4 increases mitochondrial respiratory chain activity during ER stress or nutrient deprivation through a mechanism involving SCAF1‐dependent increases in supercomplex formation (Balsa *et al*, 2019). ATF4 also regulates the expression of numerous mitochondrial proteostasis factors including the mitochondrial HSP70 *HSPA9* and the AAA+ quality control protease *LONP1* to increase mitochondrial proteostasis capacity during ER stress (Hori *et al*, 2002; Han *et al*, 2013). Furthermore, PERK‐dependent translational attenuation regulates mitochondrial protein import by selectively decreasing protein concentrations of the core TIM23 subunit TIM17A, a process dependent on the mitochondrial AAA+ protease YME1L (Rainbolt *et al*, 2013).

PERK signaling also promotes adaptive mitochondrial elongation downstream of eIF2α phosphorylation‐dependent translational attenuation (Lebeau *et al*, 2018). This increase in mitochondrial elongation functions to protect mitochondria during ER stress by preventing premature fragmentation and regulating mitochondrial respiratory chain activity (Lebeau *et al*, 2018). However, the mechanistic basis of PERK‐dependent mitochondrial elongation was previously undefined. Here, we show that PERK induces mitochondrial elongation through the remodeling of mitochondrial membrane phosphatidic acid (PA). Our results suggest a model whereby PERK signaling both increases total mitochondrial PA and inhibits trafficking of PA to the inner mitochondrial membrane. This leads to the accumulation of PA on the outer mitochondrial membrane where it induces mitochondrial elongation by inhibiting mitochondrial fission. These results define a new role for PERK in regulating the amount and localization of mitochondrial membrane phospholipids and show that this remodeling is important for adapting mitochondrial morphology during acute ER stress.

## Results

### A hypomorphic PERK variant inhibits ER stress‐induced mitochondrial elongation

**Figure EV1:**
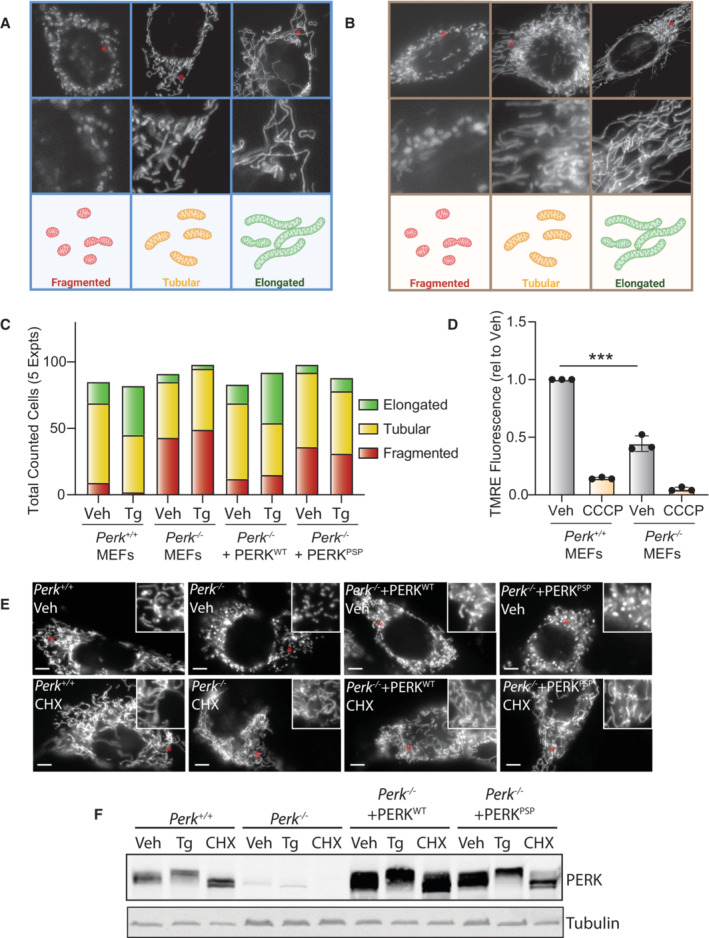
Supplement to Fig 1. ER stress‐induced mitochondrial elongation is impaired in cells expressing a hypomorphic PERK variant Representative images of fragmented, tubular, and elongated mitochondria in MEF^mtGFP^ cells.Representative images of fragmented, tubular, and elongated mitochondria in HeLa cells expressing ^mt^GFP.Total cells counted for each treatment condition for mitochondrial morphology qualifications in Fig 1B and C.Mitochondrial polarization, measured by TMRE fluorescence, in *Perk* ^+/+^ MEFs and *Perk* ^−/−^ MEFs treated 30 min with CCCP (10 μM). Error bars show SEM for *n* = 3 biological replicates. ***P < 0.005 for one‐way ANOVA.Representative images of *Perk* ^+/+^ MEFs, *Perk* ^−/−^ MEFs, or *Perk* ^−/−^ MEFs transfected with wild‐type PERK^WT^ or the PSP‐associated PERK allele (PERK^PSP^) expressing ^mt^GFP treated for 3 h with cycloheximide (CHX; 50 μg/ml). The inset shows twofold magnification of the image centered on the asterisk. Scale bars, 5 μm.Immunoblot of lysates prepared from *Perk* ^+/+^ MEFs, *Perk* ^−/−^ MEFs, or *Perk* ^−/−^ MEFs transfected with wild‐type PERK^WT^ or the PSP‐associated PERK allele (PERK^PSP^) treated for 6 h with thapsigargin (Tg; 500 nM) or cycloheximide (CHX; 50 μg/ml). Source data are available online for this figure.

Pharmacologic inhibition of PERK signaling, but not other arms of the UPR, blocks mitochondrial elongation induced by ER stress (Lebeau *et al*, 2018). Here, we further probed the dependence of ER stress induced mitochondrial elongation on PERK activity in *Perk* ^−/−^ MEFs. We transfected *Perk* ^+/+^ or *Perk* ^−/−^ MEFs with mitochondrial targeted GFP (^mt^GFP) and monitored mitochondrial morphology in cells treated with or without the ER stressor thapsigargin (Tg; a SERCA inhibitor). We then scored cells based on the presence of fragmented, tubular, or elongated mitochondria (see Fig EV1A and B for representative examples). *Perk* ^−/−^ MEFs showed increases in fragmented mitochondria in the absence of treatment (Figs 1B and C, and EV1C). This corresponds with reductions in the mitochondrial membrane potential in *Perk*‐deficient cells, as measured by tetramethylrhodamine ethyl ester (TMRE) staining (Fig EV1D). This suggests that the increase of fragmentation in these cells can be attributed to mitochondrial depolarization. Tg‐induced mitochondrial elongation was also impaired in *Perk*‐deficient cells (Figs 1B and C, and EV1C). However, treatment with cycloheximide (CHX), which induces mitochondrial elongation independent of PERK signaling (Tondera *et al*, 2009; Lebeau *et al*, 2018), reduced the population of fragmented mitochondria in *Perk* ^−/−^ MEFs (Fig EV1E). This indicates that these cells are not deficient in their ability to induce elongation in response to reduced translation. Reconstitution of *Perk* ^−/−^ MEFs with wild‐type PERK restored basal mitochondrial morphology and rescued Tg‐induced mitochondrial elongation (Figs 1B and C, and EV1C). In contrast, reconstitution of *Perk‐*deficient cells with a hypomorphic PERK haplotype implicated in progressive supranuclear palsy (PSP; PERK^PSP^; Hoglinger *et al*, 2011; Yuan *et al*, 2018) did not impact basally fragmented mitochondria or rescue Tg‐induced mitochondrial elongation. However, CHX increased mitochondrial length in *Perk*‐deficient cells expressing PERK^PSP^ (Fig EV1E). We confirmed similar expression of PERK^WT^ and PERK^PSP^ in *Perk* ^−/−^ MEFs by immunoblotting (Fig EV1F). These results implicate PERK signaling in ER stress induced mitochondrial elongation and demonstrate that genetic disruptions in PERK activity impair the regulation of mitochondrial morphology in response to ER stress.

### Overexpression of cytosolic PA lipases inhibits ER stress induced mitochondrial elongation

Mitochondrial morphology is defined by the relative activities of GTPases localized to the inner and outer mitochondrial membranes that regulate organellar fission and fusion. These include the pro‐fission GTPase DRP1 of the outer mitochondrial membrane (OMM) and the pro‐fusion GTPases MFN1 and MFN2 of the OMM and OPA1 of the inner mitochondrial membrane (IMM; Mishra & Chan, 2016; Chan, 2020; Fenton *et al*, 2020; Giacomello *et al*, 2020; Sabouny & Shutt, 2020). Stress‐induced changes in mitochondrial shape can be dictated through posttranslational regulation of these GTPases to alter the relative activities of fusion and fission pathways (Mishra & Chan, 2016; Chan, 2020; Fenton *et al*, 2020; Giacomello *et al*, 2020; Sabouny & Shutt, 2020). Previous results indicate that PERK signaling does not influence the posttranslational regulation of these GTPases (Lebeau *et al*, 2018), suggesting that ER stress‐induced mitochondrial elongation proceeds through an alternative mechanism.

**Figure 2:**
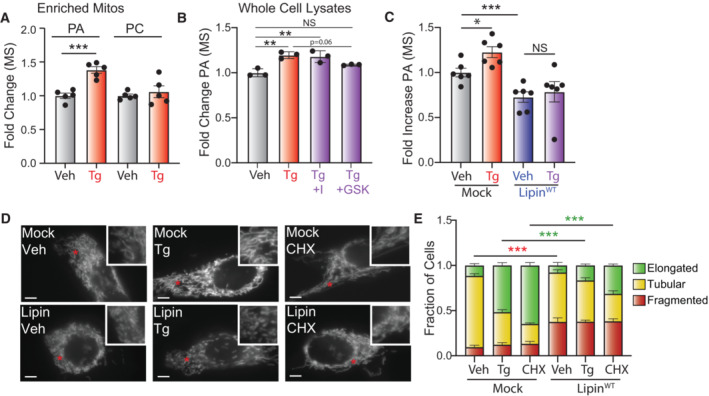
Overexpression of PA lipases inhibits ER stress‐induced mitochondrial elongation Relative amounts of phosphatidic acid (PA) and phosphatidylcholine (PC), measured by untargeted mass spectrometry, in mitochondrial fractions isolated from MEF cells treated for 6 h with vehicle or thapsigargin (Tg; 500 nM). Error bars show SEM for *n* = 5 biological replicates. ***P < 0.005 for one‐way ANOVA.Relative amounts of PA, measured by untargeted mass spectrometry, in whole cell lysates prepared from MEF cells treated for 3 h with vehicle, Tg (500 nM), GSK2656157 (10 μM), or ISRIB (I; 2 μM), as indicated. Error bars show SEM for *n* = 3 biological replicates. **P < 0.01 for one‐way ANOVA.Relative amounts of PA, measured by untargeted mass spectrometry, in whole cell lysates prepared from HeLa cells transfected with mock or wild‐type HA‐Lipin (Lipin^WT^) and treated for 3 h with vehicle or Tg (500 nM). Error bars show SEM for *n* = 6 biological replicates. *P < 0.05, ***P < 0.005 for unpaired *t*‐test.Representative images of HeLa cells expressing ^mt^GFP transfected with mock or Lipin1^WT^ and treated for 3 h with vehicle, Tg (500 nM) or cycloheximide (CHX; 50 μg/ml). The inset shows twofold magnification of the image centered on the asterisk. Scale bars, 5 μm.Quantification of fragmented (red), tubular (yellow), or elongated (green) mitochondria from the images shown in (D). Error bars show SEM for *n* = 7 independent experiments. *P*‐value reflects comparisons of elongated (green) or fragmented (red) mitochondria populations for the indicated conditions. ***P < 0.005 for two‐way ANOVA (red indicates comparison between fragmented mitochondria fractions; green indicates comparisons between elongated mitochondria fractions).

**Figure EV2:**
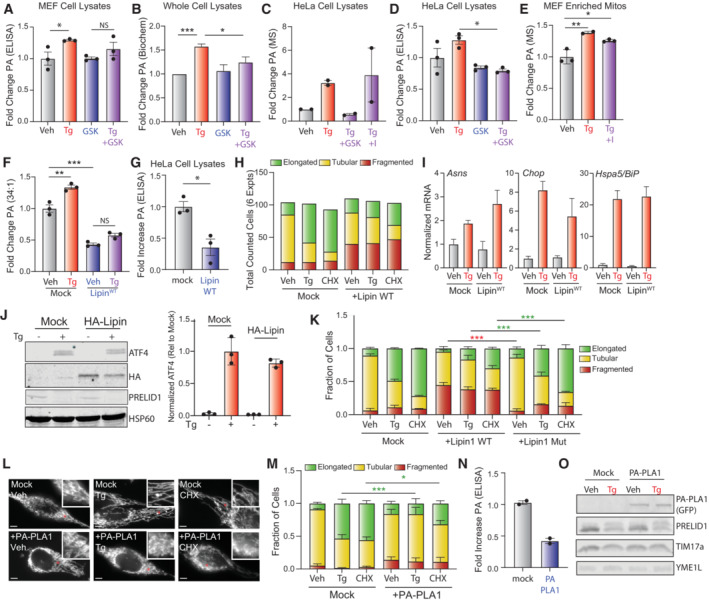
Supplement to Fig 2. Overexpression of PA lipases inhibits ER stress‐induced mitochondrial elongation A, B
Relative phosphatidic acid (PA), measured by ELISA (A) or biochemical assay (B) in whole cell extracts isolated from MEF cells treated for 3 h with vehicle, thapsigargin (Tg, 500 nM), and/or GSK2656157 (1 μM). Error bars show SEM for *n* = 3 biological replicates *P < 0.05 for unpaired *t*‐test (A) or *P < 0.05 ***P < 0.005 for paired *t*‐test (B).C
Relative PA, measured by untargeted mass spectrometry, in lysates prepared from HeLa cells treated for 3 h with vehicle, thapsigargin (Tg, 500 nM), GSK2656157 (GSK; 1 μM), or ISRIB (I; 200 nM), as indicated. Error bars show SEM for *n* = 2 biological replicates.D
Relative PA, measured by ELISA, in lysates prepared from HeLa cells treated for 3 h with vehicle, thapsigargin (Tg, 500 nM), and/or GSK2656157 (GSK; 1 μM). Error bars show SEM for *n* = 3 biological replicates and *P < 0.05 for one‐way ANOVA.E
Relative PA levels, measured by untargeted mass spectrometry, in mitochondrial enriched fractions from MEF cells treated for 3 h with vehicle, thapsigargin (Tg; 500 nM), and/or ISRIB (I; 0.2 μM). Error bars show SEM for *n* = 2–3 biological replicates. **P < 0.01, ***P < 0.005 for one‐way ANOVA.F
Normalized relative abundance of PA 34:1, as measured by targeted MS, in lysates of HeLa cells expressing mock or Lipin^WT^ and treated for 3 h with vehicle or thapsigargin (Tg; 500 nM), as indicated. Error bars show SEM for *n* = 3 biological replicates. **P < 0.01, ***P < 0.005 for one‐way ANOVA.G
Relative PA, measured by ELISA, in lysates of HeLa cells expressing mock or Lipin^WT^ and treated for 3 h with vehicle or thapsigargin (Tg; 500 nM), as indicated. Error bars show SEM for *n* = 3 biological replicates. *P < 0.05 for unpaired *t*‐test.H
Total counted cells for qualitative analysis of mitochondrial morphology for Fig 2D and E.I
Expression, measured by qPCR, of *Asns*, *Chop*, and *Hspa5/BiP* in HeLa cells expressing mock or Lipin^WT^ treated for 3 h with vehicle or thapsigargin (Tg, 500 nM). Error bars show ±95% confidence interval for *n* = 3 technical replicates.J
Immunoblot lysates prepared form HeLa cells expressing mock or Lipin^WT^ treated for 3 h with vehicle or thapsigargin (Tg; 500 nM). Note that the Lipin construct is HA tagged allowing detection with the HA antibody. Quantification of ATF4 from three different experiments is also shown.K
Quantification of fragmented (red), tubular (yellow), or elongated (green) mitochondria from HeLa cells transfected with either mock, Lipin^WT^ and a catalytically inactive lipin1 mutant (Lipin^mut^) treated for 3 h with vehicle, thapsigargin (Tg; 500 nM) or cycloheximide (CHX; 50 μg/ml). Error bars show SEM for *n* = 3 independent experiments. *P*‐value reflects comparisons of elongated (green) mitochondria populations for the indicated conditions. ***P < 0.005 for two‐way ANOVA.L
Representative images of HeLa cells expressing ^mt^GFP transfected with mock or GFP‐tagged PA‐PLA1 then treated for 3 h with thapsigargin (Tg; 500 nM) or cycloheximide (CHX; 50 μg/ml). The inset shows 2‐fold magnification of the image centered on the asterisk. Scale bars, 5 μm. Note that the presence of GFP on PA‐PLA1 did not influence our ability to monitor mitochondrial morphology in these cells.M
Quantification of fragmented (red), tubular (yellow), or elongated (green) mitochondria from the images shown in (L). Error bars show SEM for *n* = 3 independent experiments. *P < 0.05, ***P < 0.005 for two‐way ANOVA (green indicates comparisons between elongated mitochondria fractions).N
Relative PA, measured by ELISA, in lysates of HeLa cells expressing mock or PA‐PLA1. Error bars show SEM for *n* = 2 biological replicates.O
Immunoblot of lysates prepared from HeLa cells expressing mock or PA‐PLA1 treated for 3 h with vehicle or thapsigargin (Tg; 500 nM). Source data are available online for this figure.

Mitochondrial elongation can be induced by the accumulation of saturated PA on the OMM through mechanisms including PA‐dependent inhibition of the pro‐fission GTPase DRP1 (Baba *et al*, 2014; Ha & Frohman, 2014; Adachi *et al*, 2016; Kameoka *et al*, 2018; Acoba *et al*, 2020). PERK was previously shown to increase cellular PA during ER stress through a mechanism dependent on PERK kinase activity but independent of signaling downstream of eIF2α phosphorylation (Bobrovnikova‐Marjon *et al*, 2012). We found that treatment with Tg increases PA in mitochondria‐enriched fractions and whole‐cell extracts from both MEF or HeLa cells using mass spectrometry, biochemical assays, and ELISA (Figs 2A–C and EV2A–F). Phosphatidylcholine (PC) was not affected in enriched mitochondria (Fig 2A). Co‐treatment with the PERK inhibitor GSK2656157, a compound that directly inhibits PERK kinase activity (Fig 1A; Axten *et al*, 2013), reduced Tg‐dependent increases of PA in both MEF and HeLa cells (Figs 2B and EV2A–D). This indicates that ER stress‐dependent increases in PA require PERK kinase activity, as previously reported (Bobrovnikova‐Marjon *et al*, 2012). However, co‐treatment of MEFs with Tg and ISRIB, a compound that blocks PERK signaling downstream of eIF2α phosphorylation (Fig 1A; Sidrauski *et al*, 2013), did not appear to mitigate ER stress induced PA increases in either mitochondria enriched fractions or whole‐cell extracts (Figs 2B, and EV2B and E). This is consistent with previous results suggesting that ER stress increases PA through a mechanism selectively dependent on PERK kinase activity, but not signaling downstream of eIF2α phosphorylation (Bobrovnikova‐Marjon *et al*, 2012).

We next determined the dependence of PERK‐regulated mitochondrial elongation on PA by monitoring mitochondrial morphology in Tg‐treated HeLa cells co‐overexpressing ^mt^GFP and Lipin1—a cytosolic PA lipase that catalyzes the conversion of PA to diacylglycerol (DAG; Baba *et al*, 2014; Tatsuta & Langer, 2017; Kameoka *et al*, 2018; Tamura *et al*, 2020). We showed that Lipin1 overexpression reduced cellular PA and prevented Tg‐dependent increases of PA (Figs 2C, and EV2F and G). Overexpression of wild‐type Lipin1 increased basal mitochondrial fragmentation and inhibited Tg‐induced mitochondrial elongation (Figs 2D and E, and EV2H). Similar results were observed in cells treated with CHX. Lipin1 overexpression did not significantly impact the expression of ATF4 target genes (e.g., *Asns* and *Chop*) or increases of ATF4 protein in Tg‐treated cells (Fig EV2I and J). Furthermore, overexpression of a catalytically inactive Lipin1 did not influence basal mitochondrial morphology or mitochondrial elongation induced by Tg or CHX (Fig EV2K). Overexpression of PA‐PLA1—a cytosolic lipase that converts PA to lysophosphatidic acid (LPA; Baba *et al*, 2014)—similarly inhibited mitochondrial elongation in cells treated with Tg or CHX without impacting other aspects of PERK signaling (Fig EV2L–O). The sensitivity of Tg‐ and CHX‐induced mitochondrial elongation to PA‐PLA1 also suggests that this process is not mediated through increased LPA—a phospholipid that promotes mitochondrial elongation during starvation through a MTCH2‐dependent mechanism (Labbe *et al*, 2021). Collectively, our results show that depletion of PA afforded by overexpression of two distinct PA lipases blocks ER stress‐induced mitochondrial elongation, implicating PA in this process.

### ER stress prevents DRP1‐dependent mitochondrial fragmentation

**Figure EV3:**
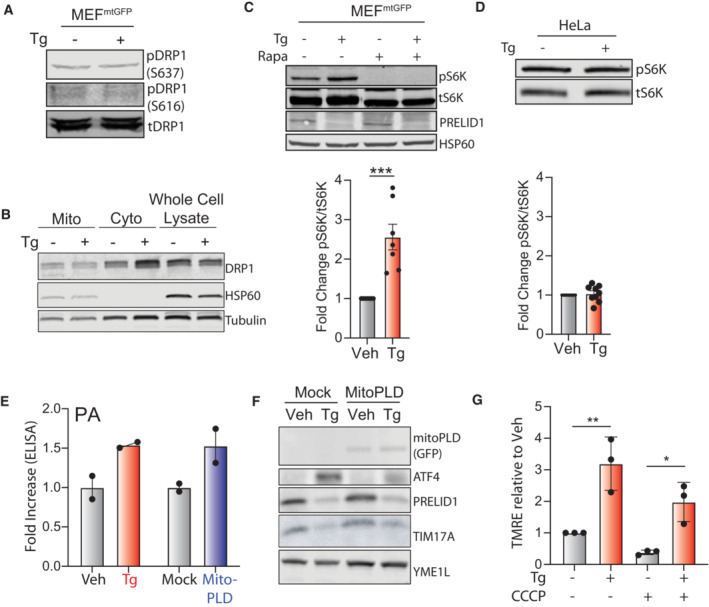
Supplement to Fig 3. ER stress‐induced mitochondrial elongation inhibits Ionomycin‐induced mitochondrial fragmentation Immunoblot of lysates prepared from MEF^mtGFP^ cells treated for 3 h with thapsigargin (Tg; 500 nM).Immunoblot of mitochondrial fractions, cytosolic fractions, or whole cell lysates from MEF^mtGFP^ cells treated for 3 h with thapsigargin (Tg; 500 nM).Immunoblot of lysates prepared from MEF^mtGFP^ cells treated for 3 h with thapsigargin (Tg; 500 nM) and/or rapamycin (Rapa; 10 μM). Quantification of pS6K normalized to tS6K is shown. Error bars show SEM for *n* = 7 independent experiments. ***P < 0.005 for paired *t*‐test.Immunoblot of lysates prepared from HeLa cells treated for 3 h with vehicle or thapsigargin (Tg; 500 nM). Quantification of pS6K normalized to tS6K is shown. Error bars show SEM for *n* = 9 independent experiments.Phosphatidic acid (PA), measured by ELISA, in HeLa cells treated with thapsigargin (Tg; 500 nM, 3 h) or expressing mock or mitoPLD. Error bars show SEM for *n* = 2 biological replicates. Individual replicates are shown.Immunoblot of lysates prepared from HeLa cells transfected with mock or mitoPLD^GFP^ and treated for 3 h with vehicle or thapsigargin (Tg; 500 nM). Note mitoPLD^GFP^ is tagged with GFP allowing detection of this protein with the GFP antibody.Mitochondrial polarization, measured by TMRE fluorescence, in MEF cells pre‐treated for 3 h with thapisgargin (Tg; 500 nM) then challenged for 30 min with CCCP (10 μM). Error bars show SEM for *n* = 3 biological replicates. *P < 0.05, **P < 0.01, ***P < 0.005 for one‐way ANOVA. Source data are available online for this figure.

Mitochondrial elongation can be induced in response to stress through mechanisms involving posttranslational regulation of the pro‐fission GTPase DRP1. DRP1 phosphorylation at residue S637 promotes mitochondrial elongation by inhibiting DRP1 GTPase activity, while the pro‐fission phosphorylation of DRP1 at S616 increases DRP1 localization to mitochondria and subsequent activity (Chang & Blackstone, 2007; Taguchi *et al*, 2007; Kar *et al*, 2017). Pharmacologic mTOR inhibition can induce mitochondrial elongation through a mechanism involving both increased DRP1 phosphorylation at S637 and reduced phosphorylation at S616 (Morita *et al*, 2017). However, as reported previously (Lebeau *et al*, 2018), Tg did not influence DRP1 phosphorylation at either S637 or S616 (Fig EV3A) or alter the amount of DRP1 enriched in mitochondrial fractions from MEF^mtGFP^ cells (Fig EV3B). PERK‐dependent increases in PA can activate mTOR during ER stress (Bobrovnikova‐Marjon *et al*, 2012). Consistent with this, we observe Tg‐dependent increases in mTOR‐dependent S6K phosphorylation in MEF^mtGFP^ cells (Fig EV3C). However, despite increasing PA and promoting mitochondrial elongation, Tg did not increase S6K phosphorylation in HeLa cells (Fig EV3D). These results suggest that PERK‐dependent alterations in mTOR activity are unlikely to be primary contributors to ER stress induced mitochondrial elongation across cell types.

**Figure 3:**
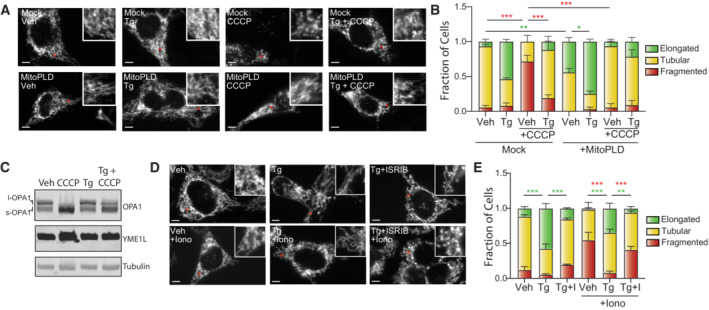
ER stress‐induced mitochondrial elongation inhibits ionomycin‐induced mitochondrial fragmentation Representative images of HeLa cells expressing ^mt^GFP transfected with mock or mitoPLD^GFP^ pretreated for 3 h with vehicle or thapsigargin (Tg; 500 nM) and then challenged with CCCP (20 μM) for 30 min. Note the expression of mitoPLD^GFP^ did not impair our ability to accurately monitor mitochondrial morphology in these cells.Quantification of fragmented (red), tubular (yellow), or elongated (green) mitochondria from the images shown in (A). Error bars show SEM for *n* = 3 independent experiments. *P < 0.05, **P < 0.01, ***P < 0.005 for two‐way ANOVA (red indicates comparison between fragmented mitochondria fractions; green indicates comparisons between elongated mitochondria fractions).Immunoblot of lysates prepared from MEF^mtGFP^ cells pre‐treated for 3 h with vehicle or Tg (500 nM) and then challenged with CCCP (20 μM) for 30 min.Representative images of MEF^mtGFP^ cells pre‐treated for 3 h with vehicle, thapsigargin (Tg; 500 nM), or Tg and ISRIB (I; 0.2 μM) then challenged with vehicle or ionomycin (Iono; 1 μM) for 30 min. The inset shows two‐fold magnification of the image centered on the asterisk. Scale bars, 5 μm.Quantification of fragmented (red), tubular (yellow), or elongated (green) mitochondria from the images shown in (D). Error bars show SEM for *n* = 3 independent experiments. **P < 0.01, ***P < 0.005 for two‐way ANOVA (red indicates comparison between fragmented mitochondria fractions; green indicates comparisons between elongated mitochondria fractions). Source data are available online for this figure.

Accumulation of PA on the OMM can also promote mitochondrial elongation by inhibiting DRP1 activity (Adachi *et al*, 2016). This was previously demonstrated by showing that genetically increasing PA on the OMM by overexpressing mitoPLD—an OMM lipase that converts cardiolipin to PA—basally increased mitochondrial elongation and inhibited DRP1‐dependent mitochondrial fragmentation induced by the uncoupler carbonyl cyanide m‐chlorophenylhydrazone (CCCP; Li *et al*, 2015; Adachi *et al*, 2016). Consistent with this, we observed that mitoPLD overexpression in HeLa cells increased basal mitochondrial elongation and inhibited CCCP‐induced mitochondrial fragmentation (Fig 3A and B). We found that mitoPLD overexpression increased cellular PA to levels similar to that observed in Tg‐treated cells and did not significantly influence PERK signaling (Fig EV3E and F). Pretreatment with Tg also reduced CCCP‐induced mitochondrial fragmentation. However, Tg pretreatment inhibited CCCP‐induced proteolytic cleavage of the inner membrane GTPase OPA1 (Fig 3C)—a biological process upstream of DRP1 in mitochondrial fragmentation induced by membrane uncoupling (Mishra & Chan, 2016; Jones *et al*, 2017; Chan, 2020; Fenton *et al*, 2020; Giacomello *et al*, 2020; Sabouny & Shutt, 2020). This appears to result from Tg‐dependent increases in mitochondrial membrane polarity (Fig EV3G), preventing efficient uncoupling in CCCP‐treated cells and precluding our ability to determine whether Tg pretreatment directly impairs DRP1 activity under these conditions.

To circumvent this problem, we monitored mitochondria morphology in MEF^mtGFP^ cells pretreated with Tg and then challenged with ionomycin—a Ca^2+^ ionophore that increases cytosolic Ca^2+^ (Ji *et al*, 2015; Mishra & Chan, 2016; Chan, 2020; Fenton *et al*, 2020; Giacomello *et al*, 2020; Sabouny & Shutt, 2020). Increases in cytosolic Ca^2+^ induced by short (< 30 min) treatment with ionomycin promotes DRP1‐dependent mitochondrial fragmentation through a mechanism independent of membrane uncoupling or OPA1 processing (Ji *et al*, 2015). Pretreatment for 3 h with Tg—a time point sufficient to increase PA and induce mitochondrial elongation—inhibits ionomycin‐induced mitochondrial fragmentation (Fig 3D and E). This inhibition is reversed by co‐treatment with ISRIB, a small molecule that blocks eIF2α phosphorylation‐dependent signaling downstream of PERK (Fig 1A). This indicates that this inhibition of ionomycin‐induced fragmentation can be attributed to PERK signaling and not dysregulation of intracellular Ca^2+^ induced by the combined treatment of Tg and ionomycin. These results are consistent with a model whereby ER stress promotes mitochondrial elongation through a mechanism involving PA‐dependent inhibition of DRP1‐mediated fission, as reported previously for mitoPLD overexpression (Adachi *et al*, 2016).

### PERK signaling leads to reductions in the intramitochondrial PA transporter PRELID1 during ER stress

**Figure 4:**
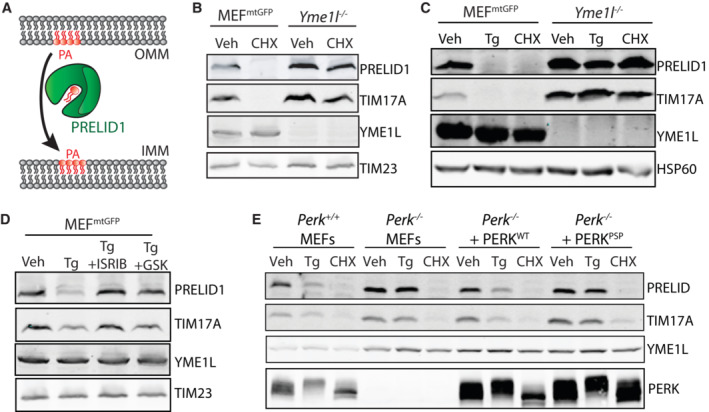
ER stress reduces PRELID1 through a YME1L‐dependent mechanism downstream of PERK‐dependent translational attenuation Illustration showing the PRELID1‐dependent trafficking of PA from the outer to inner mitochondrial membranes (OMM and IMM, respectively).Immunoblot of lysates prepared from MEF^mtGFP^ cells and *Yme1l*‐deficient MEF^mtGFP^ cells treated for 3 h with vehicle or cycloheximide (CHX; 50 μg/ml).Immunoblot of lysates prepared from MEF^mtGFP^ cells and *Yme1l*‐deficient MEF^mtGFP^ cells treated for 3 h with vehicle, thapsigargin (Tg; 500 nM) or cycloheximide (CHX; 50 μg/ml).Immunoblot of lysates prepared from MEF^mtGFP^ cells treated for 3 h with vehicle, Tg (500 nM), Tg and ISRIB (0.2 μM), or Tg and GSK2656157 (GSK; 1 μM).Immunoblot of lysates prepared from *Perk* ^+/+^ MEFs, *Perk* ^−/−^ MEFs, or *Perk* ^−/−^ MEFs transfected with wild‐type PERK^WT^ or the PSP‐associated PERK allele (PERK^PSP^) treated for 6 h with thapsigargin (Tg; 500 nM) or cycloheximide (CHX; 50 μg/ml). Source data are available online for this figure.

**Figure EV4:**
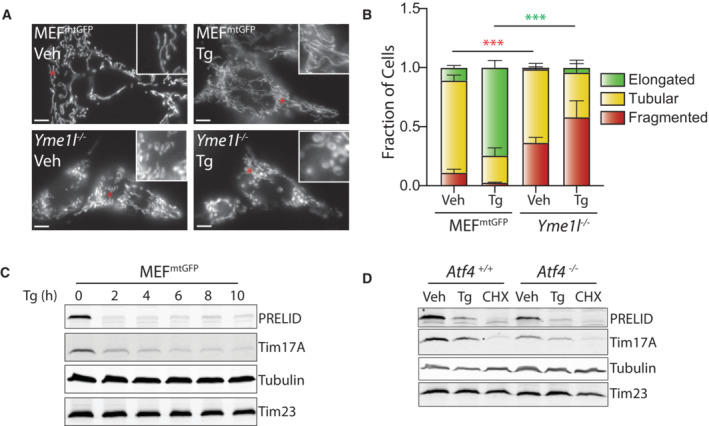
Supplement to Fig 4. ER stress reduces PRELID1 through a YME1L‐dependent mechanism downstream of PERK‐dependent translational attenuation Representative images of MEF^mtGFP^ cells and *Yme1l*‐deficient MEF^mtGFP^ cells treated for 6 h with thapsigargin (Tg; 500 nM). The inset shows twofold magnification of the image centered on the asterisk. Scale bars, 5 μm.Quantification of fragmented (red), tubular (yellow), or elongated (green) mitochondria from the images shown in (A). Error bars show SEM for *n* = 7 independent experiments. *P*‐value reflects comparisons of elongated (green) or fragmented (red) mitochondria populations for the indicated conditions. ***P < 0.005 for two‐way ANOVA (red indicates comparison between fragmented mitochondria fractions; green indicates comparisons between elongated mitochondria fractions).Immunoblot of lysates prepared from MEF^mtGFP^ cells treated with thapsigargin (Tg; 500 nM) for the indicated time.Immunoblot of lysates prepared from *Atf4* ^+/+^ and *Atf4* ^−/−^ MEFs treated with thapsigargin (Tg; 500 nM) or CHX (50 μg/ml) for 3 h. Source data are available online for this figure.

ER stress induced mitochondrial elongation is inhibited by shRNA‐depletion of *YME1L* in HeLa cells (Lebeau *et al*, 2018). We further confirmed the dependence of Tg‐induced mitochondrial elongation on YME1L in MEF^mtGFP^ cells where *Yme1l* was deleted by CRISPR (Fig EV4A and B). PRELID1, an intermembrane space protein that transports PA from the OMM to the IMM (Fig 4A; Tatsuta & Langer, 2017; Tamura *et al*, 2020), is a known substrate of YME1L (Potting *et al*, 2010; Tamura *et al*, 2012; MacVicar *et al*, 2019). Interestingly, PRELID1 is a short‐lived protein whose levels are highly sensitive to translational attenuation (Li *et al*, 2021). Consistent with this, PRELID1 levels are reduced in MEF^mtGFP^ cells treated with CHX for 3 h (Fig 4B). This CHX‐dependent reduction in PRELID1 was blocked in *Yme1l*‐deficient cells (Fig 4B), confirming that PRELID1 is degraded by YME1L under these conditions. Identical results were observed for TIM17A, another short‐lived mitochondrial protein degraded by YME1L downstream of translation inhibition (Fig 4B; Rainbolt *et al*, 2013).

The sensitivity of PRELID1 to reductions in protein translation suggests that this protein could be decreased in response to PERK‐dependent translational attenuation. As expected, PRELID1 was rapidly decreased in MEF^mtGFP^ cells treated with the ER stressor Tg (Fig EV4C). Tg‐dependent reductions in PRELID1 were inhibited in cells deficient in *Yme1l*, indicating that YME1L was required for this process (Fig 4C). Co‐treatment with either the PERK kinase inhibitor GSK2656157 or the PERK signaling inhibitor ISRIB (Fig 1A) blocked Tg‐dependent reductions in PRELID1 (Fig 4D). Similar results were observed for TIM17A. Tg‐dependent reductions in PRELID1 and TIM17A were also inhibited in *Perk* ^−/−^ MEFs (Fig 4E). Reconstitution of *Perk* ^−/−^ cells with PERK^WT^, but not hypomorphic PERK^PSP^, restored Tg‐induced degradation of these proteins. Importantly, CHX reduced PRELID1 and TIM17A in all genotypes, confirming that these proteins remain sensitive to translational attenuation even when PERK signaling is impaired (Fig 4E). Tg‐dependent reductions in PRELID1 were not inhibited in cells deficient in *Atf4* (Fig EV4D; Harding *et al*, 2003), a primary upstream transcription factor in the PERK pathway (Fig 1A). This indicates that this phenotype is independent of PERK‐regulated transcriptional signaling. Collectively, these results suggest that PRELID1, like TIM17A (Rainbolt *et al*, 2013), is reduced during ER stress through a YME1L‐dependent mechanism downstream of PERK‐dependent translational attenuation.

### PERK‐dependent PRELID1 degradation remodels mitochondrial membranes during ER stress

**Figure EV5:**
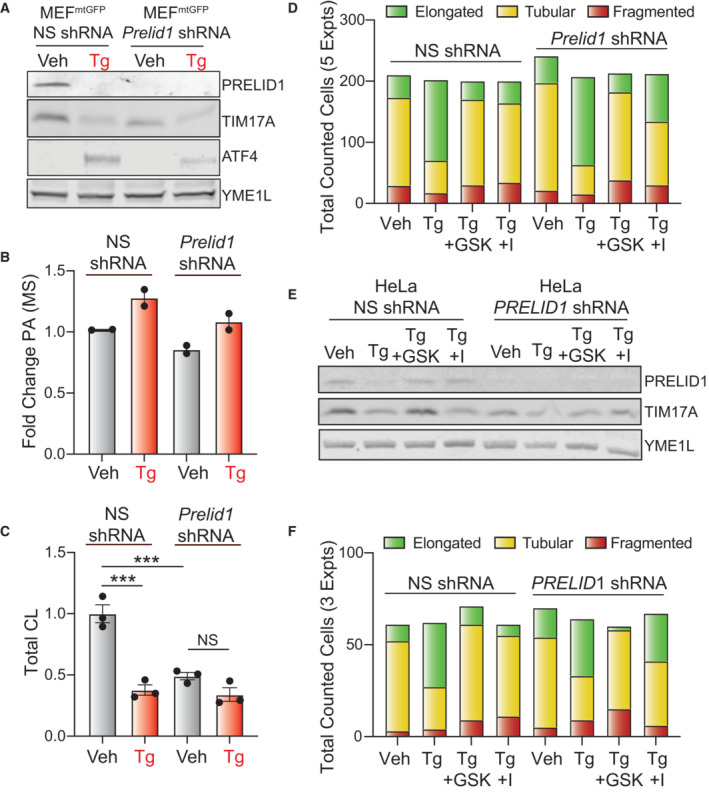
Supplement to Fig 5. Reductions in PRELID1 contribute to ER stress‐induced mitochondrial elongation Immunoblot of lysates from MEF^mtGFP^ cells expressing non‐silencing (NS) or *Prelid1* shRNA treated for 3 h with vehicle or thapsigargin (Tg; 500 nM).Relative amounts of phosphatidic acid (PA), measured by untargeted mass spectrometry, in lysates prepared from MEF^mtGFP^ cells expressing non‐silencing (NS) or *Prelid1* shRNA treated for 3 h with vehicle or thapsigargin (Tg; 500 nM). Error bars show SEM for *n* = 2 independent replicates.Normalized relative abundance of total cardiolipin (CL) species measured by targeted mass spectrometry in isolated mitochondria prepared from MEF^mtGFP^ cells expressing non‐silencing (NS) or *Prelid1* shRNA treated for 3 h with vehicle or thapsigargin (Tg; 500 nM). Error bars show SEM for *n* = 3 biological replicates. ***P < 0.005 for one‐way ANOVA.Total counted cells for qualitative analysis of mitochondrial morphology in Fig 5A and B.Immunoblot of lysates prepared from HeLa cells expressing non‐silencing (NS) or *PRELID1* shRNA and treated for 3 h with vehicle or thapsigargin (Tg, 500 nM), GSK2656157 (10 μM), and/or ISRIB (I; 2 μM), as indicated.Total counted cells for qualitative analysis of mitochondrial morphology for Fig 5C and D. Source data are available online for this figure.

PRELID1 traffics PA from the outer to inner mitochondrial membrane, where it serves as a precursor to the formation of cardiolipin (Potting *et al*, 2010; Tamura *et al*, 2012; Tatsuta & Langer, 2017). Thus, reductions in PRELID1 should decrease cardiolipin. To test this, we shRNA‐depleted *Prelid1* from MEF^mGFP^ cells and monitored cardiolipin in isolated mitochondria in the presence or absence of ER stress. We confirmed efficient PRELID1 knockdown by immunoblotting (Fig EV5A). Importantly, *Prelid1* depletion did not alter Tg‐induced reductions of TIM17A or increases of ATF4. Furthermore, Tg‐dependent increases in PA were observed in *Prelid1*‐depleted MEF^mtGFP^ cells (Fig EV5B). These results indicate that loss of PRELID1 does not impair PERK signaling in these cells. *Prelid1* depletion reduced cardiolipin in mitochondria isolated from MEF^mtGFP^ cells (Fig EV5C). Treatment of MEF^mtGFP^ cells expressing nonsilencing shRNA with Tg for 3 h also reduced cardiolipin to levels similar to those observed in *Prelid1*‐deficient cells. However, Tg did not further reduce cardiolipin in *Prelid*‐depleted cells. These results are consistent with a model whereby ER stress‐dependent reductions in PRELID1 limit PA trafficking across the inner mitochondrial membrane and contribute to reductions in cardiolipin during acute ER stress.

**Figure 5:**
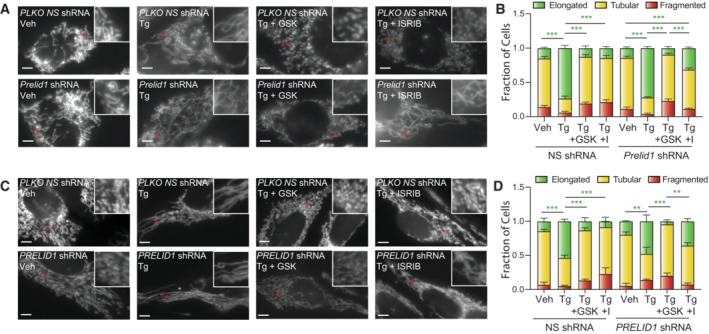
Reductions in PRELID1 contribute to ER stress‐induced mitochondrial elongation Representative images of MEF^mtGFP^ cells expressing non‐silencing (NS) or *Prelid1* shRNA treated for 3 h with thapsigargin (Tg; 500 nM) and either GSK2656157 (GSK; 1 μM) or ISRIB (I; 0.2 μM), as indicated. The inset shows 2‐fold magnification of the image centered on the asterisk. Scale bars, 5 μm.Quantification of fragmented (red), tubular (yellow), or elongated (green) mitochondria from the images shown in (A). Error bars show SEM for *n* = 5 independent experiments. ***P < 0.005 for two‐way ANOVA (green indicates comparisons between elongated mitochondria fractions).Representative images of HeLa cells expressing ^mt^GFP and non‐silencing (NS) or *PRELID1* shRNA treated for 3 h with thapsigargin (Tg; 500 nM) and either GSK2656157 (GSK; 1 μM) or ISRIB (I; 0.2 μM), as indicated. The inset shows twofold magnification of the image centered on the asterisk. Scale bars, 5 μm.Quantification of fragmented (red), tubular (yellow), or elongated (green) mitochondria from the images shown in (C). Error bars show SEM for *n* = 3 independent experiments. *P*‐value reflects comparisons of elongated (green) mitochondria populations for the indicated conditions. **P < 0.01; ***P < 0.005 for two‐way ANOVA (green indicates comparisons between elongated mitochondria fractions).

In combination with ER stress‐dependent increases in PA (Figs 2A–C and EV2A–F), reductions in PRELID1‐mediated PA trafficking across mitochondrial membranes should lead to the accumulation of PA on the OMM where it could promote mitochondrial elongation by inhibiting mitochondrial fission (Adachi *et al*, 2016). To test this, we monitored mitochondrial morphology in *Prelid1*‐depleted MEF^mtGFP^ cells in the presence and absence of Tg. Interestingly, *Prelid1* depletion did not basally influence mitochondrial morphology or inhibit Tg‐induced mitochondrial elongation (Figs 5A and B, and EV5D). This indicates that reduction of PRELID1, on its own, is not sufficient to increase mitochondrial elongation, likely reflecting the importance of PERK kinase‐dependent increases in PA in this process (Bobrovnikova‐Marjon *et al*, 2012). Consistent with this model, co‐treatment with the PERK kinase inhibitor GSK2656157 blocked Tg‐induced mitochondrial elongation in *Prelid1*‐deficient cells (Figs 5A and B, and EV5D). However, we found that *Prelid1* depletion partially rescued the Tg‐induced mitochondrial elongation in cells co‐treated with ISRIB—a compound that blocks PERK‐dependent PRELID1 degradation (Fig 4D), but not PERK kinase‐dependent increases in PA (Figs 2B, and EV2B and E). Co‐treatment with ISRIB completely blocked Tg‐induced mitochondrial elongation in MEF^mtGFP^ cells expressing non‐silencing shRNA (Figs 5A and B, and EV5D). Identical results were observed in HeLa cells depleted of *PRELID1* and treated with Tg, GSK2656157, and/or ISRIB (Figs 5C and D, and EV5E and F). These results indicate PERK‐dependent reductions in PRELID1 contribute to the mitochondrial elongation observed during ER stress.

## Discussion

Mitochondrial elongation is an adaptive mechanism that protects mitochondria in response to diverse pathologic insults (Rambold *et al*, 2011; Gomes *et al*, 2011a, 2011b; Lee *et al*, 2012, 2014; Lebeau *et al*, 2018; Labbe *et al*, 2021; Oshima *et al*, 2021). Numerous mechanisms have been shown to promote mitochondrial elongation in response to different types of stress. For example, the accumulation of lysophosphatidic acid (LPA) on the outer mitochondrial membrane increases mitochondrial elongation through a MTCH2‐dependent mechanism during starvation (Labbe *et al*, 2021). Alternatively, HDAC6‐dependent deacetylation of pro‐fusion GTPase MFN1 increases mitochondrial length during glucose deprivation by enhancing the activity of organellar fusion pathways (Lee *et al*, 2014). Furthermore, PKA‐dependent phosphorylation or PARKIN‐dependent ubiquitination of the pro‐fission GTPase DRP1 inhibits mitochondrial fission and promotes mitochondrial elongation under a variety of different conditions (Chang & Blackstone, 2007; Cribbs & Strack, 2007; Oshima *et al*, 2021). Despite these differences in mechanism, mitochondrial elongation similarly functions to prevent premature fragmentation, regulate mitochondria respiratory chain activity, and promote cell survival in response to diverse pathologic insults, including ER stress (Chang & Blackstone, 2007; Cribbs & Strack, 2007; Rambold *et al*, 2011; Gomes *et al*, 2011a, 2011b; Lee *et al*, 2012, 2014; Lebeau *et al*, 2018; Labbe *et al*, 2021; Oshima *et al*, 2021).

**Figure 6:**
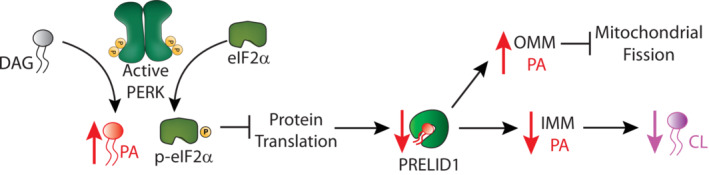
Proposed mechanism for PERK‐dependent regulation of mitochondrial PA during ER stress In response to ER stress, PERK is activated, leading to an increase in total and mitochondrial PA (left) – a process that was previously suggested to result from PERK kinase‐dependent phosphorylation of diacylglycerol (DAG; Bobrovnikova‐Marjon *et al*, 2012). YME1L degrades the intramitochondrial PA transporter PRELID1 downstream of PERK‐dependent translational attenuation, limiting the trafficking of PA to the inner mitochondrial membrane (IMM). This both decreases the population of PA in the IMM available for conversion to cardiolipin (CL) and promotes PA accumulation on the outer mitochondrial membrane (OMM) where it can promote mitochondrial elongation by inhibiting mitochondrial fission through mechanisms such as direct inhibition of DRP1 (Adachi *et al*, 2016).

ER stress promotes mitochondrial elongation through a process regulated by the PERK arm of the UPR (Lebeau *et al*, 2018). Here, our results suggest a model whereby PERK signaling promotes mitochondrial elongation through a mechanism involving PERK‐dependent remodeling of mitochondrial membrane PA (Fig 6). Previously, ER stress was shown to increase cellular PA through a mechanism dependent on PERK kinase activity, but not eIF2α phosphorylation (Bobrovnikova‐Marjon *et al*, 2012). This was suggested to involve direct, PERK‐dependent phosphorylation of diacylglycerol (DAG; Fig 6; Bobrovnikova‐Marjon *et al*, 2012); however, other mechanisms could also contribute to the PERK kinase activity‐dependent increase in PA. Our results support the preferential dependence of ER stress induced increases of PA on PERK kinase activity, showing that the PERK kinase inhibitor GSK2656157 reduces Tg‐dependent increases of PA, while ISRIB, a compound that inhibits PERK signaling downstream of eIF2α phosphorylation (Fig 1A), does not significantly impact Tg‐dependent increases of PA.

Our findings that ISRIB blocks Tg induced mitochondrial elongation suggest that PERK‐dependent mitochondrial elongation involves other mechanisms regulated downstream of eIF2α phosphorylation. To account for this, we demonstrate that ER stress‐dependent increases in mitochondrial elongation also involves reductions in the intramitochondrial PA trafficking protein PRELID1 (Fig 6). We show that PRELID1 is a short‐lived mitochondrial protein that is degraded through a YME1L‐dependent mechanism downstream of eIF2α phosphorylation‐dependent translation attenuation. We implicated PERK‐dependent reductions of PRELID1 in ER stress induced mitochondrial elongation by showing that genetic depletion of PRELID1 partially rescues ER stress induced mitochondrial elongation in cells co‐treated with the PERK signaling inhibitor ISRIB, but not the PERK kinase inhibitor GSK2656157. This highlights an important role for PERK‐dependent reductions of PRELID1 in the adaptive remodeling of mitochondrial membrane PA observed during ER stress.

The combination of PERK‐dependent increases in total PA and YME1L‐dependent decreases of PRELID1 should increase PA on the OMM during conditions of ER stress. Previous studies have shown that increases in OMM PA promote mitochondrial elongation through multiple mechanisms including direct inhibition of the pro‐fission GTPase DRP1 (Fig 6; Adachi *et al*, 2016). Consistent with an important role for OMM PA in ER stress‐induced mitochondrial elongation, overexpression of two different cytosolic PA lipases, Lipin1 and PA‐PLA1, block mitochondrial elongation observed in Tg‐treated cells. Furthermore, we demonstrate that pretreatment with the ER stressor Tg inhibits DRP1‐dependent mitochondrial fragmentation in ionomycin‐treated cells. Collectively, these results support a model whereby PERK‐dependent increases in OMM PA promote mitochondrial elongation through a mechanism involving reductions in mitochondrial fission, potentially mediated through mechanisms such as the direct inhibition of the pro‐fission GTPase DRP1 (Fig 6).

PERK‐regulated translational and transcriptional signaling regulate diverse aspects of mitochondrial proteostasis and function. Our results provide insights into PERK‐dependent remodeling of mitochondria by demonstrating that signaling through this UPR pathway promotes adaptive remodeling of mitochondrial membrane PA to induce protective organelle elongation during ER stress. As we and others continue studying the impact of PERK signaling on mitochondrial biology, additional mitochondrial pathways regulated through PERK signaling will also likely be identified, further expanding our understanding of the critical role for this stress‐responsive signaling pathway in regulating mitochondria. Moving forward, it will also be interesting to define how different PERK‐dependent mitochondrial adaptations integrate to influence other aspects of mitochondrial function during conditions of stress. For example, recent cryo‐electron tomography results indicate that mitochondrial elongation correlates with cristae remodeling in ER stressed cells, suggesting that these changes to bulk mitochondrial morphology and ultrastructure may be coordinated (Barad *et al*, 2023).

The global importance of PERK in adapting mitochondria during ER stress also suggests that disruptions in this signaling could exacerbate mitochondrial dysfunction in disease. Genetic mutations in *EIF2AK3*, the gene that encodes PERK, are causatively associated with Wolcott–Rallison syndrome—a devastating disease characterized by early onset diabetes, skeletal deformities, and growth impairments (Delepine *et al*, 2000). Furthermore, a hypomorphic PERK haplotype is associated with tauopathies including progressive supranuclear palsy (PSP; Hoglinger *et al*, 2011; Stutzbach *et al*, 2013; Yuan *et al*, 2018). Interestingly, mitochondrial dysfunction has been implicated in all these disorders, suggesting that failure of PERK‐dependent mitochondrial regulation could be a contributing factor in disease pathogenesis. Consistent with this, we show that hypomorphic PSP‐associated PERK alleles disrupt adaptive PERK‐dependent mitochondrial elongation and YME1L‐dependent PRELID1 degradation. In contrast, chronic PERK activation is also implicated in the pathogenesis of numerous neurodegenerative diseases involving mitochondrial dysfunction such as AD and prion disease (Moreno *et al*, 2012; Halliday *et al*, 2015, 2017b; Radford *et al*, 2015; Bell *et al*, 2016). While the specific importance of PERK signaling on mitochondrial function in these diseases remains largely undefined, this suggests that PERK signaling, while adaptive during acute ER insults, could become detrimental to mitochondria in response to chronic ER insults. Further investigations will be required to determine the specific impact of altered PERK signaling on mitochondria regulation in the context of these diseases to define both the pathologic and potentially therapeutic implications of PERK activity on the mitochondrial dysfunction implicated in these disorders.

## Materials and Methods

### Cell culture, transfections, lentiviral transduction, and CRISPR deletion

MEF^mtGFP^ (a kind gift from Peter Schultz, TSRI; Wang *et al*, 2012), *Perk* ^+/+^ *and Perk* ^−/−^ MEFs (Harding *et al*, 2000), *Atf4* ^+/+^ and *Atf4* ^−/−^ MEFs (Harding *et al*, 2003; kind gifts from David Ron, Cambridge), HeLa cells (purchased from the ATCC), or HEK293T cells were cultured in DMEM (Corning‐Cellgro) supplemented with 10% fetal bovine serum (FBS; Omega Scientific), 2 mM L‐glutamine (GIBCO), 100 U/ml penicillin, and 100 mg/ml streptomycin (GIBCO). Cells were maintained at 37°C and 5% CO_2_. Nonessential amino acids (GIBCO) and 2‐mercaptoethanol (ThermoFisher) were added to culture media of *Atf4* ^+/+^ and *Atf4* ^−/−^ MEFs and *Perk* ^+/+^ and *Perk* ^+/+^ MEFs. HeLa cells were transfected by calcium phosphate precipitation, as previously described (Lebeau *et al*, 2018). MEF cells were transfected with MEF Avalanche Transfection Reagent (EZ Biosystems) according to the manufacturer's protocol. Lentivirus were prepared by transfecting HEK293T cells with pRSV‐rev (Addgene #12253), pMDL‐RRE (Addgene, #12251), pMD2.6 (Addgene #12259), and the indicated shRNA in the pLKO.1 vector (Sigma) using calcium phosphate precipitation. After 24 h, the transfection media was removed and replaced with complete DMEM and incubated overnight for viral production. Virus containing media was removed the following day and filtered with a 0.45‐μm syringe filter (Genessee Scientific). Polybrene (ThermoFisher) was added to the virus containing media at a concentration of 10 μg/ml and the media was then added to HeLa or MEF^mtGFP^ cells. Stable pools of cells expressing nonsilencing or gene‐specific knockdowns were then generated through selection with puromycin (3 mg/ml for MEF cells and 1 mg/ml for HeLa). Knockdown was confirmed by immunoblotting. *Yme1l* was deleted from MEF^mtGFP^ cells using CRISPR/Cas9. Briefly, cells were transfected with pSpCas9(BB)‐2A‐Puro (PX459; Addgene, #62988) containing sgRNA against *Yme1l* (GATCCAATATGAGATGTATGCCAAC AAACGTTGGCATACATCTCATATT) using MEF Avalanche, following manufacturers protocols. After transfection, cells were selected with puromycin and single clones were screened for YME1L depletion by qPCR and immunoblotting.

### Plasmids, shRNAs, and compounds

HA‐LIPIN1^WT^, HA‐LIPIN^Mut^, and mitoPLD‐GFP overexpression constructs were kind gifts from Hiromi Sesaki (Johns Hopkins) and described previously (Adachi *et al*, 2016). The PA‐PLA1‐GFP overexpression construct was purchased from Addgene (#162880). *Perk* ^*WT*^ and *Perk* ^*PSP*^ overexpression plasmids were kind gifts from Jonathan Lin (Stanford; Yuan *et al*, 2018). Plasmids containing shRNA were purchased from Sigma in the pLKO.1 vector: mouse *Prelid1* shRNA (TRCN0000345802), human *PRELID1* shRNA (TRCN0000130829). All compounds used in this study were purchased: thapsigargin (Tg; Fisher Scientific), GSK2656157 (BioVision Inc.), ISRIB (Sigma), CCCP (Sigma), rapamycin (Selleckchem), and ionomycin (Sigma).

### Fluorescence microscopy

HeLa cells transfected with ^mt^GFP or MEF^mtGFP^ cells were seeded at a density of 100,000 cells/well on glass‐bottom dishes (MatTek) coated with poly‐D‐lysine (Sigma) or rat tail collagen 1 (GIBCO). Cells were then treated as indicated and images were recorded with an Olympus IX71 microscope with 60× oil objective (Olympus), a Hamamatsu C8484 camera (Hamamatsu Photonics), and HCI image software (Hamamatsu Photonics). Quantification was performed by blinding the images and then scoring cells based on the presence of primarily fragmented, tubular, or elongated mitochondria, as before (Lebeau *et al*, 2018). At least three different researchers scored each set of images and these scores were averaged for each individual experiment and all quantifications shown were performed for at least three independent experiments quantifying a total of > 60 cells/condition across all experiments. The data were then prepared in PRISM (GraphPad, San Diego, CA) and plotted on a stacked bar plot to show the average morphology and standard error of the mean across all experiments. Statistical comparisons were performed using a two‐way ANOVA in PRISM, comparing the relative amounts of fragmented, tubular, or elongated mitochondria across different conditions.

### Phospholipid quantification

For untargeted MS analysis of PA, whole‐cell pellets were resuspended in 500 μl of a cold hypotonic buffer consisting of 1 mM PBS, pH 7.4. The material was then homogenized on ice using a glass Dounce homogenizer (30 strokes). The homogenized sample was centrifuged at 500 × *g* for 4 min then the supernatant was transferred to a 1.5‐ml microfuge tube and lyophilized overnight. The lyophilized material was weighed and normalized by total mass prior to performing a modified Bligh and Dyer extraction (Bligh & Dyer, 1959). The proceeding steps were carried out with glass pipettes and tubes to avoid plastic contamination. PA was extracted by the addition of 100 μl/mg of cold methanol containing an internal PA standard (Splash Lipidomix 330707, Avanti) at a dilution of (1:50), followed by 50 μl/mg of cold chloroform (CHCl_3_) with occasional vortex mixing. Milli‐Q H_2_0 containing 5 mM erythorbate was added at a volume of 50 μl/mg. The sample was agitated and centrifuged in glass test tubes at 200 × *g* for 10 min. The bottom phase was collected in a clean test tube, while the upper phase was re‐extracted two additional times with CH₃OH:CHCl_3_ (1:1, v/v) containing HCl at final concentration of 10 mM. The organic phases were combined and dried under vacuum to afford a lipid film that was stored at −80°C until MS analysis. Mitochondria‐enriched fractions were processed similar to whole‐cell samples except they were normalized via protein concentration as determined by the Pierce™ BCA Protein Assay kit (Thermo Scientific). In Brief, PA was extracted from the mitochondria enriched fractions using 10 μl/μg methanol, 5 μl/μg CHCl_3_ and 5 μl/μg milli‐Q H_2_0 containing 5 mM erythorbate as previously described above.

Extracted lipid samples and external standards (SPLASH Lipidomix 330707, 18:0 CL 710334p, Avanti) were removed from the −80°C freezer after drying and stored under nitrogen were resuspended in 100 μl of methanol. Negative mode LC–MS analysis was performed on an Agilent 6230 ESI‐TOF‐MS System calibrated with a reference solution at m/z 1,033.9881. A XBridge BEH C8 XP Column (Waters, 2.5 μm, 4.6 mm × 150 mm) was used at a flow rate of 500 μl/min, employing the following gradient: 30 to 100% solvent B over 30 min, 100% isocratic B for 10 min followed by a return to 30% B for 5 min. Solvents A consisted of MilliQ water and methanol (9:1, v:v). Solvents B consisted of acetonitrile: 2‐propanol (5:3, v:v), and both contained 10 mM Piperidine, 10 mM ammonium acetate (or 10 mM sodium acetate), and 0.1% formic acid. Prior to processing, raw.d files were converted to the open format mzXML using MSConvert software, which is part of the ProteoWizard software toolkit (Chambers *et al*, 2012). Mass detection was achieved using mzmine 2 wavelet algorithm, ADAP chromatogram builder and ADAP deconvolution, which are part of the MZmine 2 software package (Pluskal *et al*, 2010). Initial lipid identifications were achieved using lipidmaps database using [M − H]^−^ for PA with a m/z tolerance of 15 ppm, subsequently detected lipids were filtered out for further processing. The putative PA peaks were validated by aligning to the internal and external standards followed by graphical identification of PA lipids by plotting the Kendrick mass defect plot employing CH_2_ as the repeating unit. The quantification of all PA lipid classes was normalized based on the abundance of the internal standard PA (15:0–18:1‐d7‐PA), which factors in extraction efficiency and sample handling. Total PA levels were then normalized to vehicle for the indicated number of independent experiments.

Targeted lipidomics was performed on abundant lipid species for both PA and CL, (34:1) and (66:2, 68:2, 68:3), respectively. The relative abundance of these individual lipid species were quantified using a 1,260 Infinity II LC System (G6125BW) in selected ion monitoring (SIM) mode outfitted with an ESI source. All of the lipid species were observed as [M – H] ^−^ ions. Subsequently, the replicates were pooled together and injected onto a high‐resolution, time‐of‐flight (Q‐TOF) MS/MS (Agilent model 6546) using the identical RP platform as the targeted MS approach for the purpose of molecular identification. For the MS/MS PA, 34:1 was observed as a [M – H] ^−^ peak at 673.4802 with mass fragments of 281.2490, 255.2335, and 152.9963 m/z. For Cl 68:2, we observed the [M – H] ^−^ peak at 1,403.9983 with the MS/MS spectra comprising primarily of 673.4805, 417.2421, 281.2485, and 255.2324 m/z. The RP platform used was the same as previously described above for our untargeted approach except the mobile phase was changed to, 0–10 min 40% solvent B isocratic, 10–40 min 40–100% solvent B as a gradient. Solvent A is MilliQ water:methanol (1:1, v/v) and solvent B is methyl tert‐butyl ether (MTBE): 2‐propanol (1:4, v:v) both containing 2 mM ammonium acetate. To generate the required sensitivity for identification in the MS/MS we extracted the lipids as described previously with these notable exceptions. The dried cell lysate was comprised from a single 15‐cm tissue culture plate affording 5–6 mg of dried material per sample. The total cellular lipids of this material were extracted using four sequential extractions differing in solvent compositions. The first extraction was achieved by adding 200 μl MilliQ water, 400 μl methanol and 400 μl chloroform. The second subsequent extraction was performed by adding an additional 200 μl methanol and 400 μl chloroform to the remaining aqueous layer. The third extraction uses an addition of 500 μl MTBE, this fraction does not contain many enriched lipids, but functions to remove residual methanol/chloroform that will prevent separation of the last butanol extraction. The last extraction uses 100 μl butanol and 400 μl MTBE both of which are water saturated before their addition to the remaining aqueous layer. The organic phases were combined and dried under vacuum to afford a lipid film that was stored at −80°C until targeted MS and MS/MS analysis.

For quantification of PA by ELISA, MEF or HeLa cells were treated as indicated and collected on ice and then lysed with 20 mM Hepes (Sigma, H4034) pH 7.4, 100 mM NaCl (Sigma, S7653), 1 mM EDTA (Sigma, E9884), 1% Triton X100 (Sigma, 9036‐19‐5) supplemented with Pierce protease inhibitor (ThermoFisher, A32963). Protein concentrations for each sample were then quantified using the Bio‐Rad Bradford assay. PA was then measured using the Human Phosphatidic Acid Antibody IgM ELISA Kit (Lifeome Bioloabs) following the manufacturers protocol and monitoring fluorescence on a Tecan F250Pro microplate reader (Tecan).

For quantification of PA by fluorometric biochemical assay, MEF or HeLa cells were treated as indicated and collected on ice and then centrifuged and washed with cold PBS three times. Samples were then sonicated using the Misonix S‐4000 sonicator then processed and PA was measured according the manufacturer's protocol for the Total Phosphatidic Assay Kit (CellBio Labs).

### Immunoblotting and antibodies

Whole cells were lysed at room temperature in HEPES lysis buffer (20 mM Hepes pH 7.4, 100 mM NaCl, 1 mM EDTA, 1% Triton X100) supplemented with 1× Pierce protease inhibitor (ThermoFisher). Total protein concentrations of lysates were then normalized using the Bio‐Rad protein assay and lysates were combined with 1× Laemmli buffer supplemented with 100 mM DTT and boiled for 5 min. Samples (100 μg protein) were then separated using 10 or 12% SDS–PAGE gels and transferred to nitrocellulose membranes (Bio‐Rad). Membranes were then blocked with 5% milk in tris‐buffered saline (TBS) and then incubated overnight at 4°C with the indicated primary antibody. The next day, membranes were washed in TBS supplemented with Tween, incubated with the species appropriate secondary antibody conjugated to IR‐Dye (LICOR Biosciences) and then imaged using an Odyssey Infrared Imaging System (LICOR Biosciences). Quantification was then carried out using the LICOR Imaging Studio software.

Primary antibodies were acquired from commercial sources and used in the indicated dilutions in antibody buffer (50 mM Tris [pH 7.5], 150 mM NaCl supplemented with 5% BSA (w/v) and 0.1% NaN_3_ (w/v)): TIM17A (Thermo Scientific, PA5‐21925; 1:1,000), PRELID1 [aa27‐54] (LS Bio, LC‐C158729; 1:1,000), YME1L (Proteintech, 11510‐1‐AP; 1:1,000), ATF4 (Cell Signaling, 11815; 1:500), Tubulin [B‐5‐1‐2] (Sigma, T6074; 1:5,000), TIM23 (BD Transduction Labs, 611222; 1:1,000), HSP60 [LK1] (Thermo Scientific, MA1‐35434; 1:1,000), PERK (Cell Signaling, 3192S; 1:1,000), HA [Clone: 16B12] (Biolegend, 901501; 1:1,000), GFP (B2; Santa Crux, sc9996; 1:1,000), OPA1 (BD Biosciences, 612606; 1:2,000), Phospho‐DRP1 (Ser616) (ThermoFisher, PA5‐64821; 1:1,000), Phospho‐DNM1L (Ser637; ThermoFisher, PA5; 101038; 1:1,000), DNM1L (ThermoFisher, MA5‐26255; 1:1,000), p70 S6 Kinase p(Ser389; Cell Signaling, 9206S; 1:1,000), p70 S6 Kinase (Cell Signaling, 9202S; 1:1,000).

### Quantitative polymerase chain reaction (qPCR)

The relative mRNA expression of target genes was measured using quantitative RT‐PCR. Cells were treated as indicated and then washed with phosphate buffered saline (PBS; Gibco). RNA was extracted using Quick‐RNA MiniPrepKit (Zymo Research) according to the manufacturers protocol. RNA (500 ng) was then converted to cDNA using the QuantiTect Reverse Transcription Kit (Qiagen). qPCRs were prepared using Power SYBR Green PCR Master Mix (Applied Biosystems), and primers (below) were obtained from Integrated DNA Technologies. Amplification reactions were run in an ABI 7900HT Fast Real Time PCR machine with an initial melting period of 95°C for 5 min and then 45 cycles of 10 s at 95°C, 30 s at 60°C.

### Primers used in this study

*Human ASNS*: forward: GCAGCTGAAAGAAGCCCAAG; reverse: AGCCTGAATGCCTTCCTCAG.

*Human CHOP/DDIT3*: forward: ACCAAGGGAGAACCAGGAAACG; reverse: TCACCATTCGGTCAATCAGAGC.

*Human HSPA5/BIP*: forward: GCCTGTATTTCTAGACCTGCC; reverse: TTCATCTTGCCAGCCAGTTG.

*Human RIBOP*: forward: CGT CGC CTC CTA CCT GCT; reverse: CCA TTC AGC TCA CTG ATA ACC TTG.

### Membrane depolarization

Cells were seeded at a density of 85,000 cells/well of a six‐well plate and treated with 500 nM Tg for 3 h prior to collection. CCCP (10 μM) was added 50 min before collection, followed by 200 nM TMRE (Thermofisher) 20 min before collection. Samples were collected using TrypLE Express and cell culture media. Following a brief centrifugation, cell pellets were washed in DPBS (Gibco) and resuspended in DPBS supplemented with 5% BSA. Fluorescence intensity of TMRE for 20,000 cells/condition was recorded on the PE channel of a BD Biosciences LSR II analytical flow cytometer. Data are presented as geometric mean of the fluorescence intensity from three experiments normalized to vehicle‐treated cells.

### Mitochondrial isolation

Whole cells were collected on ice from at least 3 × 10 cm plates then pelleted. Cells were mixed with mitochondrial lysis buffer (220 mM sorbitol; 70 mM sucrose; 50 mM MOPS pH 7.4; 5 mM EGTA) supplemented with 1× Pierce protease inhibitor (ThermoFisher) and lysed by passing through a 21 guage needle 10–20 times. Lysed cells were spun down at 1,000 × *g* for 10 min to pellet the nuclei and unlysed cells. The supernatant was transferred into a new tube and spun down at 9,500 × *g* for 10 min to pellet the mitochondria. The supernatant was saved as a cytosolic control. For immunoblotting, mitochondrial pellets were lysed on ice for 10 min in mitochondrial wash buffer (220 mM sorbitol; 70 mM sucrose; 50 mM MOPS pH 7.4) supplemented with 1% Triton and protein concentration was determined using the Bio‐rad protein assay and prepared as described above.

### Statistical analysis

Statistics were calculated in PRISM 9 (GraphPad, San Diego, CA). Data are presented as mean ± SEM and were analyzed by two‐way ANOVA with Tukey's multiple correction test, one‐way ANOVA, or the appropriate Student's *t*‐tests, as indicated in the accompanying figure legends. Indications of nonsignificant interactions from ANOVA were generally omitted for clarity.

## Author contributions

**Valerie Perea:** Conceptualization; formal analysis; investigation; visualization; writing – original draft; writing – review and editing. **Christian Cole:** Conceptualization; formal analysis; investigation; writing – review and editing. **Justine Lebeau:** Conceptualization; formal analysis; investigation; writing – review and editing. **Vivian Dolina:** Conceptualization; formal analysis; investigation; writing – review and editing. **Kelsey R Baron:** Formal analysis; investigation; writing – review and editing. **Aparajita Madhavan:** Formal analysis; investigation; writing – review and editing. **Jeffery W Kelly:** Supervision. **Danielle A Grotjahn:** Supervision; visualization; writing – original draft; project administration; writing – review and editing. **R Luke Wiseman:** Conceptualization; formal analysis; supervision; investigation; visualization; writing – original draft; project administration; writing – review and editing.

## Disclosure and competing interests statement

The authors declare that they have no conflict of interest.

## Supporting information



Expanded View Figures PDF

Source Data for Expanded View

PDF+

Source Data for Figure 3

Source Data for Figure 4

## Data Availability

This study includes no data deposited in external repositories.
