# Supplementary material for: PERK signaling promotes mitochondrial elongation by remodeling membrane phosphatidic acid
Source: EMBO J. 2023 Jun 12;42(15):e113908. doi: 10.15252/embj.2023113908 (PMC10390871; doi:10.15252/embj.2023113908)
Supplement: Supplementary file 1 — Expanded View Figures PDF [file EMBJ-42-e113908-s002.pdf]

Expanded View Figures

Figure EV1. Supplement to Fig 1. ER stress-induced mitochondrial elongation is impaired in cells expressing a hypomorphic PERK variant.

A Representative images of fragmented, tubular, and elongated mitochondria in MEF^{mtGFP} cells.
B Representative images of fragmented, tubular, and elongated mitochondria in HeLa cells expressing ^{mt}GFP.
C Total cells counted for each treatment condition for mitochondrial morphology qualifications in Fig 1B and C.
D Mitochondrial polarization, measured by TMRE fluorescence, in *Perk*$^{+/+}$ MEFs and *Perk*$^{-/-}$ MEFs treated 30 min with CCCP (10 µM). Error bars show SEM for $n = 3$ biological replicates. ***$P < 0.005$ for one-way ANOVA.
E Representative images of *Perk*$^{+/+}$ MEFs, *Perk*$^{-/-}$ MEFs, or *Perk*$^{-/-}$ MEFs transfected with wild-type $PERK^{WT}$ or the PSP-associated PERK allele ($PERK^{PSP}$) expressing ^{mt}GFP treated for 3 h with cycloheximide (CHX; 50 µg/ml). The inset shows twofold magnification of the image centered on the asterisk. Scale bars, 5 µm.
F Immunoblot of lysates prepared from *Perk*$^{+/+}$ MEFs, *Perk*$^{-/-}$ MEFs, or *Perk*$^{-/-}$ MEFs transfected with wild-type $PERK^{WT}$ or the PSP-associated PERK allele ($PERK^{PSP}$) treated for 6 h with thapsigargin (Tg; 500 nM) or cycloheximide (CHX; 50 µg/ml).

Source data are available online for this figure.

© 2023 The Authors

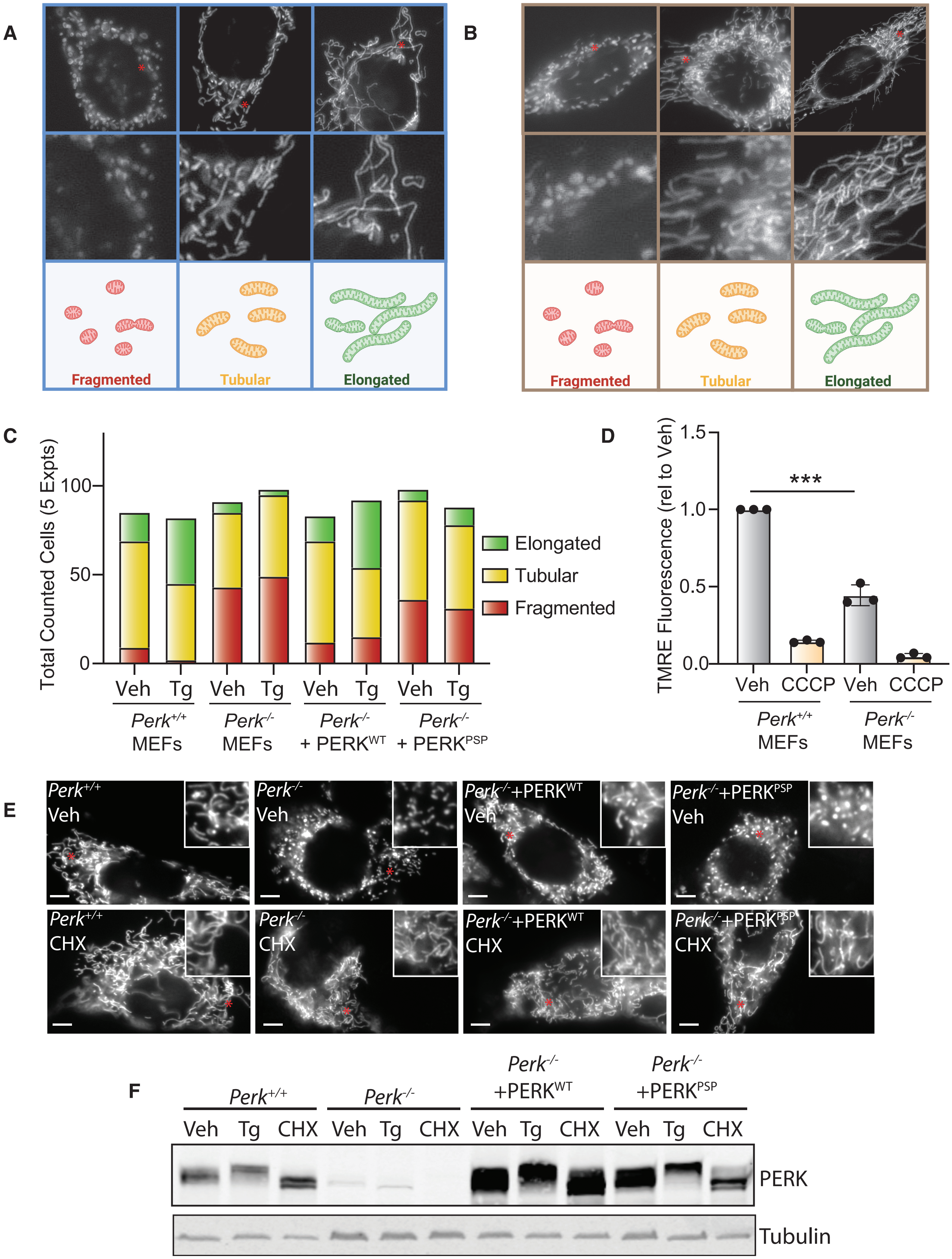

Figure EV1.

 © 2023 The Authors

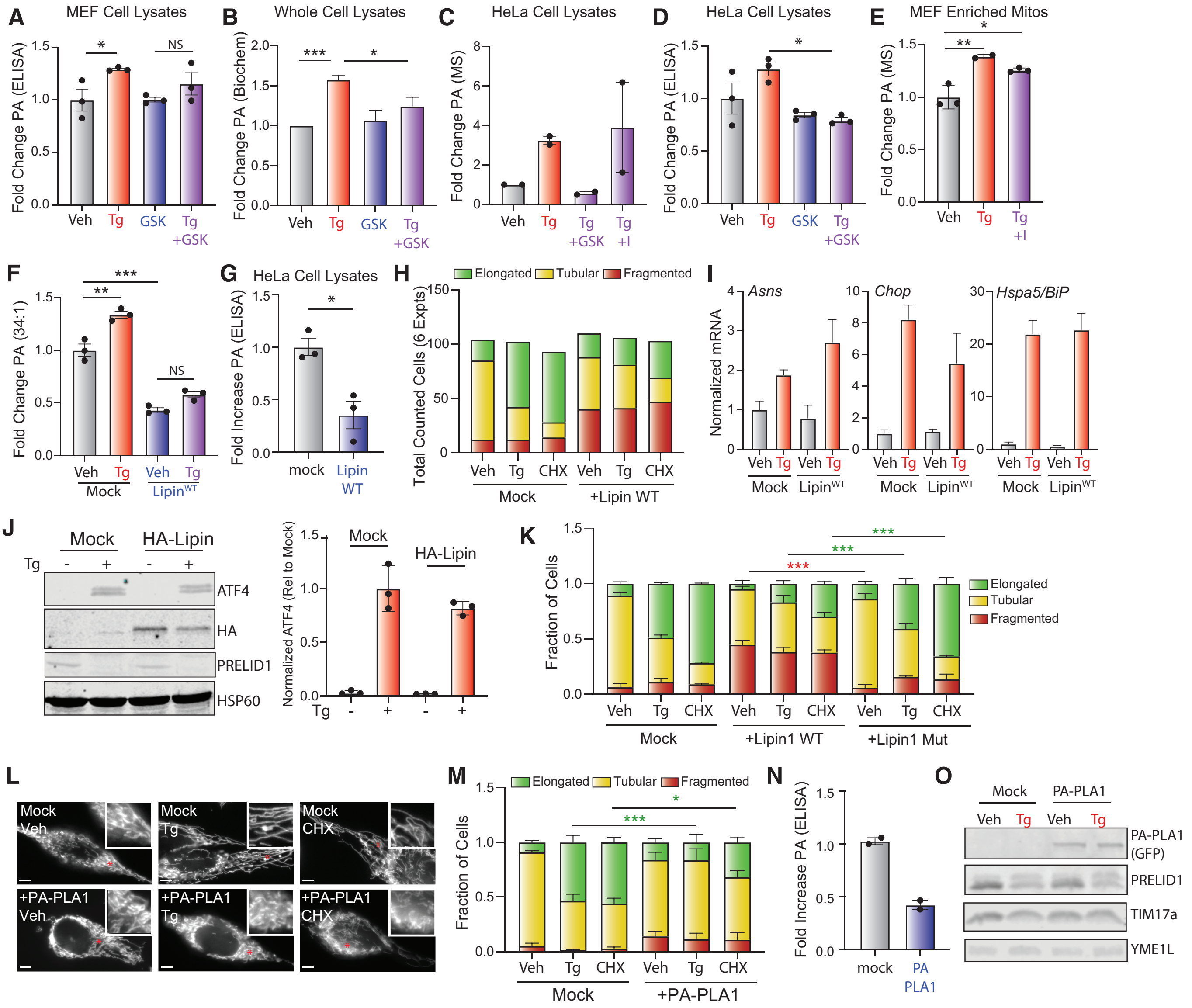

A MEF Cell Lysates
Fold Change PA (ELISA)
Veh Tg GSK Tg +GSK
B Whole Cell Lysates
Fold Change PA (Biochem)
C HeLa Cell Lysates
Fold Change PA (MS)
Veh Tg Tg +GSK Tg +I
D HeLa Cell Lysates
E MEF Enriched Mitos
Fold Change PA (MS)
F
Fold Change PA (34:1)
Mock Lipin^WT
G HeLa Cell Lysates
Fold Increase PA (ELISA)
mock Lipin WT
H
Elongated Tubular Fragmented
Total Counted Cells (6 Expts)
Veh Tg CHX Veh Tg CHX
Mock +Lipin WT
I
Asns Chop Hspa5/BiP
Normalized mRNA
J
Mock HA-Lipin
ATF4 HA PRELID1 HSP60
Normalized ATF4 (Rel to Mock)
K
Fraction of Cells
Mock +Lipin1 WT +Lipin1 Mut
L
Mock Veh
Mock Tg
Mock CHX
+PA-PLA1 Veh
+PA-PLA1 Tg
+PA-PLA1 CHX
M
Mock +PA-PLA1
N
Fold Increase PA (ELISA)
mock PA PLA1
O
Mock PA-PLA1
PA-PLA1 (GFP)
PRELID1
TIM17a
YME1L

Figure EV2.

Figure EV2. Supplement to Fig 2. Overexpression of PA lipases inhibits ER stress-induced mitochondrial elongation.

A, B Relative phosphatidic acid (PA), measured by ELISA (A) or biochemical assay (B) in whole cell extracts isolated from MEF cells treated for 3 h with vehicle, thapsigargin (Tg, 500 nM), and/or GSK2656157 (1 µM). Error bars show SEM for $n = 3$ biological replicates $*P < 0.05$ for unpaired t-test (A) or $*P < 0.05$ $***P < 0.005$ for paired t-test (B).

C Relative PA, measured by untargeted mass spectrometry, in lysates prepared from HeLa cells treated for 3 h with vehicle, thapsigargin (Tg, 500 nM), GSK2656157 (GSK; 1 µM), or ISRIB (I; 200 nM), as indicated. Error bars show SEM for $n = 2$ biological replicates.

D Relative PA, measured by ELISA, in lysates prepared from HeLa cells treated for 3 h with vehicle, thapsigargin (Tg, 500 nM), and/or GSK2656157 (GSK; 1 µM). Error bars show SEM for $n = 3$ biological replicates and $*P < 0.05$ for one-way ANOVA.

E Relative PA levels, measured by untargeted mass spectrometry, in mitochondrial enriched fractions from MEF cells treated for 3 h with vehicle, thapsigargin (Tg; 500 nM), and/or ISRIB (I; 0.2 µM). Error bars show SEM for $n = 2$–3 biological replicates. $**P < 0.01$, $***P < 0.005$ for one-way ANOVA.

F Normalized relative abundance of PA 34:1, as measured by targeted MS, in lysates of HeLa cells expressing mock or Lipin^{WT} and treated for 3 h with vehicle or thapsigargin (Tg; 500 nM), as indicated. Error bars show SEM for $n = 3$ biological replicates. $**P < 0.01$, $***P < 0.005$ for one-way ANOVA.

G Relative PA, measured by ELISA, in lysates of HeLa cells expressing mock or Lipin^{WT} and treated for 3 h with vehicle or thapsigargin (Tg; 500 nM), as indicated. Error bars show SEM for $n = 3$ biological replicates. $*P < 0.05$ for unpaired t-test.

H Total counted cells for qualitative analysis of mitochondrial morphology for Fig 2D and E.

I Expression, measured by qPCR, of *Asns*, *Chop*, and *Hspa5/BiP* in HeLa cells expressing mock or Lipin^{WT} treated for 3 h with vehicle or thapsigargin (Tg, 500 nM). Error bars show ±95% confidence interval for $n = 3$ technical replicates.

J Immunoblot lysates prepared form HeLa cells expressing mock or Lipin^{WT} treated for 3 h with vehicle or thapsigargin (Tg; 500 nM). Note that the Lipin construct is HA tagged allowing detection with the HA antibody. Quantification of ATF4 from three different experiments is also shown.

K Quantification of fragmented (red), tubular (yellow), or elongated (green) mitochondria from HeLa cells transfected with either mock, Lipin^{WT} and a catalytically inactive lipin1 mutant (Lipin^{mut}) treated for 3 h with vehicle, thapsigargin (Tg; 500 nM) or cycloheximide (CHX; 50 µg/ml). Error bars show SEM for $n = 3$ independent experiments. P-value reflects comparisons of elongated (green) mitochondria populations for the indicated conditions. $***P < 0.005$ for two-way ANOVA.

L Representative images of HeLa cells expressing ^{mt}GFP transfected with mock or GFP-tagged PA-PLA1 then treated for 3 h with thapsigargin (Tg; 500 nM) or cycloheximide (CHX; 50 µg/ml). The inset shows 2-fold magnification of the image centered on the asterisk. Scale bars, 5 µm. Note that the presence of GFP on PA-PLA1 did not influence our ability to monitor mitochondrial morphology in these cells.

M Quantification of fragmented (red), tubular (yellow), or elongated (green) mitochondria from the images shown in (L). Error bars show SEM for $n = 3$ independent experiments. $*P < 0.05$, $***P < 0.005$ for two-way ANOVA (green indicates comparisons between elongated mitochondria fractions).

N Relative PA, measured by ELISA, in lysates of HeLa cells expressing mock or PA-PLA1. Error bars show SEM for $n = 2$ biological replicates.

O Immunoblot of lysates prepared from HeLa cells expressing mock or PA-PLA1 treated for 3 h with vehicle or thapsigargin (Tg; 500 nM).

Source data are available online for this figure.

© 2023 The Authors

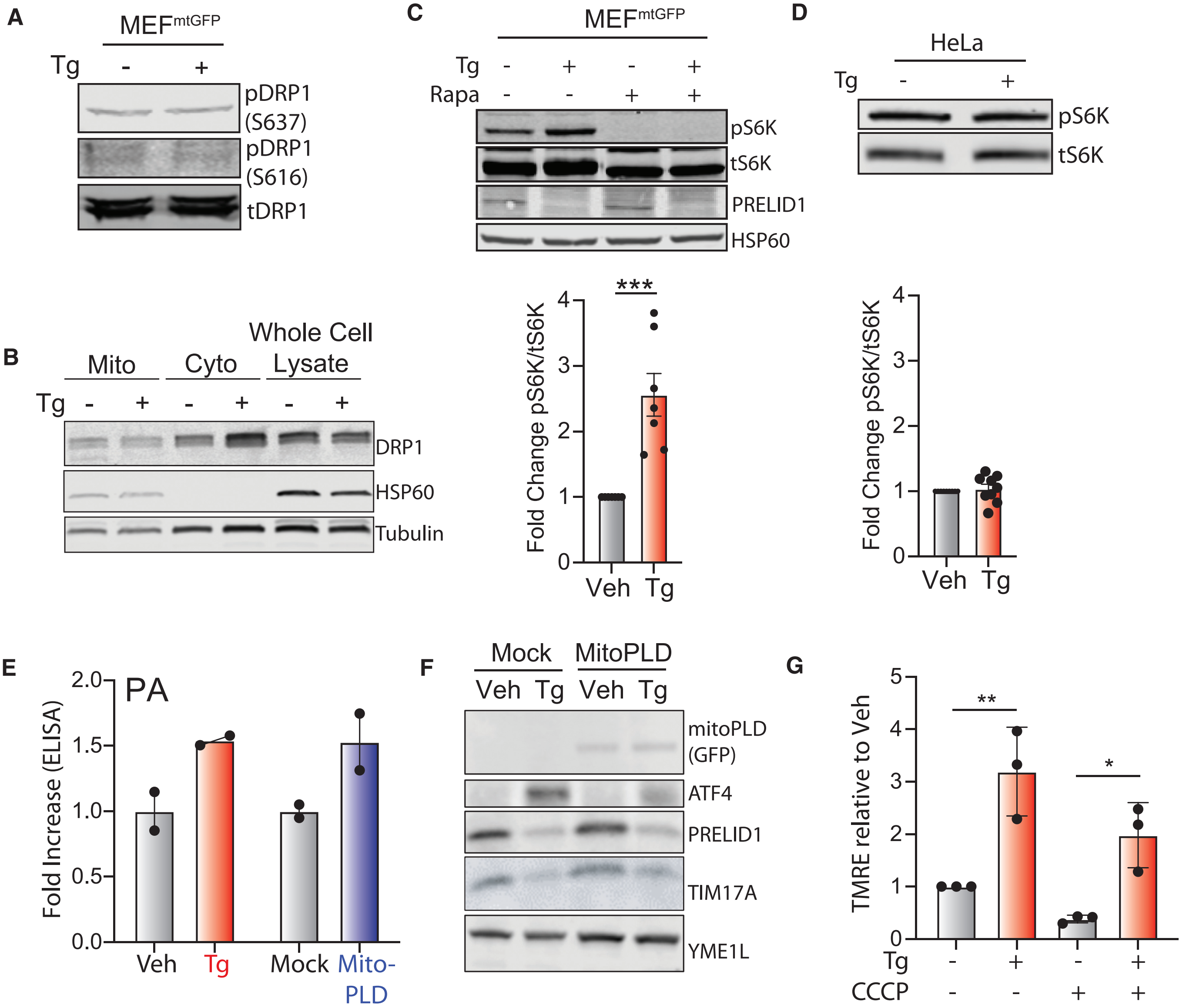

Figure EV3. Supplement to Fig 3. ER stress-induced mitochondrial elongation inhibits Ionomycin-induced mitochondrial fragmentation.

A Immunoblot of lysates prepared from MEF^{mtGFP} cells treated for 3 h with thapsigargin (Tg; 500 nM).

B Immunoblot of mitochondrial fractions, cytosolic fractions, or whole cell lysates from MEF^{mtGFP} cells treated for 3 h with thapsigargin (Tg; 500 nM).

C Immunoblot of lysates prepared from MEF^{mtGFP} cells treated for 3 h with thapsigargin (Tg; 500 nM) and/or rapamycin (Rapa; 10 μM). Quantification of pS6K normalized to tS6K is shown. Error bars show SEM for $n = 7$ independent experiments. ***$P < 0.005$ for paired t-test.

D Immunoblot of lysates prepared from HeLa cells treated for 3 h with vehicle or thapsigargin (Tg; 500 nM). Quantification of pS6K normalized to tS6K is shown. Error bars show SEM for $n = 9$ independent experiments.

E Phosphatidic acid (PA), measured by ELISA, in HeLa cells treated with thapsigargin (Tg; 500 nM, 3 h) or expressing mock or mitoPLD. Error bars show SEM for $n = 2$ biological replicates. Individual replicates are shown.

F Immunoblot of lysates prepared from HeLa cells transfected with mock or mitoPLD^{GFP} and treated for 3 h with vehicle or thapsigargin (Tg; 500 nM). Note mitoPLD^{GFP} is tagged with GFP allowing detection of this protein with the GFP antibody.

G Mitochondrial polarization, measured by TMRE fluorescence, in MEF cells pre-treated for 3 h with thapisgargin (Tg; 500 nM) then challenged for 30 min with CCCP (10 μM). Error bars show SEM for $n = 3$ biological replicates. *$P < 0.05$, **$P < 0.01$, ***$P < 0.005$ for one-way ANOVA.

Source data are available online for this figure.

© 2023 The Authors

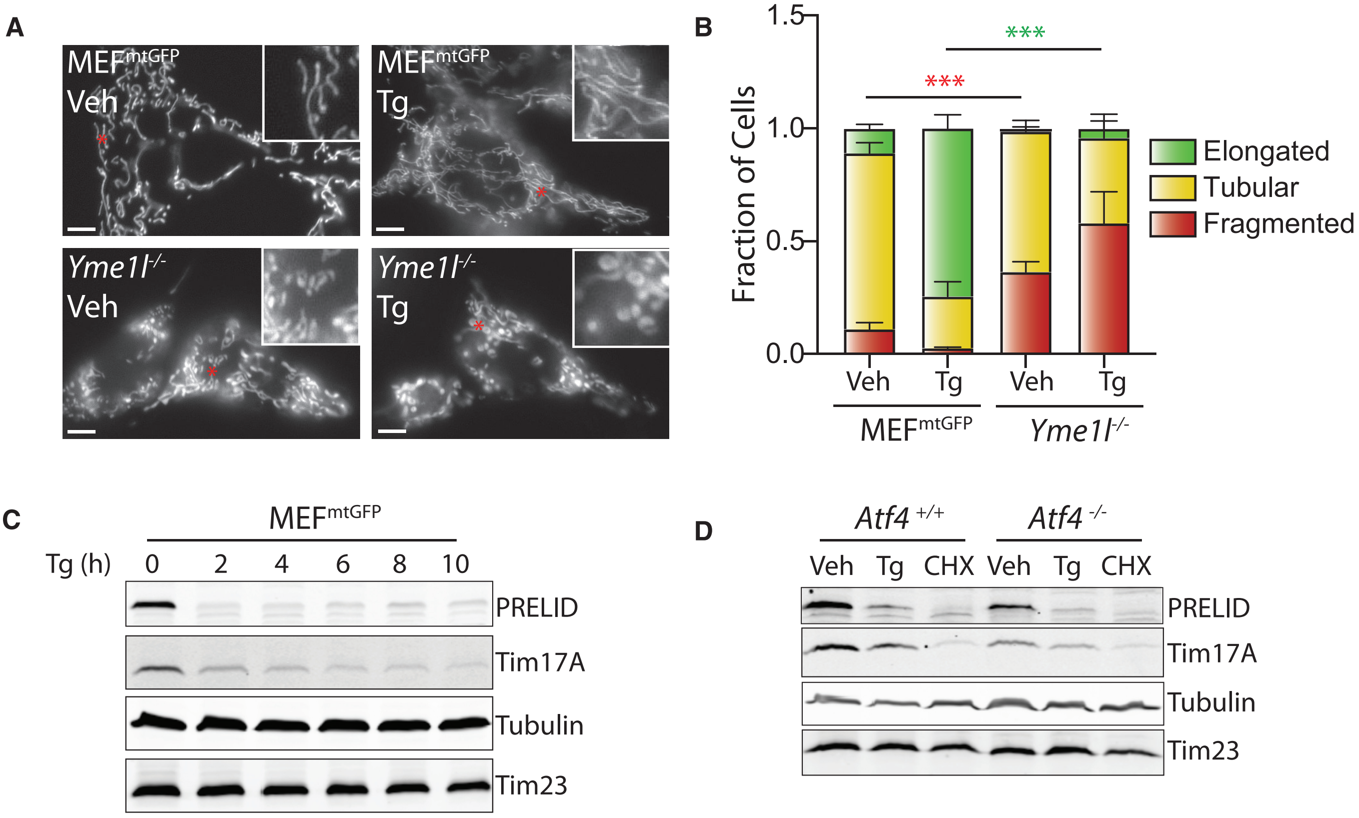

Figure EV4. Supplement to Fig 4. ER stress reduces PRELID1 through a YME1L-dependent mechanism downstream of PERK-dependent translational attenuation.

A Representative images of MEF^{mtGFP} cells and *Yme1l*-deficient MEF^{mtGFP} cells treated for 6 h with thapsigargin (Tg; 500 nM). The inset shows twofold magnification of the image centered on the asterisk. Scale bars, 5 μm.

B Quantification of fragmented (red), tubular (yellow), or elongated (green) mitochondria from the images shown in (A). Error bars show SEM for $n = 7$ independent experiments. *P*-value reflects comparisons of elongated (green) or fragmented (red) mitochondria populations for the indicated conditions. ***$P < 0.005$ for two-way ANOVA (red indicates comparison between fragmented mitochondria fractions; green indicates comparisons between elongated mitochondria fractions).

C Immunoblot of lysates prepared from MEF^{mtGFP} cells treated with thapsigargin (Tg; 500 nM) for the indicated time.

D Immunoblot of lysates prepared from $Atf4^{+/+}$ and $Atf4^{-/-}$ MEFs treated with thapsigargin (Tg; 500 nM) or CHX (50 μg/ml) for 3 h.

Source data are available online for this figure.

 © 2023 The Authors

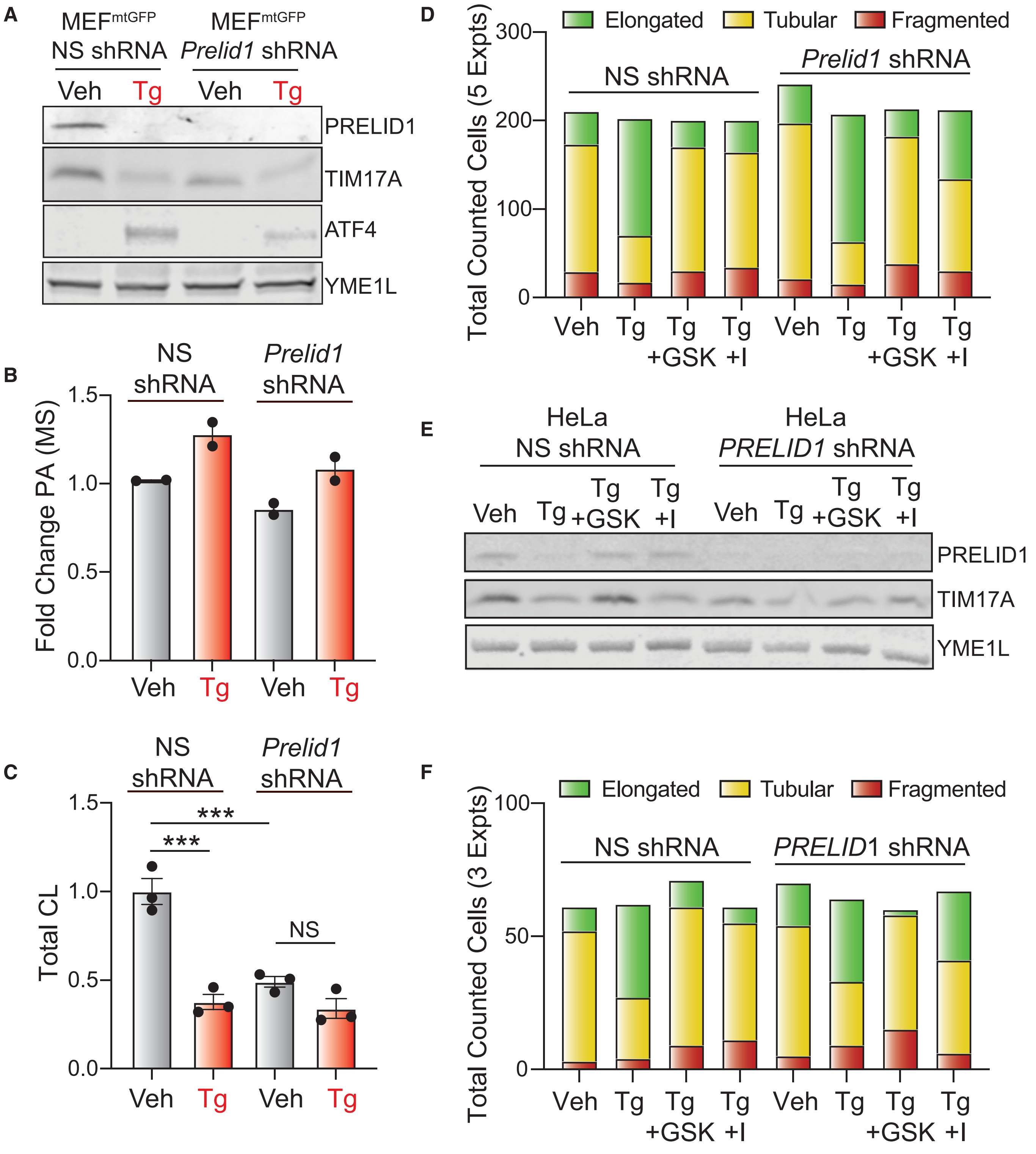
A
MEF mtGFP NS shRNA
MEF mtGFP Prelid1 shRNA
Veh Tg Veh Tg
PRELID1
TIM17A
ATF4
YME1L
B
NS shRNA
Prelid1 shRNA
Fold Change PA (MS)
1.5
1.0
0.5
0.0
Veh Tg Veh Tg
C
NS shRNA
Prelid1 shRNA
Total CL
***
***
NS
1.5
1.0
0.5
0.0
Veh Tg Veh Tg
D
Elongated Tubular Fragmented
Total Counted Cells (5 Expts)
NS shRNA
Prelid1 shRNA
300
200
100
0
Veh Tg Tg +GSK Tg +I Veh Tg Tg +GSK Tg +I
E
HeLa NS shRNA
HeLa PRELID1 shRNA
Veh Tg Tg +GSK Tg +I Veh Tg Tg +GSK Tg +I
PRELID1
TIM17A
YME1L
F
Elongated Tubular Fragmented
Total Counted Cells (3 Expts)
NS shRNA
PRELID1 shRNA
100
50
0
Veh Tg Tg +GSK Tg +I Veh Tg Tg +GSK Tg +I

Figure EV5.

Figure EV5. Supplement to Fig 5. Reductions in PRELID1 contribute to ER stress-induced mitochondrial elongation.

A Immunoblot of lysates from MEF^{mtGFP} cells expressing non-silencing (NS) or *Prelid1* shRNA treated for 3 h with vehicle or thapsigargin (Tg; 500 nM).

B Relative amounts of phosphatidic acid (PA), measured by untargeted mass spectrometry, in lysates prepared from MEF^{mtGFP} cells expressing non-silencing (NS) or *Prelid1* shRNA treated for 3 h with vehicle or thapsigargin (Tg; 500 nM). Error bars show SEM for $n = 2$ independent replicates.

C Normalized relative abundance of total cardiolipin (CL) species measured by targeted mass spectrometry in isolated mitochondria prepared from MEF^{mtGFP} cells expressing non-silencing (NS) or *Prelid1* shRNA treated for 3 h with vehicle or thapsigargin (Tg; 500 nM). Error bars show SEM for $n = 3$ biological replicates. ***$P < 0.005$ for one-way ANOVA.

D Total counted cells for qualitative analysis of mitochondrial morphology in Fig 5A and B.

E Immunoblot of lysates prepared from HeLa cells expressing non-silencing (NS) or *PRELID1* shRNA and treated for 3 h with vehicle or thapsigargin (Tg, 500 nM), GSK2656157 (10 μM), and/or ISRIB (I; 2 μM), as indicated.

F Total counted cells for qualitative analysis of mitochondrial morphology for Fig 5C and D.

Source data are available online for this figure.

© 2023 The Authors
