# Supplementary material for: PERK signaling promotes mitochondrial elongation by remodeling membrane phosphatidic acid
Source: EMBO J. 2023 Jun 12;42(15):e113908. doi: 10.15252/embj.2023113908 (PMC10390871; doi:10.15252/embj.2023113908)

FIGURE EV2J Whole Gels (Last 4 lanes used for Figure EV2J)

ATF4

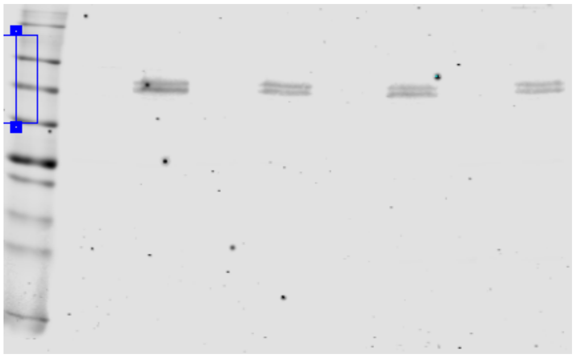

HA (Lipin)

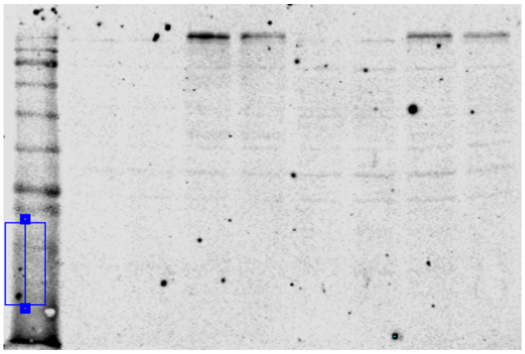

PRELID1

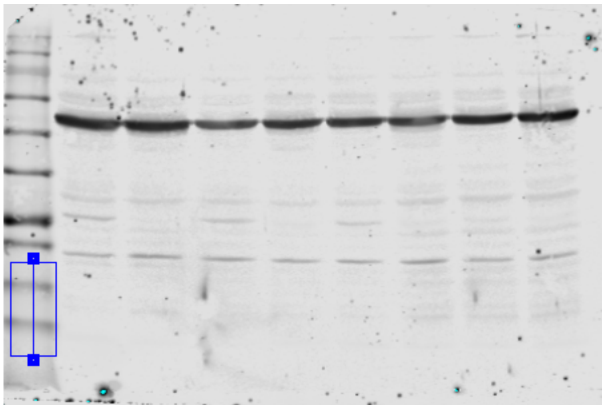

HSP60

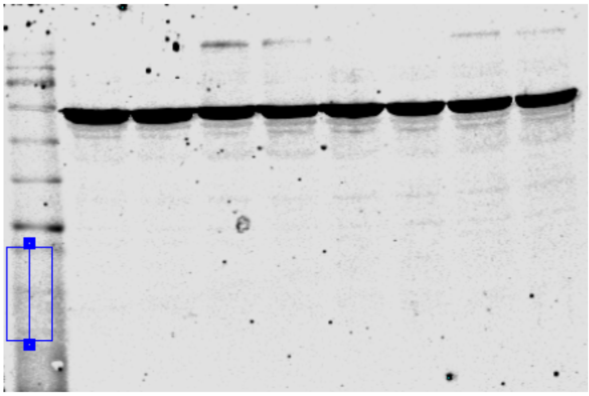

FIGURE EV2O Whole Gels

GFP (PA-PLA1)

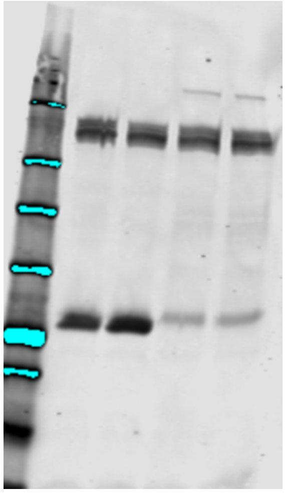

PRELID1

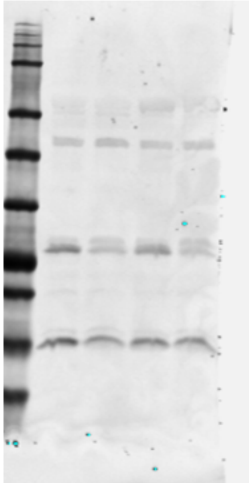

TIM17A (bottom) and YME1L (top)

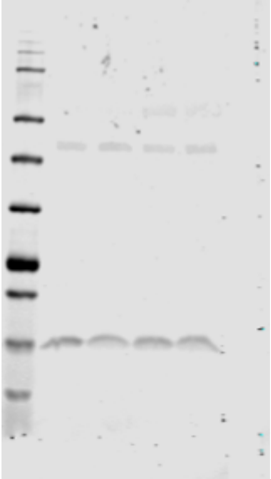

Supplement: Supplementary file 2 — Source Data for Expanded View [file EMBJ-42-e113908-s004.zip › Source Data (Whole Gels)/Figure EV2.pdf]
