# Supplementary material for: PERK signaling promotes mitochondrial elongation by remodeling membrane phosphatidic acid
Source: EMBO J. 2023 Jun 12;42(15):e113908. doi: 10.15252/embj.2023113908 (PMC10390871; doi:10.15252/embj.2023113908)

FIGURE EV3A Whole Gels

p-DRP1 (S637)

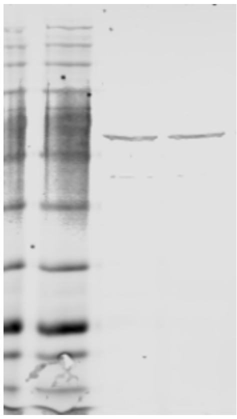

p-DRP1 (S616)

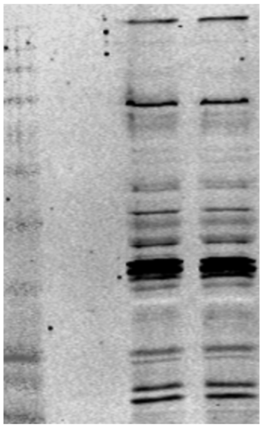

t-DRP1

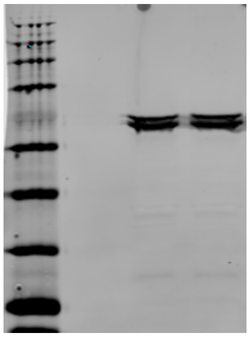

FIGURE EV3B Whole Gels

DRP1

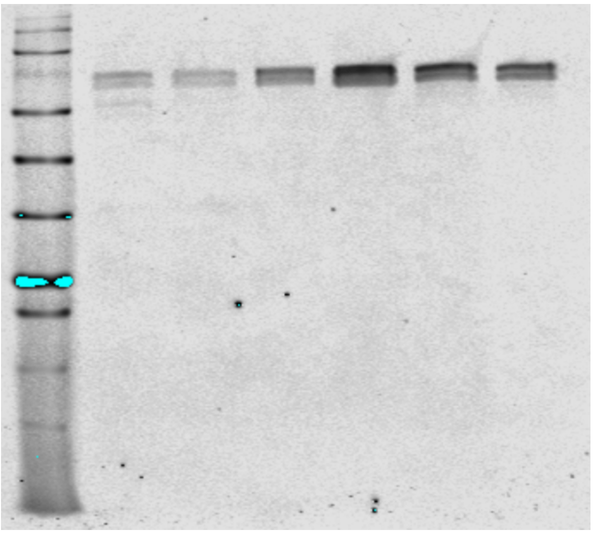

HSP60

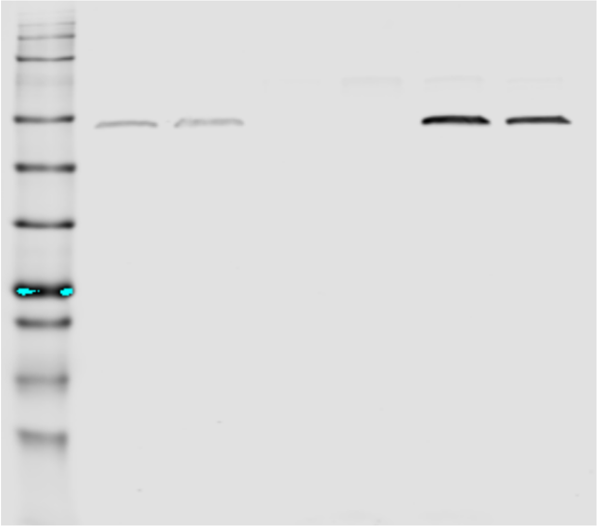

Tubulin

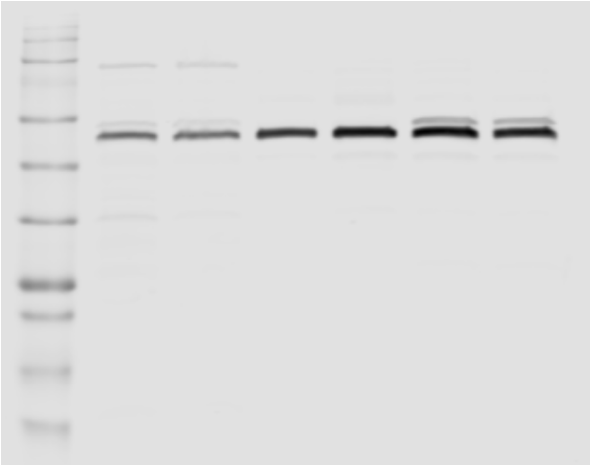

FIGURE EV3C Whole Gels

p-S6K

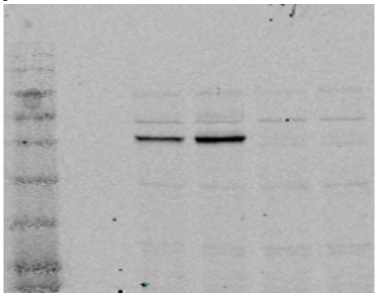

t-S6K

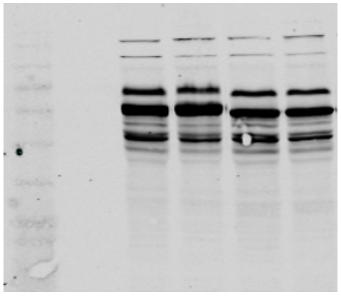

PRELID1

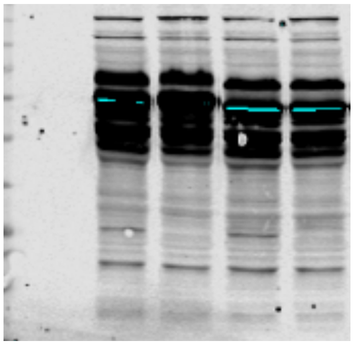

HSP60

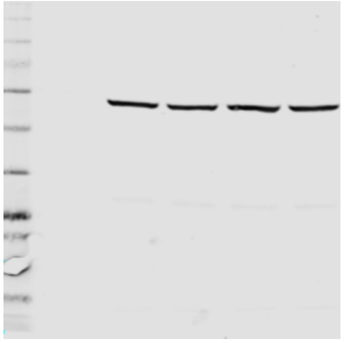

FIGURE EV3D Whole Gels

p-S6K

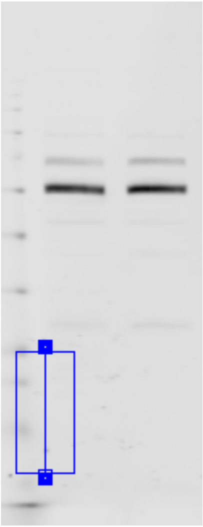

t-S6K

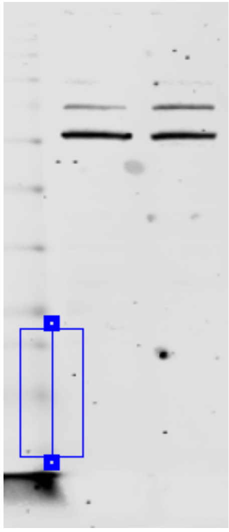

FIGURE EV3F Whole Gels

GFP

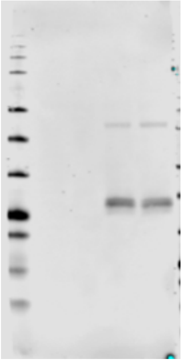

ATF4

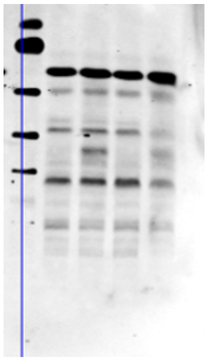

PRELID1

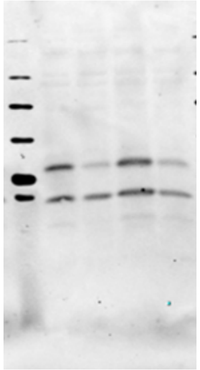

TIM17A

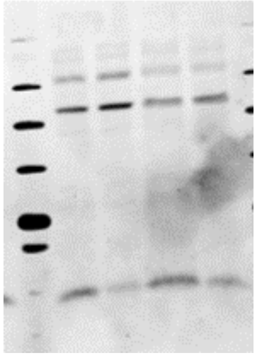

YME1L

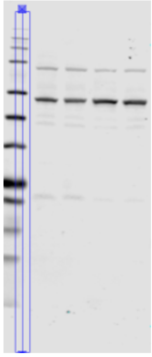

Supplement: Supplementary file 2 — Source Data for Expanded View [file EMBJ-42-e113908-s004.zip › Source Data (Whole Gels)/Figure EV3.pdf]
