# Supplementary figures and images for: PERK signaling promotes mitochondrial elongation by remodeling membrane phosphatidic acid
Source: EMBO J. 2023 Jun 12;42(15):e113908. doi: 10.15252/embj.2023113908 (PMC10390871; doi:10.15252/embj.2023113908)

FIGURE EV1F Whole Gels

PERK

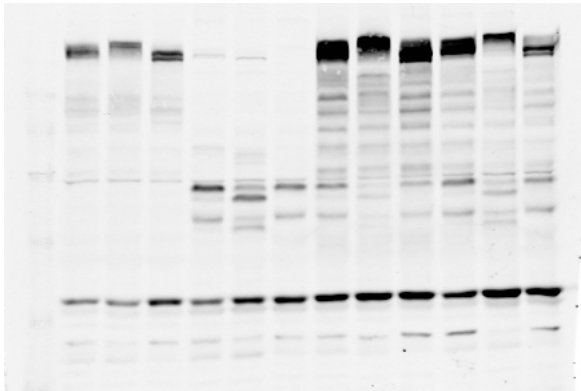

TUBULIN

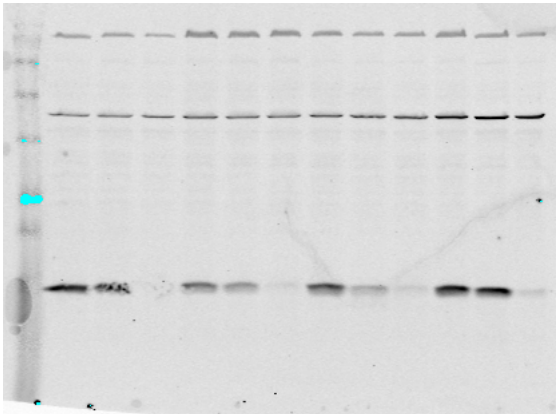

Supplement: Supplementary file 2 — Source Data for Expanded View [file EMBJ-42-e113908-s004.zip › Source Data (Whole Gels)/Figure EV1.pdf]

**FIGURE EV4C Whole Gels**

**PRELID1**

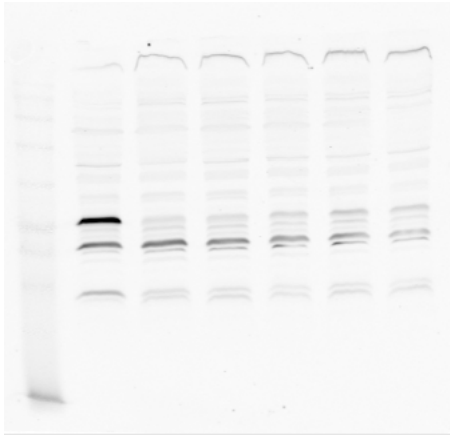

**TIM17A**

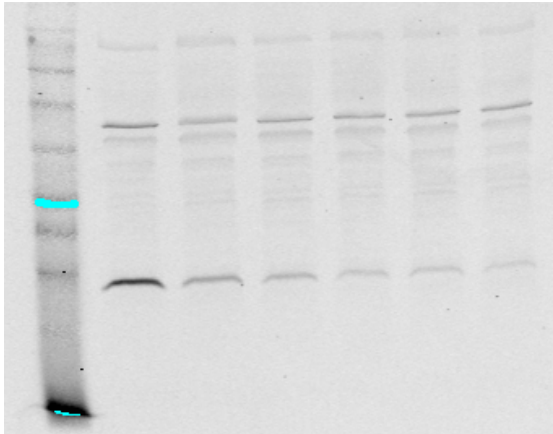

**TIM23**

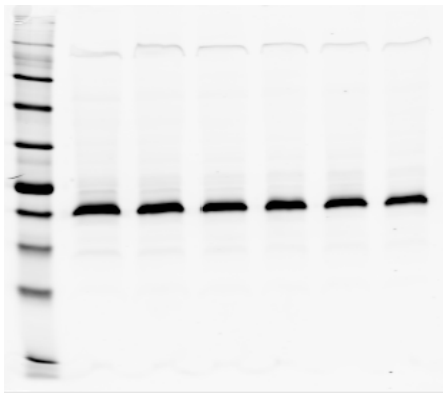

**TUBULIN**

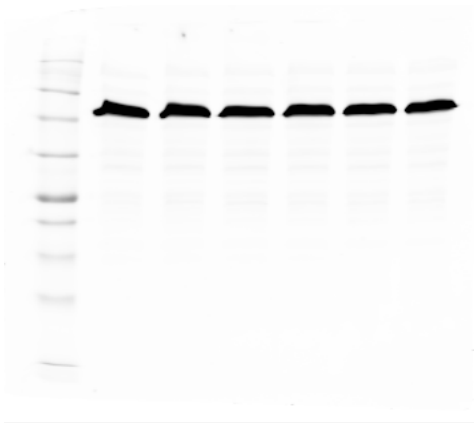

**FIGURE EV4D Whole Gels**

**PRELID1**

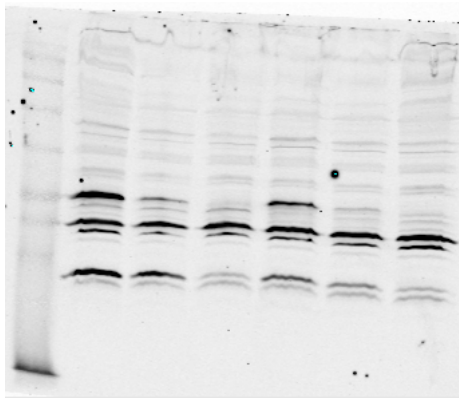

**TIM17A**

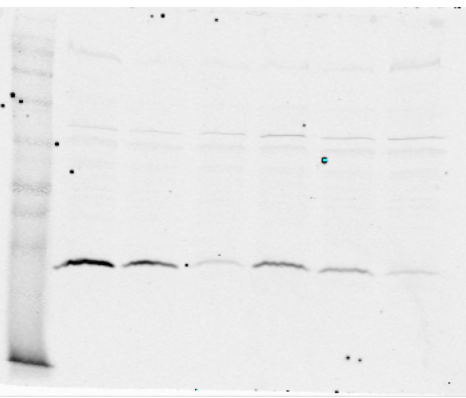

**TIM23**

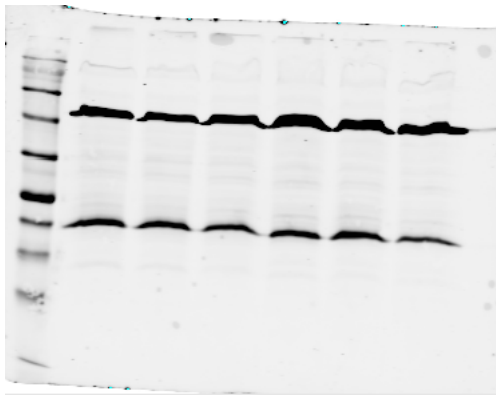

**TUBULIN**

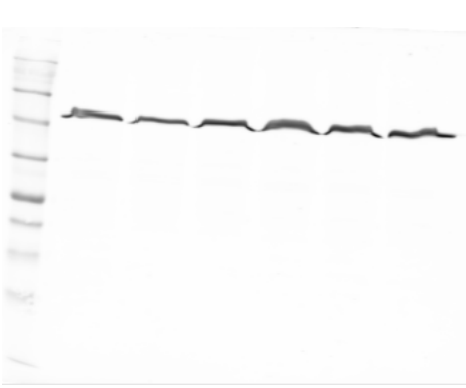

Supplement: Supplementary file 2 — Source Data for Expanded View [file EMBJ-42-e113908-s004.zip › Source Data (Whole Gels)/Figure EV4.pdf]

FIGURE EV5A Whole Gels

PRELID1

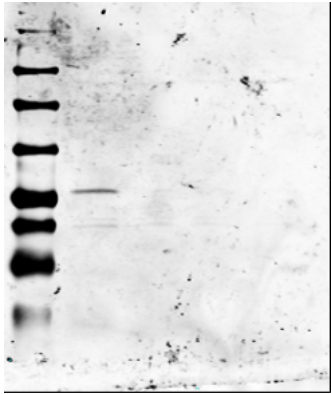

TIM17A

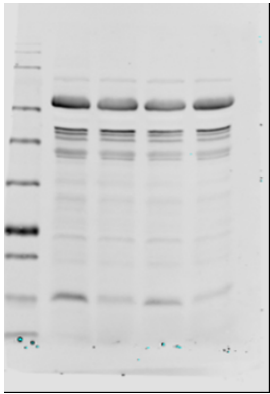

ATF4

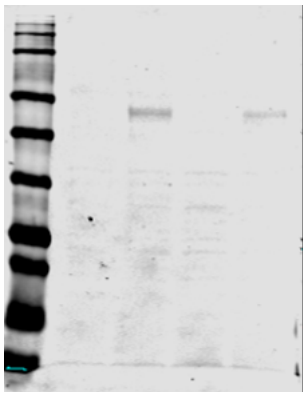

YME1L

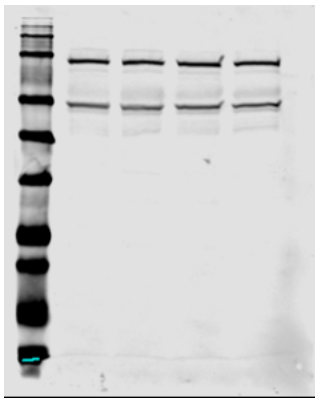

FIGURE EV5E Whole Gels

PRELID1

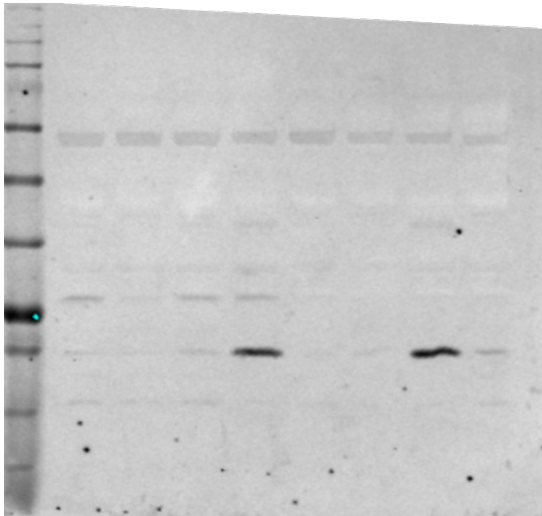

TIM17A

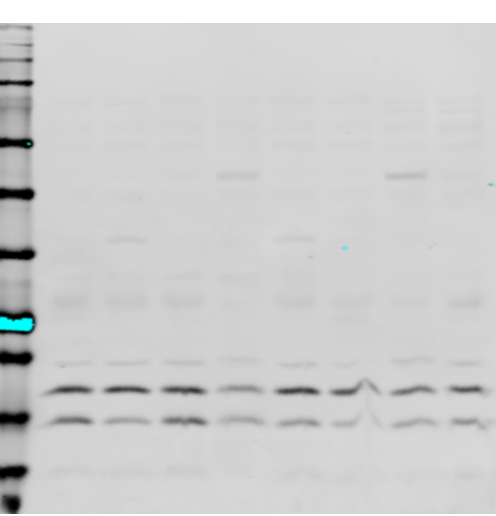

YME1L

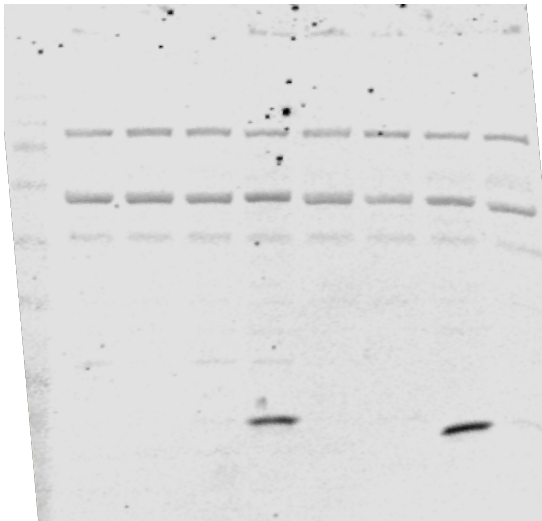

Supplement: Supplementary file 2 — Source Data for Expanded View [file EMBJ-42-e113908-s004.zip › Source Data (Whole Gels)/Figure EV5.pdf]

**Fig. 3C Whole Gels**

**YME1L**

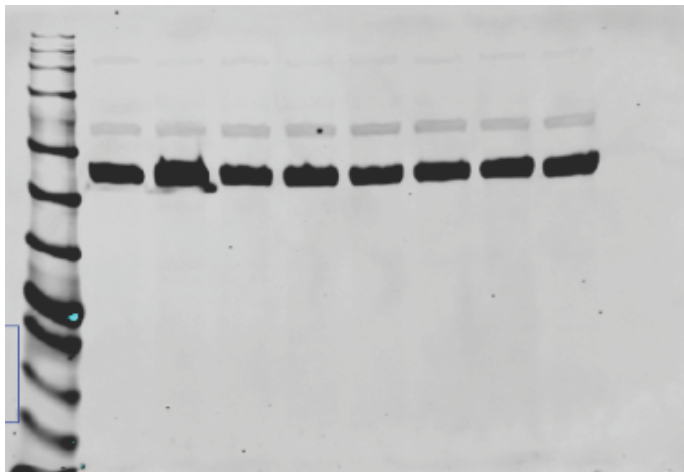

**OPA1 (top) and Tubulin (bottom)**

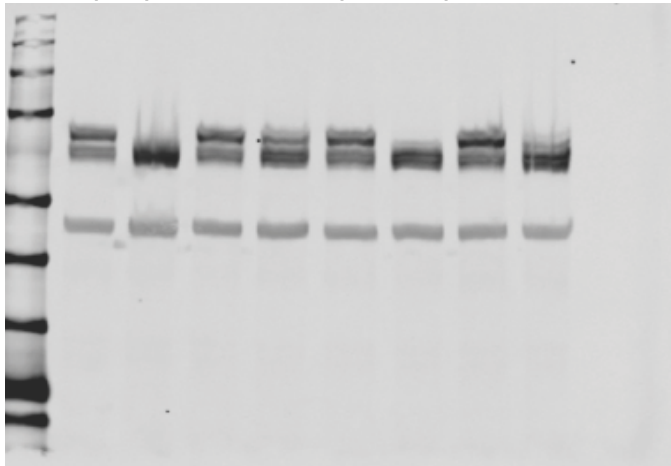

Supplement: Supplementary file 4 — Source Data for Figure 3 [file EMBJ-42-e113908-s003.zip › Figure 3.pdf]
