# Supplementary material for: PERK signaling promotes mitochondrial elongation by remodeling membrane phosphatidic acid
Source: EMBO J. 2023 Jun 12;42(15):e113908. doi: 10.15252/embj.2023113908 (PMC10390871; doi:10.15252/embj.2023113908)

FIGURE 4B Whole gels (last four lanes used for Figure 4B)

TIM17A

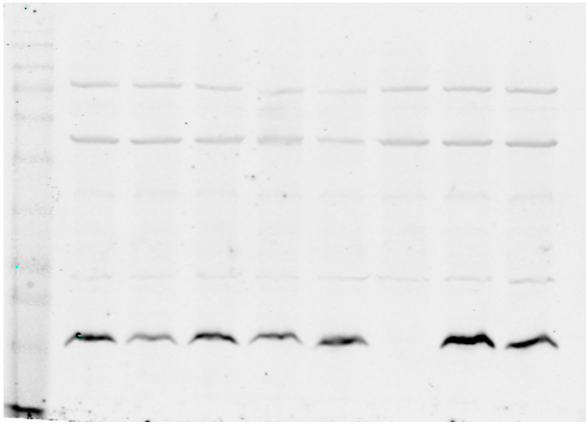

PRELID1

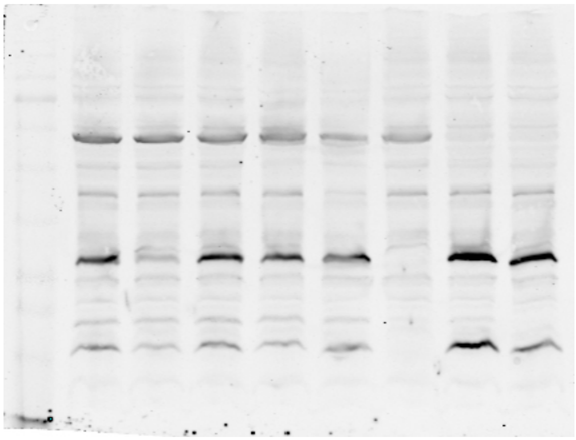

YME1L

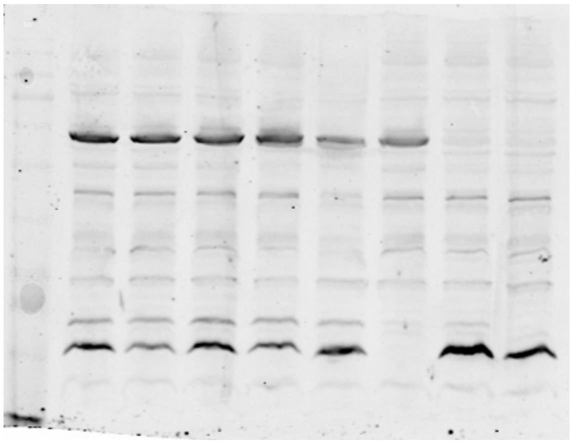

TIM23

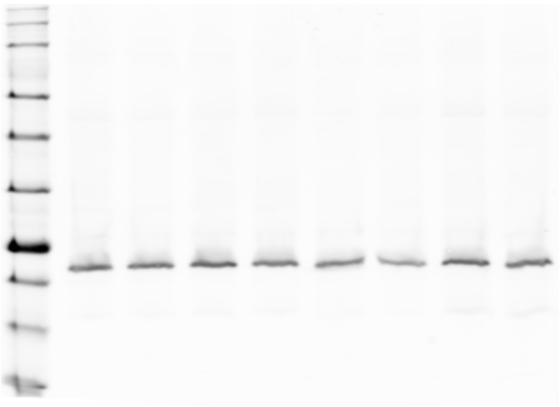

FIGURE 4C Whole Gels

PRELID1

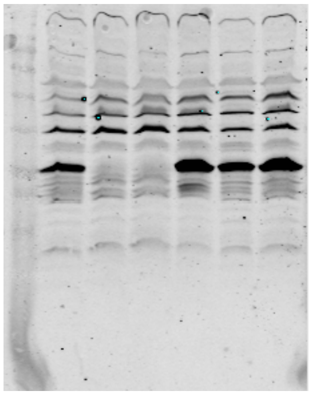

TIM17A

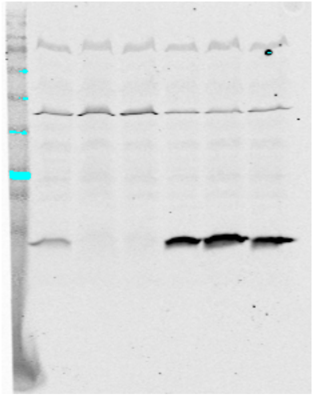

YME1L

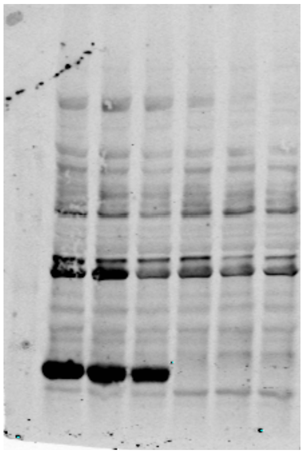

HSP60

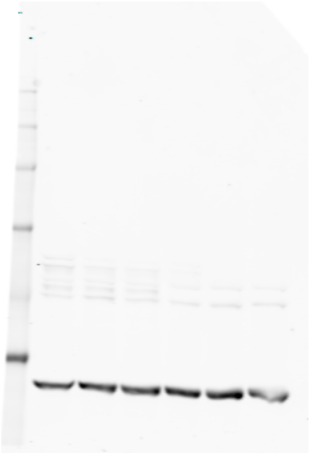

FIGURE 4D Whole Gels (first four lanes used for Figure 4D)

TIM17A

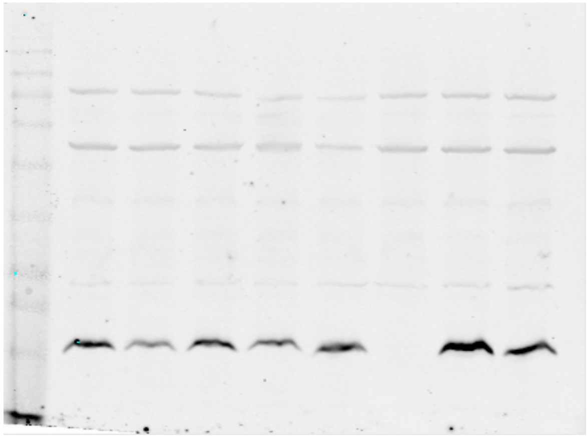

PRELID1

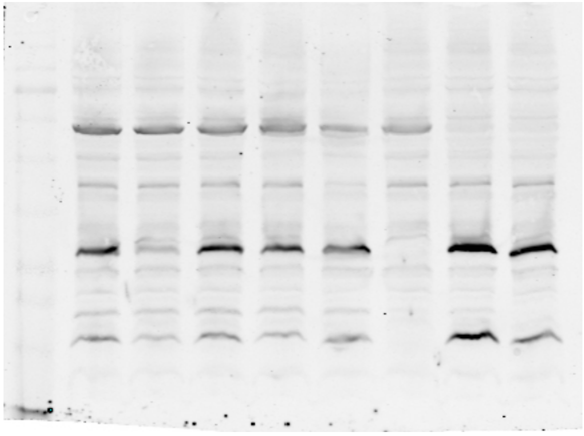

YME1L

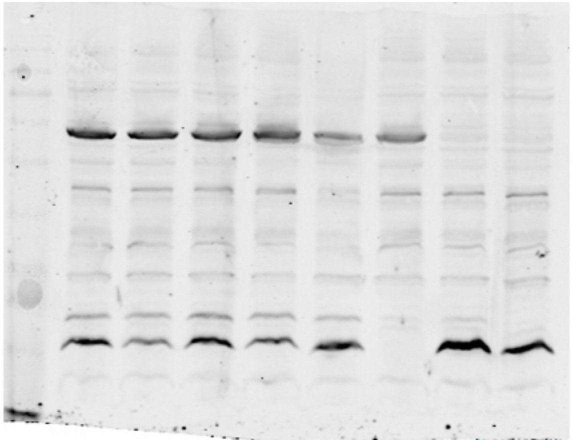

TIM23

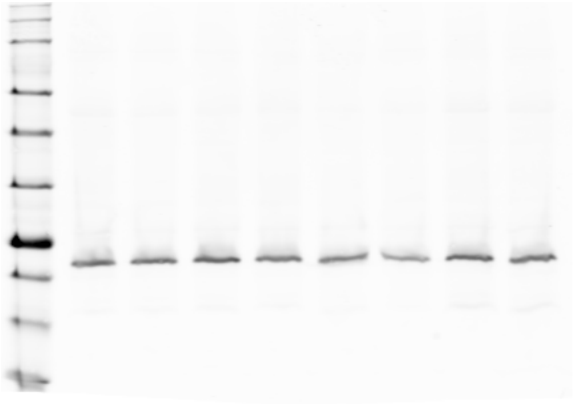

FIGURE 4E Whole Gels

TIM17A

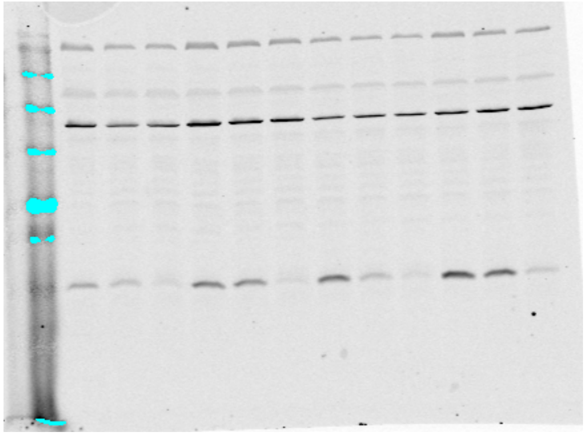

PRELID1

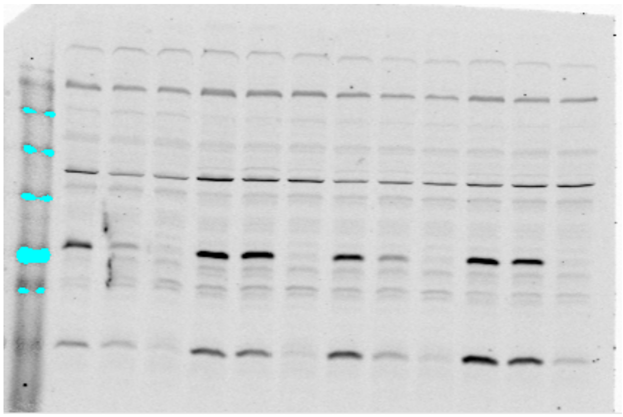

YME1L

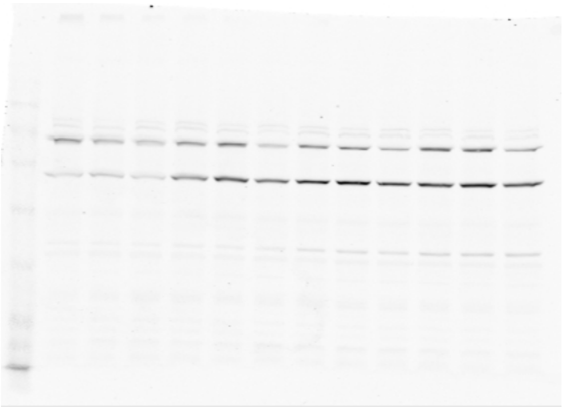

PERK

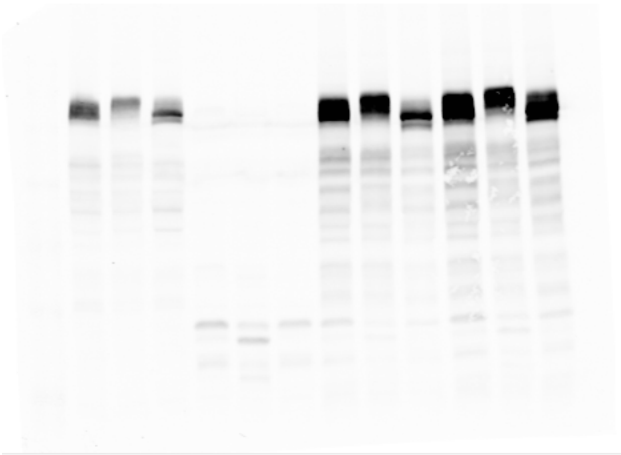

Supplement: Supplementary file 5 — Source Data for Figure 4 [file EMBJ-42-e113908-s006.zip › Figure 4.pdf]
