# Supplementary material for: PERK signaling promotes mitochondrial elongation by remodeling membrane phosphatidic acid
Source: EMBO J. 2023 Jun 12;42(15):e113908. doi: 10.15252/embj.2023113908 (PMC10390871; doi:10.15252/embj.2023113908)
Supplement: Supplementary file 6 [file EMBJ-42-e113908-s005.pdf]

THE EMBO JOURNAL

PERK Signaling Promotes Mitochondrial Elongation By Remodeling Membrane Phosphatidic Acid

Valerie Perea, Christian Cole, Justine Lebeau, Vivian Dolina, Kelsey Baron, Aparajita Madhavan, Jeffery Kelly, Danielle Grotjahn, and Rockland Wiseman
DOI: 10.15252/embj.2023113908

Corresponding author(s): Rockland Wiseman (wiseman@scripps.edu)

Review Timeline:

Transfer from Review Commons:	28th Feb 23
Editorial Decision:	3rd May 23
Revision Received:	5th May 23
Accepted:	12th May 23

Review COMMONS

Editor: William Teale

Transaction Report: This manuscript was transferred to The EMBO JOURNAL following peer review at Review Commons.

Review #1

1. Evidence, reproducibility and clarity:

Evidence, reproducibility and clarity (Required)

Summary:

This study investigates changes in mitochondrial morphology in response to ER stress due to pharmacological inhibition or genetic dysfunction in vitro via two different cell models (MEFs and HeLa cells). The authors specifically implicate the PERK branch of the ER-stress induced pathway in this process based on the observation that mitochondria elongate in response to thapsigargin (Tg) treatment which is blocked by the pathway inhibitors GSK and ISRIB or by genetic ablation of Perk/PERK. Homozygous knockout cells lacking PERK exhibit a fragmented mitochondrial phenotype even in the absence of Tg, which is rescued by expression of the wildtype but not a hypomorphic allele (PERKPSP). One of the more interesting suppositions of this manuscript is that mitochondrial elongation is dependent on the abundance of phosphatidic acid (PA); treatment with Tg provokes an increase in mitochondrial PA, but PA does not accumulate in mitochondria from cells co-treated with GSK, an inhibitor of PERK. This correlation suggests that increased mitochondrial PA accumulation is PERK-dependent. In addition, predicted manipulation of PA levels achieved by a gain of function expression of the lipase Lipin diminished mitochondrial elongation in response to ER stress. Similar results were obtained by PA-PLA1 overexpression, a cytosolic lipase that converts PA into lysophosphatidic acid (LPA). To further describe the mechanistic link between ER stress and mitochondrial morphology, the authors found that PRELID1, which transports PA from the OMM to the intermembrane space, and TIM17A, a component of the protein translocation machinery, were stabilized by loss of PERK or YME1L [and possibly an effect of ATF4], regardless of ER stress via Tg treatment. The authors also report that Tg treatment prevents OPA1 cleavage in cells treated with CCCP, an uncoupler of the proton gradient, suggesting that the effect due to Tg treatment is not through ER stress but decreased mitochondrial fusion via mito-stress induced OPA1 cleavage. To address this, cells were treated with ionomycin which induces mitochondrial fragmentation independent of DRP1. The authors observed an increase in mitochondrial fragmentation in the presence of ionomycin. However, co-treatment with Tg prevented fragmentation, as did overexpression of mitoPLDGFP, which converts cardiolipin to PA on the OMM. These results support a model in which, under ER stress conditions, PERK activation leads to translational attenuation, which leads to a decrease in the steady state levels of PRELID1 via YME1L-dependent

degradation and to the accumulation of PA on the OMM. Based on published work this PA accumulation is expected to inhibit the mitochondrial division dynamin, DRP1. The authors tested this by examining the dependence of mitochondrial elongation on PRELID1.

Major comments:

1. Are the key conclusions convincing?
A considerable amount of work was performed by the authors in preparation of this manuscript and while we find the model exciting, there are several issues that need to be addressed in order for the model to be sufficiently supported.
1. Image quality of mitochondria is sub par and the images do not always appear representative of/match the accompanying histograms. When using a single fluorescent marker (mito-GFP), the images should be in grey scale.
2. Mitochondria in Perk-/- MEFs are highly fragmented, which is potentially inconsistent with previous work (Lebeau J, et al. 2018) performed by the same research group. Can the authors comments on this discrepancy? Also, do the authors interpret this fragmentation to mean that Perk is required to maintain mitochondrial elongation in the absence of exogenous ER stress (Tg)? If so, the authors should test whether expression of a dominant negative version of DRP1 rescues this fragmented morphology. This would be an additional critical test of the authors' model.
3. The authors postulate that mitochondrial elongation in response to Perk activation is specifically outer membrane PA-dependent negative regulation of DRP1. However, PA is readily convertible to other phospholipids, notably CL and LPA, both of which positively regulate mitochondrial fusion. The authors do not measure abundance of other phospholipids, particularly LPA or CL in their targeted lipidomics experiments, only PC. The authors need to consider this alternate possibility.
4. In Figure 5, the authors found very little difference in mitochondrial elongation following knockdown of Prelid1 (comparison between vehicle only conditions), which is potentially inconsistent with their model as decreased PRELID1 should lead to increased OMM PA [and subsequently mitochondrial fusion/elongation].
5. The manuscript requires more careful editing - there were grammatical and punctuation errors.
2. Should the authors qualify some of their claims as preliminary or speculative, or remove them altogether?
In Figure 5, the authors found very little difference in mitochondrial elongation following knockdown of Prelid1 (comparison between vehicle only conditions), which is potentially inconsistent with their model as decreased PRELID1 should lead to increased OMM PA [and subsequently mitochondrial fusion/elongation]. Therefore, these findings do not adequately support the authors' main model.

3. Would additional experiments be essential to support the claims of the paper? Request additional experiments only where necessary for the paper as it is, and do not ask authors to open new lines of experimentation.
- a. In order to further investigate the contribution PRELID1-dependent accumulation of PA in the OMM and its role in mitochondrial elongation, the authors should investigate the abundance of PA (and other lipids) in Perk, Prelid, Yme1l KO mutants. These experiments should quantitatively complement the results in Figure 5. KD of Prelid would be expected to increase mitochondrial elongation but there is no difference compared to WT in Figure 5.
- b. The main premise is that ER-stress activates PERK which in turn leads to increased abundance of PA at the OMM in a PRELID1-dependent manner. PA has been shown to inactivate DRP1, resulting in decreased fission (and mitochondrial elongation). The authors should test their model by expressing a dominant negative allele of DRP1 to see if it rescues the fragmented morphology of Perk KO mutant.
4. Are the suggested experiments realistic in terms of time and resources? It would help if you could add an estimated cost and time investment for substantial experiments.
- a. The authors have all the necessary cell line and methods in hand, so we consider these experiments to be doable.
5. Are the data and the methods presented in such a way that they can be reproduced?
- a. Not all are described in a way that could be easily reproduced (see specific comments below).
6. Are the experiments adequately replicated and statistical analysis adequate?
- a. The foundation of this paper is based on qualitative analysis of confocal fluorescence microscopy images, but the chosen images are often not of high quality so performing statistical analysis in these cases is misleading. Also, each imaging-based experiment was performed three times, but with only 20 cells for each replicate. Does this represent sufficient statistical power?

Specific major comments by section

Introduction
- No additional major comments.

Results
- Title of the subsection: "hypomorphic PERK variants inhibit ER..." is inappropriate since authors only investigated a single hypomorphic variant (PSP). KO mutant is a null not hypomorphic mutant.

Discussion
- Can the authors elaborate on the possible biological relevance for the inhibition of

OPA1 cleavage via Tg treatment?
- PRELID is a known short-lived protein; can the authors elaborate on possible additional impact due to 3-6 hr Tg treatment which is sufficient to induce expression of ATF4 target genes (Figure S2G).
- Thapsigargin induced ER stress does not only activate PERK arm of the ISR, correct? Could the authors comment on this?

Methods
- Addition of drugs and duration (3-6 hrs) likely very toxic to cells; how does this treatment affect viability? Unhealthy cells will have unhealthy mitochondria so it's hard to be confident that subtle morphological differences are specific. Why do authors use 3 hrs Tg-treatment after initially using 6 hrs in Figure 1? Would be helpful to assay toxicity and mitochondrial morphology of thapsigargin and other drugs in WT vs. Perk KO MEFs over time. Previously, an increase in fragmentation was observed at 0.5 hours but this subsided by 6 hours in WT (Lebeau J, et al. 2018) but is this the same for Perk KO MEFs?
Figures/supplementary figures
- General:
- In several images there is substantial background GFP signal resulting in images that are fuzzy on the high quality PDF (printout is unintelligible).
- Example: Figure 2, Mock+veh.
- Example: Figure S2I, Mock+veh, +PA-PLA Tg.
- Example: Figure 3C mock+veh.
- Mitochondrial morphology doesn't appear uniform even within the same cell so how is this accounted for in scoring of mitochondrial morphology? Also, how are authors scoring mitochondrial morphology? Due to the inconsistencies in the chosen images, we feel this manuscript would benefit from addition of a supplementary figure showing examples for each cell model expressing mtGFP (i.e. HeLa and MEFs) depicting the fragmented, tubular and elongated mitochondria. This should be able to be constructed from images already collected for these analyses that weren't already used in the paper.
- Images from prior paper (Lebeau J, et al. 2018) are of much higher quality and is much easier to discern mitochondrial phenotype.
- How much protein was loaded per lane and what was the percentage of polyacrylamide gel? Please clarify details in methodology.
- Figure 1:
- See general comments.
- Figure 1A is virtually identical to Figure 2A (with exception of "MEF A/A") from previous publication: Lebeau J, Saunders JM, Moraes VWR, Madhavan A, Madrazo N, Anthony MC, Wiseman RL. The PERK Arm of the Unfolded Protein Response Regulates Mitochondrial Morphology during Acute Endoplasmic Reticulum Stress.

Cell Rep. 2018 Mar 13;22(11):2827-2836. doi: 10.1016/j.celrep.2018.02.055. PMID: 29539413; PMCID: PMC5870888.

- Figure 1B: the complemented Perk KO + vehicle should be similar to WT vehicle, but those images look quite different, even so, the respective bars are equal.
- Vehicle treated Perk-/- cells have fragmented morphology which is different from Figure 2F in above publication by same group. Can the authors explain this discrepancy?
- Figure S1:
- No additional major comments.
- Figure 2:
- See general comments.
- If the authors' hypothesis is correct, overexpression of PRELID1 should have same effect as overexpression of Lipin.

● Figure S2:

- Images in Figure S2I are not representative of corresponding bars in Figure S2J (specifically vehicle treated panels). The "+PA-PLA1+Tg" panel instead appears fragmented (in comparison with other images).
- Do authors have clearer images to substitute for CHX-treated panels?

● Figure 3:

- What is the selective marker used for HeLa cells expressing mitoPLDGFP since the HeLa parental cell background already expressed a mitochondrial targeted GFP, we assume it was puromycin but this was not clear in the Figure legend or methods? If so, it would be helpful to clarify this. If not, how can the authors observe a difference in morphology if the selectable marker is the same? Indeed, mitoPLDGFP is expressed, detectable by immunoblot, but this is on a cell population level so no way of knowing whether the specific cells scored expressed mitoPLDGFP unless another selectable marker was used (i.e. should have used CFP, RFP, etc.).
- The authors state "Note the expression of mitoPLDGFP did not impair our ability to accurately monitor mitochondrial morphology in these cells." in Figure 3 legend and again basically the same in S3: "Note that the expression of the mitoPLDGFP did not impair our ability to monitor mitochondrial morphology in these cells." Could the authors explain their reasoning here?
- Figure S3:
- Same as in Figure 3; "mock+Veh" appears more fragmented than tubular so is there a more representative image that the authors can show?
- Figure 4:
- No major comments.
- Figure S4:
- Figure S4C: the authors show that Tg treatment on MEF mtGFP cells for distinct hours to determine PRELID levels. However, in the Results section states that this treatment was with CHX, could the authors please check this and correct?

- Figure 5:
- 5C: PLKO NS shRNA +Tg appears more fragmented than tubular; do the authors have a more representative image?
- Figure S5:
- No major comments.
- Figure 6:
- A schematic representation should be a graphic summary of all findings reported in the text with no text except where absolutely essential. A good model should be easily understood without reading any description since all concepts were supported in the main text and by experimentation.
- The model also contains some inaccuracies. The suggestion is that the authors re-do the model and clarify some aspects such as:
- The model suggests that ISRIB inhibits PRELID1 directly but there is no evidence for this whereas PRELID is directly regulated by YME1L (also typo here in figure: "Yme1" no "l").
- This model incorrectly uses inhibition symbols; for example, mutation of Perk does not inhibit its activity as GSK does. The KO does not have Perk so cannot perform its function. These are not the same. Similarly, the lipases (Lipin and PA-PLA1) should be depicted instead as altering flux of PA away from OMM not as inhibition.
- The authors should connect PA accumulation in the OMM graphically to mitochondrial elongation [instead of through text]. If the authors consider the numbered labels convenient, please use just the number and place the description in the figure legend instead.

Minor comments:

1. Specific experimental issues that are easily addressable.
- a. Yes, please see specific examples below.
2. Are prior studies referenced appropriately?
- a. References appeared adequate except in the Materials and Methods section (see specific examples below).
3. Are the text and figures clear and accurate?
- a. No, the text needs considerable editing to make the language clearer and formal whereas the figures are not always presented in a manner that is easily absorbed by the reader. Representative microscopy images chosen do not always match the corresponding graphical summary and are not clear even on PDF version compared to (Lebeau J, et al. 2018 - full citation above).
4. Do you have suggestions that would help the authors improve the presentation of their data and conclusions?
- a. Yes, please see specific examples below.

Specific minor comments by section

Introduction

- This section contains minor grammatical errors and awkward writing which should be rephrased to be more concise. For example:
- Incorrect use of commas (ex: absence of commas on page 3, bottom of paragraph 3).

Results

- Overall, this section contains many grammatical errors and awkward language but these are unevenly distributed as some subsections are well written and thoroughly edited whereas others need closer inspection. For example:
- No period at end of first subsection title; this should be consistent throughout.
- Text not consistently written in past tense/passive voice.
- Post-translational should be hyphenated (page 5, 2x on bottom of page).
- The use of dashes to conjoin thoughts is too casual and sentences should be restructured with the aid of parentheses or semicolons only when necessary (ex: page 6, paragraph 2 through page 7).
- Homogenize the use of hyphens in all sentences such as: ER stress-induced, ER stress-dependent.

Discussion

- Minor grammatical errors and awkward wording throughout; description of ideas should be more concisely written. For example:
- Page 13, paragraph 1: "Thus, an improved understanding of how different PERK-dependent alterations to mitochondrial morphology and function integrate will provide additional insight to the critical importance of this pathway in regulating mitochondria during conditions of ER stress."
- Page 13, paragraph 2: "Further investigations will be required to determine the specific impact of altered PERK signaling on mitochondria morphology and function in the context of these diseases to reveal both the pathologic and potentially therapeutic implications of PERK activity on the mitochondrial dysfunction observed in the pathogenesis of these disorders."
- Awkward/oxymoronic word choices. For example:
- Page 11, paragraph 2: "...GSK2606414 reduces Tg-dependent increases of PA..." could be written as "... blocks/limits Tg-dependent increase of PA..." instead.
- What is evidence that ionomycin is completely independent of DRP1?

Methods

- Please provide more description or a reference for the method used for CRISPR/Cas9 gene editing (page 15, paragraph 1).
- Since different versions of chemicals are often available from the same company (for example in solution vs. powder, as a salt, different purities, etc.) it would be helpful for the authors to also include the catalog number for the purchased drugs and analytical standards (page 16, paragraph 1).
- The authors did an excellent job of blinding these images and utilizing several researchers to score each. However, we feel that 20 cells per biological replicate (~60 total per condition) is insufficient when mitochondrial morphology in chosen representative images is unclear. We think it is reasonable to request the authors to score additional images they collected as part of this investigation.
- The below two sentences contain some redundancies and should be combined/rephrased (page 16, paragraph 2).
- "Three different researchers scored each set of images and these scores were averaged for each individual experiment. All quantifications shown were performed for at least 3 independent experiments, where averages in morphology quantified from each individual experiment were then combined."
- Incorrect units, for example: "500g" should be "500 x g" on page 16, paragraph 3 and "g" should be italicized. Same for "200g" on page 17, paragraph 1.
- Inconsistent abbreviation of chemicals, for example:
- Chloroform and hydrochloric acid but not methanol in methods on page 17, paragraph 1. Also, the "l" in "HCL" should be lowercase.
- "Solvents" (2x) on page 17, paragraph 2 should be singular not plural.
- What does RT stand for on page 17, paragraph 2?
- Tris buffered saline is abbreviated incorrectly as "TB" then correctly later in the same paragraph as "TBS" on page 18, paragraph 3.
- Paragraph 4 on page 18 should be indented to be consistent with formatting of previous methods sections.
- To remove any ambiguity, catalog numbers should be included for antibodies (also consider including the lot number as there can be lot to lot variability).
- What percentage of tween v/v was supplemented in TBS buffer? Different concentrations of tween can impact antibody binding and would beneficial to include for reproducibility.
- Please indicate the incubation time and conditions for the secondary antibodies.
- The abbreviation for phosphate buffered saline is "PBS" not "PBD" (page 19, paragraph 1).
- Could the authors state clearly the reference transcript used for RT-qPCR (assumed is RIBOP)?
- Sometimes GIBCO is capitalized, sometimes not (Gibco), which should also be consistent.
- Who is the supplier for CCCP and what is the catalog number? Similarly, what is the

catalog number for TMRE (both on page 19, paragraph 3)?
- Student's t-test is capitalized and possessive (similar to Tukey's) on page 19, paragraph 4.

Figures/supplementary figures

- General:
- With respect to the lines overlaying histograms scoring mitochondrial morphology for designating statistical significance [with color-coded asterisks]:
- It is assumed that the bars of the histogram being compared are those at the ends of each line but these aren't aligned perfectly. Please tidy up the figure by shifting these and consider capping lines to make more clear.
- It appears that the authors provide these lines at all instances of statistically significant differences whether the comparison is important to their conclusions or not; including only the necessary comparisons will reduce the noise of these figures and make them easier to absorb and interpret. For example:
- Figure 1C: why is comparison being made only for KO vs. complemented (+veh) - difference between KO and WT not statistically significant? Also, wouldn't the difference between WT and KO +Tg percent fragmented be statistically significant? The comparisons being made appear arbitrary or if not, was not clearly stated (same criticism for 2D, 3B, 3D, etc.).
- The authors appear to use "transfection" and "transduction" interchangeably such that it is unclear whether expression of transgenes or shRNA is stably vs. transiently expressed. It would help if the authors could clarify their language here as well.
- Figure 1:
- Figure 1A - PERK is membrane bound not soluble; should this not be represented in the model? Model colors are not easily distinguishable from each other on printout and should be upgraded.
- Figure 1C - phenotypic scoring is not easy to interpret; perhaps authors could rearrange the figure such that each treatment is adjacent since that is the more interesting comparison? All cells in figure 1 are MEFs so delete "MEFs" below Perk+/+ and Perk -/-.
- Figure S1:
- How much protein was loaded per lane and what percentage of polyacrylamide gel was used?
- Figure 2:
- See general comments.
- Figure 2A - extra letter/typo in "Fold Change."
- Why do authors switch to HeLa cells after measuring PA content in MEFs?
- Figure S2:
- Authors are now including ns for "not significant" and the p value where before they

were not before. The intent for including the p-value in S2B appears to be because it suggests a trend towards statistical significance (actually a bit surprised it is not based on SEM error bars; authors should recheck their calculations) which is inappropriate. Either provide all the p-values, possibly as a separate table or none at all.
- Now including double headed error bars for S2D-E which is inconsistent with rest of manuscript.
- What is standard error for vehicle treated cells in 3B, 3D, and 3E? Given the above mistake it's reasonable to suspect that the error bars were omitted by accident.
- Figure 3:
- Title should have hyphen for "stress-induced" and ionomycin shouldn't be capitalized.
- Now using double headed error bars for 3B which is inconsistent with majority of other figures.
- Figure S3:
- Title should have hyphen for "stress-induced" and ionomycin shouldn't be capitalized.
- Figure 4:
- What is the purpose of including 4A? This depicts a concept which is not particularly difficult to grasp, was not experimentally shown in this manuscript, and is somewhat redundant with Figure 6. We recommend removing from Figure 4 and combining with Figure 6.
- Since all cells used in Figure 4 were MEFs, the authors can remove "MEFs" from figure and just include genotype.
- Figure 4C: typo in Yme1l - has two 1's.
- Figure S4:
- See general comments.
- Figure 5:
- Figure 5C: What does PLKO abbreviation stand for in the control line? pLKO.1 vector (see methods but not explained further).
- Figure S5:
- Figure S5A-B: KD clearly worked but how efficient is unclear (quantitatively, i.e. 50, 90%, etc.?). The authors could perform serial dilutions of protein (i.e. 5, 10, 20 ug of the same samples for SDS-PAGE/immunoblot) or RT-qPCR. If knockdown is incomplete, this could explain the discrepancy in Figure 5 where depletion of Prelid should result in elongation [via OMM depletion of PA].
- Figure 6:
- This is a more appropriate location for panel 4A.

2. Significance:

Significance (Required)

1. Describe the nature and significance of the advance (e.g. conceptual, technical, clinical) for the field.
- a. Perturbances in PERK signaling evoke an alteration in mitochondrial morphology and have been extensively reported on, due to their clinical implications on neurodegenerative disorders such as Alzheimer's disease. The present work provides insight into the molecular basis for Stress Induced Mitochondrial Hyperfusion (SIMH) which can be triggered by ER stress. The authors find that this process occurs downstream of PERK and proceeds through accumulation of PA in the OMM by stabilization of Prelid, a mitochondrial resident protein that transports PA from the OMM to IMM for cardiolipin synthesis. The evidence of this work represents a substantial addition to the field of mitochondrial dynamics/SIMH and the Unfolded Protein Response.
2. Place the work in the context of the existing literature (provide references, where appropriate).
- a. The novelty of this work is in the inclusion of PRELID1 downstream of PERK signaling pathway for transmission of ER stress to the mitochondria, a process that involves phosphatidic acid (PA). Some studies have addressed how phosphatidic acid is a modulator and a signal in mitochondrial physiology. The role of the lipids in mitochondrial dynamics represent an important and emerging field that needs to be explored in order to understand how metabolites control mitochondrial fusion/fission.

References

Yoshihiro Adachi, Kie Itoh, Tatsuya Yamada, Kara L. Cerveny, Takamichi L. Suzuki, Patrick Macdonald, Michael A. Frohman, Rajesh Ramachandran, Miho Iijima, Hiromi Sesaki. Coincident Phosphatidic Acid Interaction Restrains Drp1 in Mitochondrial Division. Molecular Cell. Volume 63, Issue 6. 2016. Pages 1034-1043. https://doi.org/10.1016/j.molcel.2016.08.013

Huang H, Gao Q, Peng X, Choi SY, Sarma K, Ren H, Morris AJ, Frohman MA. piRNA-associated germline nuage formation and spermatogenesis require MitoPLD profusogenic mitochondrial-surface lipid signaling. Dev Cell. 2011 Mar 15;20(3):376-87. https://doi.org/10.1016/j.devcel.2011.01.004
3. State what audience might be interested in and influenced by the reported findings.
- a. Audiences of the fields such as Mitochondrial dynamics, UPR, lipid metabolism, neurodegenerative diseases, ER-stress response, Integrated Stress Response.
4. Define your field of expertise with a few keywords to help the authors contextualize your point of view. Indicate if there are any parts of the paper that you do not have sufficient expertise to evaluate.
- a. Mitochondrial morphology, mtDNA inheritance, mitochondrial metabolism, fluorescence/indirect immunofluorescence microscopy

3. How much time do you estimate the authors will need to complete the suggested revisions:

Estimated time to Complete Revisions (Required)

(Decision Recommendation)

Between 1 and 3 months

4. *Review Commons* values the work of reviewers and encourages them to get credit for their work. Select 'Yes' below to register your reviewing activity at [Web of Science Reviewer Recognition Service] (formerly Publons); note that the content of your review will not be visible on Web of Science.

Web of Science Reviewer Recognition

No

Review #2

1. Evidence, reproducibility and clarity:

Evidence, reproducibility and clarity (Required)

Summary

Previous studies have shown that ER stress increases amounts of phosphatidic acid (PA) (PMID: 22493067) and induces elongation of mitochondria through the protein and lipid kinase PERK (PMID: 29539413, work by Wiseman's lab). The current work reports that ER stress by thapsigargin promotes the degradation of a mitochondrial protein PRELID1, which transfers PA from the outer membrane to the inner membrane. An inner membrane protease, YME1L, was identified as responsible for this degradation of PRELID1. Consistent with the notion that PA is required for the morphological change, overexpression of a PA phosphatase (Lipin) or a PA

phospholipase (PA-PLA1) decreased ER-stress-induced mitochondrial elongation.

Specific comments

1. The authors report that PRELID1 knockdown did not promote mitochondrial elongation under either normal or ER-stress conditions (Fig. 5). If PRELID1 plays a vital role in mitochondrial elongation, PRELID1 depletion will restore elongation. Therefore, the presented data argue against the authors' conclusion. Since PRELID1 has multiple homologs, including PRELID3B, which is also a short-lived protein like PRELID1, these homologs might redundantly function in PA transport, especially when PRELID1 is absent. Therefore, the authors need to knock them down simultaneously. This possibility is consistent with the previous authors' data that YME1L depletion decreases ER-stress-induced mitochondrial elongation (PMID: 29539413). YME1L knockdown may rescue multiple short-lived PRELID1 homologs.
2. Another possibility is that since a previous study has shown that PERK-produced PA activates the mTOR-AKT pathway (PMID: 22493067), this signaling pathway may contribute to mitochondrial morphology in addition to PRELID1. The authors should test the combined effects of mTOR-AKT inhibition in ER-stress-induced mitochondrial elongation.
3. The authors' model suggests the loss of PRELID1 increases PA levels in the mitochondrial outer membrane (Fig. 6). The authors should test PA levels in mitochondria isolated from cells depleted for PRELID1 and its homologs (simultaneously). Since PA that is transported to the inner membrane is actively converted to other phospholipids, such as CDP-DAG, elevated levels of PA are likely seen if the outer membrane to inner membrane transport is blocked.
4. The authors need to test whether Lipin and PA-PLA1 overexpression decreased PA levels in mitochondria treated with thapsigargin. The current manuscript only shows the effect of Lipin and PA-PLA1 on PA levels in whole-cell lysate without ER stress (Fig. S2F).
5. The authors propose that PA inhibits DRP1 in mitochondrial division under ER stress. It has been shown that PA blocks DRP1 after recruitment to mitochondria (PMID: 27635761). Does thapsigargin induce mitochondrial accumulation of DRP1?

2. Significance:

Significance (Required)

Overall, this manuscript is a nice extension of the authors' previous work and investigates the molecular mechanism underlying the regulation of mitochondrial elongation induced by ER stress. However, the current data do not strongly support

the role of PRELID1 in either ER-stress-mediated PA level elevation or mitochondrial elongation, as described in Specific comments. The authors should address these points.

Audience
ER stress, mitochondrial dynamics, membrane lipids, proteases

My Expertise
mitochondrial dynamics, lipid biology

3. How much time do you estimate the authors will need to complete the suggested revisions:

Estimated time to Complete Revisions (Required)

(Decision Recommendation)

Between 3 and 6 months

4. *Review Commons* values the work of reviewers and encourages them to get credit for their work. Select 'Yes' below to register your reviewing activity at Web of Science Reviewer Recognition Service (formerly Publons); note that the content of your review will not be visible on Web of Science.

Web of Science Reviewer Recognition

No

Review #3

1. Evidence, reproducibility and clarity:

Evidence, reproducibility and clarity (Required)

The authors investigated signaling pathways and molecular mechanisms leading to mitochondrial dysfunction after ER stress. This study extends their previous publication (Lebeau et al., 2018) by providing evidence on how PERK regulates mitochondrial structure and function in response to ER stress. Some key findings are that PERK induces mitochondrial elongation by increasing and retaining phosphatidic acid (PA) in the outer mitochondrial membrane which is important for cell adaptation and survival. This process requires PERK-dependent translational attenuation through YME1L-PRELID dependent mechanism.

This is a very strong study with compelling evidence. Only one minor point should be addressed:
In Fig S2G & H, the authors indicate that "Lipin1 overexpression did not significantly influence increases of ATF4 protein". The blots show a decrease in ATF4 in Tg-treated HeLa cells. The same effect is observed in Fig. S3F showing reduction in ATF4, but the authors described it as the "overexpression of mitoPLD did not significantly impact other aspects of PERK signaling in Tg-treated cells". The quantification of the blots or indication that the blots were quantified should be clarified and noted (at least in the legend).

2. Significance:

Significance (Required)

This study adds to our current knowledge on how ER stress affects mitochondria adaptation and proteostasis, which may contribute to the pathogenesis and progression of numerous neurodegenerative diseases. Specifically, this study establishes a new role for PERK in mitochondrial adaptive remodeling focused on trafficking and accumulation of phospholipids. Identifying molecular markers like PERK and its involvement with PRELID, YME1L, and PA to regulate mitochondrial remodeling during ER stress is important to understand the effects of drug-targeting this ER stress-responsive factor.

3. How much time do you estimate the authors will need to complete the suggested revisions:

Estimated time to Complete Revisions (Required)

(Decision Recommendation)

Less than 1 month

4. *Review Commons* values the work of reviewers and encourages them to get credit for their work. Select 'Yes' below to register your reviewing activity at Web of Science Reviewer Recognition Service (formerly Publons); note that the content of your review will not be visible on Web of Science.

Web of Science Reviewer Recognition

Yes

Full Revision

Manuscript number: RC-2022-01318
Corresponding author(s): Rockland Luke Wiseman

[Please use this template only if the submitted manuscript should be considered by the affiliate journal as a full revision in response to the points raised by the reviewers.

If you wish to submit a preliminary revision with a revision plan, please use our "Revision Plan" template. ***It is important to use the appropriate template to clearly inform the editors of your intentions.****]*

1. General Statements [optional]

Please find included a revised manuscript titled "***PERK Signaling Promotes Mitochondrial Elongation By Remodeling Membrane Phosphatidic Acid***" that we would like considered for publication in EMBO. This manuscript was originally submitted and reviewed through Review Commons (RC-2022-01318).

In this manuscript, we describe a molecular mechanism to explain endoplasmic reticulum (ER) stress dependent induction of adaptive mitochondrial elongation. We show that ER stress-dependent increases in mitochondria elongation are dependent on the PERK signaling arm of the unfolded protein response (UPR). Our data demonstrate that PERK promotes mitochondrial elongation through a mechanism involving both PERK-dependent increases in phosphatidic acid (PA) and selective YME1L-dependent reductions in the mitochondrial PA trafficking protein PRELID1 downstream of PERK-dependent translation attenuation. This results in both a reduction in PA at the inner mitochondrial membrane where it is converted to cardiolipin and an increase in PA on the outer mitochondrial membrane where it inhibits mitochondrial fission to increase mitochondrial length. These results establish a new role for PERK signaling in regulating mitochondrial phospholipids and morphology during acute ER stress.

We were very enthusiastic by the positive reviewer response to the initial submission of our manuscript to Review Commons. We address all the reviewers' valuable comments in this revised manuscript through the inclusion of 27 new or improved figure panels and significant revision to the text. Our response to specific comment are described in the ***Our Response to Reviewer Comments*** document included with this revision. We feel strongly that the manuscript has been significantly improved through the review process and is now ready for publication.

Please note that the long time it took to complete this revision was a result of numerous issues out of our control and independent of the research described in this manuscript.

2. Point-by-point description of the revisions

This section is mandatory. Please insert a point-by-point reply describing the revisions that were already carried out and included in the transferred manuscript.

We apologize for the delay in resubmitting this revised manuscript. We faced a number of challenges over the previous year unrelated to this project that slowed progress on completing the necessary revisions. However, we are happy to report we have addressed all of the reviewer's valuable comments in this revised submission through the including of 27 new or improved figure panels and significant adaptations to the text. We highlight these changes below.

REVIEWER #1.

Reviewer #1 General Comments. *"This study investigates changes in mitochondrial morphology in response to ER stress due to pharmacological inhibition or genetic dysfunction in vitro via two different cell models (MEFs and HeLa cells). The authors specifically implicate the PERK branch of the ER-stress induced pathway in this process based on the observation that mitochondria elongate in response to thapsigargin (Tg) treatment which is blocked by the pathway inhibitors GSK and ISRIB or by genetic ablation of Perk/PERK. Homozygous knockout cells lacking PERK exhibit a fragmented mitochondrial phenotype even in the absence of Tg, which is rescued by expression of the wildtype but not a hypomorphic allele (PERKPSP). One of the more interesting suppositions of this manuscript is that mitochondrial elongation is dependent on the abundance of phosphatidic acid (PA); treatment with Tg provokes an increase in mitochondrial PA, but PA does not accumulate in mitochondria from cells co-treated with GSK, an inhibitor of PERK. This correlation suggests that increased mitochondrial PA accumulation is PERK-dependent. In addition, predicted manipulation of PA levels achieved by a gain of function expression of the lipase Lipin diminished mitochondrial elongation in response to ER stress. Similar results were obtained by PA-PLA1 overexpression, a cytosolic lipase that converts PA into lysophosphatidic acid (LPA). To further describe the mechanistic link between ER stress and mitochondrial morphology, the authors found that PRELID1, which transports PA from the OMM to the intermembrane space, and TIM17A, a component of the protein translocation machinery, were stabilized by loss of PERK or YME1L [and possibly an effect of ATF4], regardless of ER stress via Tg treatment. The authors also report that Tg treatment prevents OPA1 cleavage in cells treated with CCCP, an uncoupler of the proton gradient, suggesting that the effect due to Tg treatment is not through ER stress but decreased mitochondrial fusion via mito-stress induced OPA1 cleavage. To address this, cells were treated with ionomycin which induces mitochondrial fragmentation independent of DRP1. The authors observed an increase in mitochondrial fragmentation in the presence of ionomycin. However, co-treatment with Tg prevented fragmentation, as did overexpression of mitoPLDGFP, which converts cardiolipin to PA on the OMM. These results support a model in which, under ER stress conditions, PERK activation leads to translational attenuation, which leads to a decrease in the steady state levels of PRELID1 via YME1L-dependent degradation and to the accumulation of PA on the OMM. Based on published*

work this PA accumulation is expected to inhibit the mitochondrial division dynamin, DRP1. The authors tested this by examining the dependence of mitochondrial elongation on PRELID1."

"Perturbances in PERK signaling evoke an alteration in mitochondrial morphology and have been extensively reported on, due to their clinical implications on neurodegenerative disorders such as Alzheimer's disease. The present work provides insight into the molecular basis for Stress Induced Mitochondrial Hyperfusion (SIMH) which can be triggered by ER stress. The authors find that this process occurs downstream of PERK and proceeds through accumulation of PA in the OMM by stabilization of Prelid, a mitochondrial resident protein that transports PA from the OMM to IMM for cardiolipin synthesis. The evidence of this work represents a substantial addition to the field of mitochondrial dynamics/SIMH and the Unfolded Protein Response"

"The novelty of this work is in the inclusion of PRELID1 downstream of PERK signaling pathway for transmission of ER stress to the mitochondria, a process that involves phosphatidic acid (PA). Some studies have addressed how phosphatidic acid is a modulator and a signal in mitochondrial physiology. The role of the lipids in mitochondrial dynamics represent an important and emerging field that needs to be explored in order to understand how metabolites control mitochondrial fusion/fission."

Our Response to Reviewer #1 General Comments. We thank the reviewer for the positive comments related to our manuscript. We address specific comments brought up by the reviewer in our revised manuscript as highlighted below. We combined specific comments related to the same point in this response to best manage the various points brought up by the reviewer.

Reviewer #1 Comment #1. The ***Reviewer*** brought up the quality of our images numerous times in their review. A few examples are included below.

"*Image quality of mitochondria is sub par and the images do not always appear representative of/match the accompanying histograms. When using a single fluorescent marker (mito-GFP), the images should be in grey scale.*"

"*In several images there is substantial background GFP signal resulting in images that are fuzzy on the high quality PDF (printout is unintelligible). Example: Figure 2, Mock+veh. Example: Figure S2I, Mock+veh, +PA-PLA Tg. Example: Figure 3C mock+veh*"

"*Images from prior paper (Lebeau J, et al. 2018) are of much higher quality and is much easier to discern mitochondrial"*

"*Mitochondrial morphology doesn't appear uniform even within the same cell so how is this accounted for in scoring of mitochondrial morphology? Also, how are authors scoring mitochondrial morphology? Due to the inconsistencies in the chosen images, we feel this manuscript would benefit from addition of a supplementary figure showing examples for each cell*

model expressing mtGFP (i.e. HeLa and MEFs) depicting the fragmented, tubular and elongated mitochondria. This should be able to be constructed from images already collected for these analyses that weren't already used in the paper."

Our Response to Reviewer #1 Comment #1. In the revised manuscript, we improved the quality of the images and converted all images to greyscale, as suggested by the reviewer.

As described in *Materials and Methods,* we quantified mitochondrial morphology by cell, scoring whether a cell has primarily fragmented, tubular, or elongated mitochondria morphology. This scoring was performed by at least two blinded researchers for at least 3 independent experiments with a total of >60 cells/condition counted across all experiments. Scores for individual experiments were then combined and averaged. Statistics were calculated from these averaged scores. In the revised manuscript, the images presented are representative of each individual condition. In addition, we now include new panels showing the quantification of total cells counted/condition across all individual replicates by a representative researcher for the main text figures (e.g., see **Fig. S1C**). This provides an alternative representation of the observed phenotype across the individual experiments for these key figures.

As suggested by the reviewer, in the revised submission we also now provide representative images of cells with primarily fragmented, tubular, and elongated mitochondria for both MEF and HeLa cells (**Fig. S1A,B**). We appreciate this suggestion as we feel it improves the clarity of our manuscript.

Reviewer #1 Comment #2. "*Mitochondria in Perk-/- MEFs are highly fragmented, which is potentially inconsistent with previous work (Lebeau J, et al. 2018) performed by the same research group. Can the authors comments on this discrepancy? Also, do the authors interpret this fragmentation to mean that Perk is required to maintain mitochondrial elongation in the absence of exogenous ER stress (Tg)? If so, the authors should test whether expression of a dominant negative version of DRP1 rescues this fragmented morphology. This would be an additional critical test of the authors' model*."

"*Vehicle treated Perk-/- cells have fragmented morphology which is different from Figure 2F in above publication by same group. Can the authors explain this discrepancy*?"

Our Response to Reviewer #1 Comment #2. In our previous publication, we did not quantify mitochondrial morphology in *Perk*-deficient cells. However, as reported in this current manuscript, we find that *Perk*-deficient cells display higher amounts of fragmented mitochondria, as compared to *Perk*$^{+/+}$ MEFs (**Fig. 1B,C**). We quantified this result across 5 independent experiments. Moreover, we found that reconstitution with PERK^{WT} restored tubular mitochondrial morphology in *Perk*-deficient cells, demonstrating that this effect can be attributed to loss of PERK.

With respect to the increase in mitochondrial fragmentation observed in *Perk*-deficient MEFs, we attribute this to reduced mitochondrial membrane potential observed in these cells. We now show that *Perk*-deficient MEFs show 50% reductions in TMRE staining, as compared to controls. We include this data in the revised manuscript as **Fig. S1D** and the accompanying text below.

*Line 91. "Perk$^{-/-}$ MEFs showed increases in fragmented mitochondria in the absence of treatment (**Fig. 1B,C** and **Fig. S1C**). This corresponds with reductions in the mitochondrial membrane potential in Perk-deficient cells, as measured by tetramethylrhodamine ethyl ester (TMRE) staining (**Fig. S1D**). This suggests that the increase of fragmentation in these cells can be attributed to mitochondrial depolarization. Tg-induced mitochondrial elongation was also impaired in Perk-deficient cells (**Fig. 1B,C** and **Fig. S1C**).".*

Reviewer #1 Comment #3. "*The authors postulate that mitochondrial elongation in response to Perk activation is specifically outer membrane PA-dependent negative regulation of DRP1. However, PA is readily convertible to other phospholipids, notably CL and LPA, both of which positively regulate mitochondrial fusion. The authors do not measure abundance of other phospholipids, particularly LPA or CL in their targeted lipidomics experiments, only PC. The authors need to consider this alternate possibility*."

Reviewer #1 Comment #3. Overexpression of PA-PLA1 (which converts PA to LPA) blocks ER stress induced mitochondrial elongation (**Fig. S2L-O**). This indicates that the observed Tg-dependent increase in mitochondrial elongation are unlikely to be attributed to increases in LPA. mitoPLD converts CL to PA at the outer mitochondrial membrane. Since mitoPLD overexpression increases mitochondrial elongation (**Fig. 3A,B**), this again suggests that CL is not a major driver of mitochondrial elongation. These results combined with the sensitivity of ER stress induced mitochondrial elongation to two different PA lipases strongly support a model whereby increases in PA contribute to ER stress induced mitochondrial elongation.

In the revised manuscript, we include measurements of CL in mitochondria isolated from MEF^{mtGFP} cells treated with Tg and/or depleted of *Prelid1*. As expected, reductions of PRELID1 decrease CL in isolated mitochondria (**Fig. S5C**). Treatment with Tg reduced CL to similar extents in mitochondria isolated from MEF^{mtGFP} cells expressing non-silencing shRNA. However, we did not observe further reductions of CL in *Prelid1*-depleted cells. This is consistent with a model whereby ER stress-dependent reductions in PRELID1 decrease PA trafficking across the IMS and lead to reductions in CL synthesis. These results are discussed in the revised manuscript as below:

Line 214. "*PRELID1 traffics PA from the outer to inner mitochondrial membrane, where it serves as a precursor to the formation of cardiolipin.[56,66,67] Thus, reductions in PRELID1 should decrease cardiolipin. To test this, we shRNA-depleted Prelid1 from MEF^{mGFP} cells and monitored cardiolipin in isolated mitochondria in the presence or absence of ER stress. We confirmed efficient PRELID1 knockdown by immunoblotting (**Fig. S5A**). Importantly, Prelid1 depletion did not alter Tg-induced*

*reductions of TIM17A or increases of ATF4. Further, Tg-dependent increases in PA were observed in Prelid1-depleted MEF^{mtGFP} cells (**Fig. S5B**). These results indicate that loss of PRELID1 does not impair PERK signaling in these cells. Prelid1 depletion reduced cardiolipin in mitochondria isolated from MEF^{mtGFP} cells (**Fig. S5B**). Treatment of MEF^{mtGFP} cells expressing non-silencing shRNA with Tg for 3 h also reduced cardiolipin to levels similar to those observed in Prelid1-deficient cells. However, Tg did not further reduce cardiolipin in Prelid-depleted cells. These results are consistent with a model whereby ER stress-dependent reductions in PRELID1 limit PA trafficking across the inner mitochondrial membrane and contribute to reductions in cardiolipin during acute ER stress"*

Reviewer #1 Comment #4. "*In Figure 5, the authors found very little difference in mitochondrial elongation following knockdown of Prelid1 (comparison between vehicle only conditions), which is potentially inconsistent with their model as decreased PRELID1 should lead to increased OMM PA [and subsequently mitochondrial fusion/elongation]. Therefore, these findings do not adequately support the authors' main model.*"

Our Response to Reviewer #1 Comment #4. Our model predicts that ER stress induced mitochondrial elongation is mediated through a process involving *both* PERK kinase-dependent increases in total PA *and* YME1L-dependent PRELID1 degradation induced downstream of PERK-dependent translation attenuation (see **Fig. 6**). Thus, we predict that PRELID1 degradation is *required*, but not *sufficient*, to promote mitochondrial elongation. Our results showing that *PRELID1* depletion does not basally disrupt mitochondrial morphology *or* inhibit Tg-induced mitochondrial elongation are consistent with this model. Moreover, we show that genetic *Prelid1* depletion rescues Tg-induced mitochondrial elongation in cells co-treated with the PERK signaling inhibitor ISRIB – a compound that blocks PERK-dependent PRELID1 degradation (**Fig. 4D**), but not increases in PA (**Fig. 2B, S2E,F**) – in both MEF^{mtGFP} and HeLa cells (**Fig. 5A-D**). This is consistent with our proposed model whereby PRELID1 degradation is *required* but not *sufficient* for promoting mitochondrial elongation. We make this point clearer in the revised manuscript.

*Line 259: "Interestingly, Prelid1 depletion did not basally influence mitochondrial morphology or inhibit Tg-induced mitochondrial elongation (**Fig. 5A,B** and **Fig. S5E**). This indicates that reduction of PRELID1, on its own, is not sufficient to increase mitochondrial elongation, likely reflecting the importance of PERK kinase-dependent increases in PA in this process.[53]"*

Reviewer #1 Comment #5. "*The manuscript requires more careful editing - there were grammatical and punctuation errors.*"

"… the text needs considerable editing to make the language clearer and formal whereas the figures are not always presented in a manner that is easily absorbed by the reader. Representative microscopy images chosen do not always match the corresponding graphical

summary and are not clear even on PDF version compared to (Lebeau J, et al. 2018 - full citation above)."

Our Response to Reviewer #1 Comment #5. We carefully edited the revised manuscript. We also confirmed that representative images match the observed quantifications.

Reviewer #1 Comment #6. "*In order to further investigate the contribution PRELID1-dependent accumulation of PA in the OMM and its role in mitochondrial elongation, the authors should investigate the abundance of PA (and other lipids) in Perk, Prelid, Yme1l KO mutants. These experiments should quantitatively complement the results in Figure 5. KD of Prelid would be expected to increase mitochondrial elongation but there is no difference compared to WT in Figure 5.*"

Our Response to Reviewer #1 Comment #6. We thank the reviewer for this comment and now include new data to further demonstrate that co-treatment with the PERK kinase inhibitor GSK2656157 inhibits Tg-dependent increases in PA, while the PERK signaling inhibitor ISRIB does not (**Fig. 2B** and **Fig. S2A-E**). Further, it is published that *Perk*-deletion inhibits ER stress-induced increases in PA, while knockin cells expressing the non-phosphorylatable eIF2α S51A mutant do not (Bobrovnikova-Marjon et al (2012) *Mol Cell Biol*). This is consistent with a model whereby PERK-dependent increases in PA are attributed to a PERK kinase-dependent, yet eIF2α phosphorylation-independent, mechanism. In the revised manuscript, we include additional quantification of PA across other genetic manipulations, as requested. Notably, we confirm that Lipin1 overexpression reduced basal PA and prevents Tg-dependent increases in PA (**Fig. 2C**, **Fig. S2F,G**). Further, we show that *PRELID1* depletion does not significantly impact Tg-dependent increases of PA (**Fig. S5B**).

However, it is important to highlight that our work is *specifically* monitoring how acute ER stress-dependent PERK activation impacts mitochondria. Genetic manipulations that target many of the core components of these pathways are well established to globally disrupt many aspects of mitochondrial biology. Thus, these types of genetic manipulations often confound our ability to accurately monitor the contribution of specific stress-responsive signaling pathways in adapting mitochondria in response to acute insults. For example, a recent publication demonstrates that deletion of *Perk* impairs ER-mitochondrial phospholipid transport through mechanism independent of PERK kinase activity (Sassano et al (2023) *J Cell Biol*). While this problem can be limited if specific perturbations do not basally disrupt the phenotype being monitored (e.g., *PRELID1* depletion does not significantly impact basal mitochondrial morphology; **Fig. 5**), our ability to evaluate how stress-responsive signaling regulates mitochondria in response to acute insults (e.g., ER stress) still requires temporal control to properly evaluate how these pathways impact aspects of mitochondrial biology. It is for this reason that we paired *PRELID1* depletion with pharmacologic interventions that can be used to temporally inhibit PERK signaling (e.g., ISRIB, GSK), allowing us to best define the specific role for PERK-dependent reductions PRELID1 in promoting mitochondrial elongation in response to ER stress.

Reviewer #1 Comment #7. *“Title of the subsection: "hypomorphic PERK variants inhibit ER..." is inappropriate since authors only investigated a single hypomorphic variant (PSP). KO mutant is a null not hypomorphic mutant”*

Our Response to Reviewer #1 Comment #7. We agree and have made the suggested change in the revised manuscript.

Reviewer #1 Comment #8. “*Can the authors elaborate on the possible biological relevance for the inhibition of OPA1 cleavage via Tg treatment?*”

Our Response to Reviewer #1 Comment #8. We show that Tg pretreatment inhibits mitochondrial depolarization induced by CCCP (**Fig. S3G**). Thus, the impaired CCCP-induced, OMA1-dependent OPA1 processing observed in response to pretreatment with Tg likely reflects disruptions in mitochondrial uncoupling afforded by this treatment. We make this point clearer in the revised manuscript.

*Line 170: “However, Tg pretreatment inhibited CCCP-induced proteolytic cleavage of the inner membrane GTPase OPA1 (**Fig. 3C**) – a biological process upstream of DRP1 in mitochondrial fragmentation induced by membrane uncoupling.[43-47,64] This appears to result from Tg-dependent increases in mitochondrial membrane polarity (**Fig. S3G**), preventing efficient uncoupling in CCCP-treated cells and precluding our ability to determine whether Tg pretreatment directly impairs DRP1 activity under these conditions..”*

Reviewer #1 Comment #9. “*PRELID is a known short-lived protein; can the authors elaborate on possible additional impact due to 3-6 hr Tg treatment which is sufficient to induce expression of ATF4 target genes (Figure S2G).*”

Our Response to Reviewer #1 Comment #9. PRELID1 is a short-lived mitochondrial protein that is rapidly degraded in response to acute ER insults. As demonstrated in **Fig. 4** of our manuscript, this reduction is mediated by the IMM protease YME1L downstream of PERK-regulated translation attenuation. This 3-6 h timecourse corresponds with the translational attenuation induced downstream of PERK-dependent eIF2α phosphorylation following treatment with Tg and corresponds with the loss of PRELID1 observed in Tg-treated cells.

Note that the increase in ATF4 noted by the reviewer reflects the fact that ATF4 (and related proteins) are *preferentially* translated following eIF2α phosphorylation due to the presence of uORFs in their promoter. Thus, while global protein translation (including PRELID1 translation) is reduced by eIF2α phosphorylation, proteins like ATF4 are selectively translated.

Reviewer #1 Comment #10. "*Thapsigargin induced ER stress does not only activate PERK arm of the ISR, correct? Could the authors comment on this?*"

Our Response to Reviewer #1 Comment #10. I believe the reviewer is asking whether Tg treatment activates other arms of the integrated stress response (ISR). At the short timepoints used in this work (3-6 h), Tg-dependent increases in ISR signaling can be fully attributed to PERK signaling. This is evident as *Perk* deletion or inhibition blocks markers of ISR signaling in cells treated with Tg for these shorter timepoints (e.g., **Fig. 4E** of this paper; Harding et al (2002) *Mol Cell* and Lebeau et al (2018) *Cell Reports*). While other ISR kinases can be activated in response to more prolonged ER stress, the ISR activation observed in these shorter treatments with Tg are well established to be attributed to PERK activity.

Tg does induce all three arms of the unfolded protein response (i.e., ATF6, IRE1, and PERK) in the 3-6 h timeframe used in this manuscript. We previously showed that pharmacologic inhibition of ATF6 and IRE1 activity does not influence Tg-induced mitochondrial elongation (Lebeau et al (2018) *Cell Reports*). However, as reproduced in this manuscript, inhibition of PERK signaling blocks ER stress induced mitochondrial elongation. We make this point clearer in the revised manuscript.

Line 86. "*Pharmacologic inhibition of PERK signaling, but not other arms of the UPR, blocks mitochondrial elongation induced by ER stress.*[39]"

Reviewer #1 Comment #11. "*Addition of drugs and duration (3-6 hrs) likely very toxic to cells; how does this treatment affect viability? Unhealthy cells will have unhealthy mitochondria so it's hard to be confident that subtle morphological differences are specific. Why do authors use 3 hrs Tg-treatment after initially using 6 hrs in Figure 1? Would be helpful to assay toxicity and mitochondrial morphology of thapsigargin and other drugs in WT vs. Perk KO MEFs over time."*

Our Response to Reviewer #1 Comment #11. Thapsigargin (Tg) is toxic to cells, but apoptosis is observed in cell culture models only after much longer treatments 24-72 h. We are using Tg to monitor how cells respond to acute ER stress. We chose the short 3-6 h timecourse because this is sufficient to induce PERK-dependent translation attenuation independent of cell death. Consistent with this, we observe no reductions in cellular viability or death in the short 3-6 h treatments used in this study. This timecourse is standard in the field when monitoring cellular changes induced by acute ER stress.

Reviewer #1 Comment #12. "*Previously, an increase in fragmentation was observed at 0.5 hours but this subsided by 6 hours in WT (Lebeau J, et al. 2018) but is this the same for Perk KO MEFs?*"

Our Response to Reviewer #1 Comment #12. The increase in mitochondrial fragmentation observed following Tg treatment results from the rapid increase of mitochondrial Ca^{2+} induced by this treatment (Hom et al (2007) *J Cell Phy*). Consistent with this, we have found that pharmacologic inhibition of PERK signaling using the compound ISRIB, does not inhibit mitochondrial fragmentation in MEF^{mtGFP} cells treated for 30 min with Tg. Since *Perk*-deficient MEFs already show increased fragmentation (**Fig. 1B,C**), monitoring mitochondrial morphology in *Perk*-deficient cells treated with Tg for 30 min is unlikely to reveal additional insights into the mechanism outlined in this manuscript.

Reviewer #1 Comment #13. "*How much protein was loaded per lane and what was the percentage of polyacrylamide gel? Please clarify details in methodology*."

Our Response to Reviewer #1 Comment #13. We loaded 100 µg of protein for our immunoblotting experiments. We used 10% or 12% SDS-PAGE gels. We included this information in the revised *Materials and Methods.*

Reviewer #1 Comment #14. "*Figure 1A is virtually identical to Figure 2A (with exception of "MEF A/A") from previous publication: Lebeau J, Saunders JM, Moraes VWR, Madhavan A, Madrazo N, Anthony MC, Wiseman RL. The PERK Arm of the Unfolded Protein Response Regulates Mitochondrial Morphology during Acute Endoplasmic Reticulum Stress. Cell Rep. 2018 Mar 13;22(11):2827-2836. doi: 10.1016/j.celrep.2018.02.055. PMID: 29539413; PMCID: PMC5870888.*"

Our Response to Reviewer #1 Comment #14. Yes. **Fig. 1A** is a cartoon showing PERK-dependent regulation of mitochondria and the specific pharmacologic and genetic manipulations used in this paper to alter this pathway. This is adapted from our previous manuscript (Lebeau et al (2018) *Cell Reports*). We properly reference this adaptation in the revised manuscript. We feel it is important to show this figure to specifically highlight how different manipulations influence this signaling pathway.

Reviewer #1 Comment #15. "*If the authors' hypothesis is correct, overexpression of PRELID1 should have same effect as overexpression of Lipin*"

Our Response to Reviewer #1 Comment #15. Overexpressed PRELID1 will be sensitive to the same rapid YME1L-dependent degradation observed for the endogenous protein. Thus, overexpressing PRELID1 would be expected to have no effect (or a very minor effect) on mitochondrial morphology in Tg-treated cells. We show that Lipin1 overexpression basally increases mitochondrial fragmentation and blocks Tg-induced mitochondrial elongation (**Fig. 2**). Identical results were observed in cells overexpressing the alternative PA lipase PA-PLA1 (**Fig.**

S2). We feel that these data, in combination with others shown in our manuscript, strongly support the dependence of this process on PA levels and localization.

Reviewer #1 Comment #16. “*What is the selective marker used for HeLa cells expressing mitoPLDGFP since the HeLa parental cell background already expressed a mitochondrial targeted GFP, we assume it was puromycin but this was not clear in the Figure legend or methods? If so, it would be helpful to clarify this. If not, how can the authors observe a difference in morphology if the selectable marker is the same? Indeed, mitoPLDGFP is expressed, detectable by immunoblot, but this is on a cell population level so no way of knowing whether the specific cells scored expressed mitoPLDGFP unless another selectable marker was used (i.e. should have used CFP, RFP, etc.).”*

“*The authors state "Note the expression of mitoPLDGFP did not impair our ability to accurately monitor mitochondrial morphology in these cells." in Figure 3 legend and again basically the same in S3: "Note that the expression of the mitoPLDGFP did not impair our ability to monitor mitochondrial morphology in these cells." Could the authors explain their reasoning here?*”

Our Response to Reviewer #1 Comment #16. We co-transfected the mitochondrial localized mitoPLD-GFP with ^{mt}GFP in HeLa cells using calcium phosphate transfection. In using this approach, we (and others) have consistently found that this method leads to the efficient transfection of cells with both plasmids. Thus, cells will express both mitoPLD-GFP and ^{mt}GFP. We used mitoPLD-GFP because we were reproducing published experiments (Adachi et al (2016) *Mol Cell*) and we wanted to use the same overexpression plasmid used in these previous studies. It is clear from our images that the presence of GFP-tagged mitoPLD did not influence our ability to accurately monitor mitochondrial elongation in these cells. Further, the robust increase in mitochondrial elongation observed in cells overexpressing mitoPLD-GFP and the further increase in elongation observed upon co-treatment with Tg demonstrate the effectiveness of this assay. This is consistent with published results (Adachi et al (2016) *Mol Cell*).

Reviewer #1 Comment #17. **“***Figure S4C: the authors show that Tg treatment on MEF mtGFP cells for distinct hours to determine PRELID levels. However, in the Results section states that this treatment was with CHX, could the authors please check this and correct?”*

Our Response to Reviewer #1 Comment #17. The data shown in **Fig. S4C** from the previous version is in Tg-treated cells. We corrected this in the revised manuscript.

Reviewer #1 Comment #18. “*Figure 6: A schematic representation should be a graphic summary of all findings reported in the text with no text except where absolutely essential. A good model should be easily understood without reading any description since all concepts were supported in the main text and by experimentation.”*

"The model also contains some inaccuracies. The suggestion is that the authors re-do the model and clarify some aspects such as:
The model suggests that ISRIB inhibits PRELID1 directly but there is no evidence for this whereas PRELID is directly regulated by YME1L (also typo here in figure: "Yme1" no "l").
This model incorrectly uses inhibition symbols; for example, mutation of Perk does not inhibit its activity as GSK does. The KO does not have Perk so cannot perform its function. These are not the same.
Similarly, the lipases (Lipin and PA-PLA1) should be depicted instead as altering flux of PA away from OMM not as inhibition.
The authors should connect PA accumulation in the OMM graphically to mitochondrial elongation [instead of through text]. If the authors consider the numbered labels convenient, please use just the number and place the description in the figure legend instead."

Our Response to Reviewer #1 Comment #18. We have adapted our model shown in **Fig. 6** and the accompanying legend to address points brought up by the reviewer. In particular, because the reviewer found it difficult to follow how specific manipulations impacted specific steps, we removed those parts from the revised figure for clarity.

Reviewer #1 Comment #19. The reviewer made many suggestions to improve the *Materials and Methods* section of this manuscript in their review, which we do not include here for space considerations.

Our Response to Reviewer #1 Comment #19. We have addressed all of the reviewer's comments regarding the *Materials and Methods* section in our revised manuscript.

Reviewer #1 Comment #20. The reviewer made many suggestions about the presentation of our figures that we do not include here for space considerations.

Our Response to Reviewer #1 Comment #20. We have addressed all of the reviewer's comments regarding the ***Figures*** in our revised manuscript.

REVIEWER #2.

Reviewer #2 General Comments. "*Previous studies have shown that ER stress increases amounts of phosphatidic acid (PA) (PMID: 22493067) and induces elongation of mitochondria through the protein and lipid kinase PERK (PMID: 29539413, work by Wiseman's lab). The current work reports that ER stress by thapsigargin promotes the degradation of a mitochondrial protein PRELID1, which transfers PA from the outer membrane to the inner membrane. An inner membrane protease, YME1L, was identified as responsible for this degradation of PRELID1.*

Consistent with the notion that PA is required for the morphological change, overexpression of a PA phosphatase (Lipin) or a PA phospholipase (PA-PLA1) decreased ER-stress-induced mitochondrial elongation."

"Overall, this manuscript is a nice extension of the authors' previous work and investigates the molecular mechanism underlying the regulation of mitochondrial elongation induced by ER stress. However, the current data do not strongly support the role of PRELID1 in either ER-stress-mediated PA level elevation or mitochondrial elongation, as described in Specific comments. The authors should address these points."

Our Response to Reviewer #2 General Comments. We thank the reviewer for the thorough and careful read of our manuscript. We address the specific points brought up by the reviewer in our revised manuscript, as described below.

Reviewer #2 Comment #1. "*The authors report that PRELID1 knockdown did not promote mitochondrial elongation under either normal or ER-stress conditions (Fig. 5). If PRELID1 plays a vital role in mitochondrial elongation, PRELID1 depletion will restore elongation. Therefore, the presented data argue against the authors' conclusion. Since PRELID1 has multiple homologs, including PRELID3B, which is also a short-lived protein like PRELID1, these homologs might redundantly function in PA transport, especially when PRELID1 is absent. Therefore, the authors need to knock them down simultaneously. This possibility is consistent with the previous authors' data that YME1L depletion decreases ER-stress-induced mitochondrial elongation (PMID: 29539413). YME1L knockdown may rescue multiple short-lived PRELID1 homologs."*

Our Response to Reviewer #2 Comment #1. Our model indicates that ER stress-dependent increases in mitochondrial elongation require two steps: 1) PERK kinase-dependent increases in total PA and 2) YME1L-dependent degradation of PRELID1 downstream of PERK-dependent translation attenuation. Thus, it is not surprising that *PRELID1* depletion did not induce mitochondrial elongation on its own. However, we do demonstrate that depletion of *PRELID1* rescues Tg-induced mitochondrial elongation in cells co-treated with the PERK signaling inhibitor ISRIB – a compound that specifically blocks Tg-dependent PRELID1 degradation, but not PERK kinase dependent increases in total PA (**Fig. 6**). This demonstrates that PRELID1 reductions are *required*, but not *sufficient* for promoting mitochondrial elongation. We make this point more clear in the revised manuscript.

*Line 259: "Interestingly, Prelid1 depletion did not basally influence mitochondrial morphology or inhibit Tg-induced mitochondrial elongation (**Fig. 5A,B** and **Fig. S5E**). This indicates that reduction of PRELID1, on its own, is not sufficient to increase mitochondrial elongation, likely reflecting the importance of PERK kinase-dependent increases in PA in this process.[53]"*

With respect to PRELID3B/SLMO2. This lipid transporter is primarily associated with trafficking phosphatidylserine (PS) from the outer to the inner mitochondrial membrane, where it is then

converted to phosphatidylethanolamine (PE). As alluded to by the reviewer, we found that SLMO2, like PRELID1, is also a short-lived mitochondrial protein that is rapidly degraded by YME1L downstream of PERK-dependent translation attenuation. We have also found that Tg treatment disrupts mitochondrial PE levels through a PERK-dependent mechanism on a similar timescale to that observed for PA changes. However, shRNA depletion of *SLMO2* in HeLa cells – a condition that mimics the reductions in SLMO2 observed during ER stress – *increases* basal mitochondrial fragmentation and inhibits Tg-induced mitochondrial elongation. Since chronic, genetic reductions in SLMO2 (which mirror the acute reduction in SLMO2 observed during ER stress) show opposite impacts on mitochondrial morphology to that observed upon Tg treatment, we interpreted this result to indicate that SLMO2 reductions are likely not involved in PERK-dependent regulation of mitochondrial elongation during acute ER stress. In contrast, depletion of *PRELID1* is sufficient to rescue Tg-induced mitochondrial elongation in cells co-treated with ISRIB (**Fig. 5A-D**) – a compound that selectively blocks ER stress-dependent reductions in PRELID1. This implicates reductions in PRELID1 in this process. We are continuing to define the specific impact of PERK-dependent regulation of SLMO2 on mitochondrial morphology, ultrastructure, and/or function in work outside the scope of this current manuscript, but we felt it most appropriate to focus this manuscript on PA-dependent morphology remodeling based on the presented data.

Reviewer #2 Comment #2. *"Another possibility is that since a previous study has shown that PERK-produced PA activates the mTOR-AKT pathway (PMID: 22493067), this signaling pathway may contribute to mitochondrial morphology in addition to PRELID1. The authors should test the combined effects of mTOR-AKT inhibition in ER-stress-induced mitochondrial elongation."*

Our Response to Reviewer #2 Comment #2. As highlighted by the reviewer, PERK-dependent increases in PA can influence mTOR and AKT activity. To test this, we monitored mTOR-dependent S6K phosphorylation and AKT phosphorylation in MEF^{mtGFP} and HeLa cells treated with Tg for 3 h. While we did observe increases in S6K phosphorylation in Tg-treated MEF^{mtGFP} cells, mTOR activity was not changed in Tg-treated HeLa cells. AKT phosphorylation was not affected in MEF^{mtGFP} or HeLa cells (not shown). We include these mTOR data in the revised manuscript (see **Fig. S3C,D**). Since we observe PERK-dependent mitochondrial elongation in both MEF^{mtGFP} and HeLa cells, we interpret these results to indicate that PA-dependent increases in mTOR activity is not primarily responsible for ER stress dependent increases in mitochondrial elongation across cell types. We describe these results in the revised manuscript.

*Line 156: "In contrast, PERK-dependent increases in PA can activate mTOR during ER stress.[53] Consistent with this, we observe Tg-dependent increases in mTOR-dependent S6K phosphorylation in MEF^{mtGFP} cells (**Fig. S3C**). However, despite increasing PA and promoting mitochondrial elongation, Tg did not increase S6K phosphorylation in HeLa cells (**Fig. S3D**). These results suggest that PERK-dependent alterations in mTOR activity are unlikely to be primary contributors to ER stress induced mitochondrial elongation across cell types."*

Reviewer #2 Comment #3. "*The authors' model suggests the loss of PRELID1 increases PA levels in the mitochondrial outer membrane (Fig. 6). The authors should test PA levels in mitochondria isolated from cells depleted for PRELID1 and its homologs (simultaneously). Since PA that is transported to the inner membrane is actively converted to other phospholipids, such as CDP-DAG, elevated levels of PA are likely seen if the outer membrane to inner membrane transport is blocked.*

Our Response to Reviewer #2 Comment #3. We agree with the reviewer it is important to evaluate how PERK-dependent degradation of PRELID1 impacts other phospholipids dependent on PA trafficking to the IM where it can be converted to other lipids, most notably cardiolipin (CL). In the revised manuscript, we now show measurements of CL in MEF^{mtGFP} cells treated with Tg and/or depleted of *Prelid1*. As expected, reductions in PRELID1 decrease CL in isolated mitochondria (**Fig. S5C**). Treatment with Tg reduced CL to similar extents in MEF^{mtGFP}-treated cells expressing non-silencing shRNA. However, we did not observe further reductions of CL in *Prelid1*-depleted cells. This is consistent with a model whereby ER stress-dependent reductions in PRELID1 decrease PA trafficking across the IMS and lead to reductions in CL synthesis. These results are discussed in the revised manuscript as below:

*Line 214. "PRELID1 traffics PA from the outer to inner mitochondrial membrane, where it serves as a precursor to the formation of cardiolipin.[56,66,67] Thus, reductions in PRELID1 should decrease cardiolipin. To test this, we shRNA-depleted Prelid1 from MEF^{mGFP} cells and monitored cardiolipin in isolated mitochondria in the presence or absence of ER stress. We confirmed efficient PRELID1 knockdown by immunoblotting (**Fig. S5A**). Importantly, Prelid1 depletion did not alter Tg-induced reductions of TIM17A or increases of ATF4. Further, Tg-dependent increases in PA were observed in Prelid1-depleted MEF^{mtGFP} cells (**Fig. S5B**). These results indicate that loss of PRELID1 does not impair PERK signaling in these cells. Prelid1 depletion reduced cardiolipin in mitochondria isolated from MEF^{mtGFP} cells (**Fig. S5C**). Treatment of MEF^{mtGFP} cells expressing non-silencing shRNA with Tg for 3 h also reduced cardiolipin to levels similar to those observed in Prelid1-deficient cells. However, Tg did not further reduce cardiolipin in Prelid-depleted cells. These results are consistent with a model whereby ER stress-dependent reductions in PRELID1 limit PA trafficking across the inner mitochondrial membrane and contribute to reductions in cardiolipin during acute ER stress."*

We are continuing to define how PERK signaling influences other mitochondrial phospholipids during conditions of ER stress in work outside the scope of this manuscript. Notably, we are continuing to evaluate how ER stress and PERK signaling influences aspects of cardiolipin synthesis in response to both acute and chronic ER stress. Further, as discussed above, we are determining how PERK-dependent reductions in PRELID3B/SLMO2 influence PS trafficking and subsequent PE synthesis at the IM and the implications of these changes on mitochondrial biology. Initial experiments indicate that PERK signaling reduces PE during ER stress, indicating that other phospholipids can be influenced by this pathway. However, we view this work as being

outside the scope of the current manuscript focused specifically on defining the impact of PA remodeling on mitochondrial morphology.

Reviewer #2 Comment #4. ***"****The authors need to test whether Lipin and PA-PLA1 overexpression decreased PA levels in mitochondria treated with thapsigargin. The current manuscript only shows the effect of Lipin and PA-PLA1 on PA levels in whole-cell lysate without ER stress (Fig. S2F)."*

Our Response to Reviewer #2 Comment #4. We agree. In the revised manuscript, we now show that Lipin overexpression blocks Tg-dependent increases in PA (**Fig. 2C**). Identical experiments are also shown for *Prelid1*-depleted cells (**Fig. S5B**).

Reviewer #2 Comment #5. "*The authors propose that PA inhibits DRP1 in mitochondrial division under ER stress. It has been shown that PA blocks DRP1 after recruitment to mitochondria (PMID: 27635761). Does thapsigargin induce mitochondrial accumulation of DRP1?"*

Our Response to Reviewer #2 Comment #5. The reviewer is correct that our results suggest that ER stress promotes mitochondrial elongation through a model involving PA-dependent inhibition of mitochondrial fission at the outer membrane. In the revised manuscript, we now show that Tg treatment does not significantly influence the recovery of DRP1 in mitochondrial fractions (**Fig. S3A**). Further, we recapitulate results from previous publications showing that Tg does not significantly influence DRP1 phosphorylation at either S637 or S616 (**Fig. S3B**). This indicates that DRP1 localization and posttranslational modification does not appear affected by Tg treatment. However, we do show that Tg pretreatment inhibits DRP1-dependent mitochondrial fission induced by ionomycin (**Fig. 3D,E**). Combined with other results, our data are consistent with a model whereby PERK-dependent increases in PA *and* PRELID1 degradation leads to the accumulation of PA on the OM where it can inhibit DRP1 activity (**Fig. 6**). We make this point clearer in the revised manuscript.

*Line 154: "However, as reported previously[39], Tg did not influence DRP1 phosphorylation at either S637 or S616 (**Fig. S3A**) or alter the amount of DRP1 enriched in mitochondrial fractions from MEF^{mtGFP} cells (**Fig. S3B**)."*

REVIEWER #3.

Reviewer #3 General Comments. "*The authors investigated signaling pathways and molecular mechanisms leading to mitochondrial dysfunction after ER stress. This study extends their previous publication (Lebeau et al., 2018) by providing evidence on how PERK regulates mitochondrial structure and function in response to ER stress. Some key findings are that PERK induces mitochondrial elongation by increasing and retaining phosphatidic acid (PA) in the outer*

mitochondrial membrane which is important for cell adaptation and survival. This process requires PERK-dependent translational attenuation through YME1L-PRELID dependent mechanism. This is a very strong study with compelling evidence."

"*This study adds to our current knowledge on how ER stress affects mitochondria adaptation and proteostasis, which may contribute to the pathogenesis and progression of numerous neurodegenerative diseases. Specifically, this study establishes a new role for PERK in mitochondrial adaptive remodeling focused on trafficking and accumulation of phospholipids. Identifying molecular markers like PERK and its involvement with PRELID, YME1L, and PA to regulate mitochondrial remodeling during ER stress is important to understand the effects of drug-targeting this ER stress-responsive factor."*

Our Response to Reviewer #3 General Comments. We thank the reviewer for the enthusiastic comments about our manuscript. We address the reviewers remaining concerns as outlined below.

Reviewer #3 Comment #1. "*Only one minor point should be addressed: In Fig S2G & H, the authors indicate that "Lipin1 overexpression did not significantly influence increases of ATF4 protein". The blots show a decrease in ATF4 in Tg-treated HeLa cells. The same effect is observed in Fig. S3F showing reduction in ATF4, but the authors described it as the "overexpression of mitoPLD did not significantly impact other aspects of PERK signaling in Tg-treated cells". The quantification of the blots or indication that the blots were quantified should be clarified and noted (at least in the legend)."*

Our Response to Reviewer #3 Comment #1. We agree. We now include quantification of ATF4 in immunoblots from HeLA cells overexpressing lipin1 and treated with Tg (**Fig. S2J**). As we suggested, these results confirm that Tg treatment does not significantly influence ATF4 expression in these cells. In addition, we now include additional data showing that lipin overexpression does not significantly reduce Tg-dependent expression of ISR target genes including *Asns* or *Chop* (**Fig. S2I**). This further supports other findings in the manuscript showing that different manipulations do not significantly impact ISR signaling (evident by ATF4 expression or TIM17A or PRELID1 degradation).

1st Editorial Decision 3rd May 2023

Dear Luke,

We have now received an adjudicating report from an external expert, which I have attached to the bottom of this letter. As you will see, the feedback was clearly supportive of publication. Before I can finally accept the manuscript though, there are some remaining editorial points which need to be addressed. In this regard would you please:

- make sure that the final text file (.doc) contains no figures and no track changes,
- pay attention to the comments in the data edited manuscript file (on sample size, defining the nature of replicates, defining error bars),
- ensure that the references are in alphabetical order; in the text, please use 1 author + et al. for references with long author lists, please limit to 10 authors + et al. in the reference section,
- rename the conflict of interest statement as the "DISCLOSURE AND COMPETING INTERESTS STATEMENT",
- remove the AC/CrediT section from the text,
- include figures as separate files, with additional figures uploaded individually as EV figures, and
- reorganize Appendix figure Source data file to one file/folder per figure and zip.

The publication of source data, particularly for electrophoretic gels and blots, makes primary data more accessible and transparent to the reader. Please provide me with a PDF file per figure that contains the original, uncropped and unprocessed scans of all or key gels used in the figures. The PDF files should be labeled with the appropriate figure/panel number, and should have molecular weight markers; further annotation could be useful but is not essential. The PDF files will be published online with the article as supplementary "Source Data" files. Source Data can also include Excel tables to accompany your graphs. We anticipate that their inclusion will make your work more discoverable and useable to scientists in the future.

We include a synopsis of the paper (see http://emboj.embopress.org/). Please provide me with a general summary statement and 3-5 bullet points that capture the key findings of the paper.
EMBO Press is an editorially independent publishing platform for the development of EMBO scientific publications.

Best wishes,

William

William Teale, PhD
Editor
The EMBO Journal
w.teale@embojournal.org

Instructions for preparing your revised manuscript:

Please check that the title and abstract of the manuscript are brief, yet explicit, even to non-specialists.

When assembling figures, please refer to our figure preparation guideline in order to ensure proper formatting and readability in print as well as on screen:
https://bit.ly/EMBOPressFigurePreparationGuideline
See also figure legend guidelines: https://www.embopress.org/page/journal/14602075/authorguide#figureformat

IMPORTANT: When you send the revision we will require
- a point-by-point response to the referees' comments, with a detailed description of the changes made (as a word file).
- a word file of the manuscript text.
- individual production quality figure files (one file per figure)
- a complete author checklist, which you can download from our author guidelines (https://www.embopress.org/page/journal/14602075/authorguide).
- Expanded View files (replacing Supplementary Information)
Please see out instructions to authors
https://www.embopress.org/page/journal/14602075/authorguide#expandedview

Please remember: Digital image enhancement is acceptable practice, as long as it accurately represents the original data and

conforms to community standards. If a figure has been subjected to significant electronic manipulation, this must be noted in the figure legend or in the 'Materials and Methods' section. The editors reserve the right to request original versions of figures and the original images that were used to assemble the figure.

Further information is available in our Guide For Authors: https://www.embopress.org/page/journal/14602075/authorguide

We realize that it is difficult to revise to a specific deadline. In the interest of protecting the conceptual advance provided by the work, we recommend a revision within 3 months (1st Aug 2023). Please discuss the revision progress ahead of this time with the editor if you require more time to complete the revisions. Use the link below to submit your revision:

https://emboj.msubmit.net/cgi-bin/main.plex

Adjudicating referee

I have checked the manuscript and the point-by-point response by Luke Wiseman and colleagues.

They performed a very careful and detailed revision of the manuscript and addressed all relevant comments of the reviewers in a clear and constructive manner. Particularly, they included substantial new experimental results and revised the text according to the suggestions of the reviewers.

The paper reveals a very interesting mechanism of how endoplasmic reticulum stress affects mitochondrial phospholipid distribution and fission/fusion. The authors identify the relevant players and characterize them by careful experiments. The functional cross-talk between ER and mitochondria under stress conditions is of broad interest to cell biologists, biochemists and molecular biologists and, thus, the elucidation of responsible components and molecular mechanisms is of great value to the readership of the EMBO Journal.

I recommend publication of this innovative study in the EMBO Journal.

RESPONSE TO EDITOR

We thank you and the external examiner for their positive response to our revised submission. We address all of the editorial points and formatting requests in this revised submission. We address these changes to the text below:

EDITORIAL COMMENTS.

Editorial Comment #1. "*make sure that the final text file (.doc) contains no figures and no track changes"*

Our Response to Editorial Comment #1. We have submitted a .docx file that contains no figures and shows no changes tracked.

Editorial Comment #2. "*pay attention to the comments in the data edited manuscript file (on sample size, defining the nature of replicates, defining error bars)"*

Our Response to Editorial Comment #2. All data presented, except those shown in **Fig. S2I**, are from biological replicates isolated separately or independent experiments. We make this point clear in the revised figure legends. The error bars in **Fig. S2I** show 95% confidence interval for n=3 technical replicates. This is now clear in the **Fig. S2I** figure legend included in the revised submission.

Editorial Comment #3. "*ensure that the references are in alphabetical order; in the text, please use 1 author + et al. for references with long author lists, please limit to 10 authors + et al. in the reference section"*

Our Response to Editorial Comment #3. We have changed the reference formatting according to EMBO guidelines using the EMBO style in Endnote.

Editorial Comment #4. *"rename the conflict of interest statement as the "DISCLOSURE AND COMPETING INTERESTS STATEMENT"*

Our Response to Editorial Comment #4. We have made this change in the revised submission.

Editorial Comment #5. *"remove the AC/CrediT section from the text"*

Our Response to Editorial Comment #5. This section is removed in the revised manuscript.

Editorial Comment #6. *"include figures as separate files, with additional figures uploaded individually as EV figures"*

Our Response to Editorial Comment #6. We have uploaded individual main text and extended view figures as PNGs in the revised submission.

Editorial Comment #7. "*reorganize Appendix figure Source data file to one file/folder per figure and zip."*

Our Response to Editorial Comment #7. We included a zip file including individual PDFs showing whole gels for all immunoblots included in this manuscript.

Editorial Comment #8. "*Please provide me with a general summary statement and 3-5 bullet points that capture the key findings of the paper"*

Our Response to Editorial Comment #8. We include a general summary statement and 3-5 bullet points. This document can be found in the "*Editorial Summary"* document included in the revised submission.

Dear Luke,

I am pleased to inform you that your manuscript has been accepted for publication in the EMBO Journal.

Thank you for choosing EMBO Journal for this piece of work - we'll be really proud to publish it!

Please note that it is EMBO Journal policy for the transcript of the editorial process (containing referee reports and your response letter) to be published as an online supplement to each paper. If you do NOT want this, you will need to inform the Editorial Office via email immediately. More information is available here:
https://www.embopress.org/page/journal/14602075/authorguide#transparentprocess

Your manuscript will be processed for publication in the journal by EMBO Press. Manuscripts in the PDF and electronic editions of The EMBO Journal will be copy edited, and you will be provided with page proofs prior to publication. Please note that supplementary information is not included in the proofs.

You will be contacted by Wiley Author Services to complete licensing and payment information. The required 'Page Charges Authorization Form' is available here: https://www.embopress.org/pb-assets/embo-site/tej_apc.pdf - please download and complete the form and return to embopressproduction@wiley.com

EMBO Press participates in many Publish and Read agreements that allow authors to publish Open Access with reduced/no publication charges. Check your eligibility: https://authorservices.wiley.com/author-resources/Journal-Authors/open-access/affiliation-policies-payments/index.html

Should you be planning a Press Release on your article, please get in contact with embojournal@wiley.com as early as possible, in order to coordinate publication and release dates.

If you have any questions, please do not hesitate to call or email the Editorial Office. Thank you for your contribution to The EMBO Journal.

Best wishes,

William

William Teale, PhD
Editor
The EMBO Journal
w.teale@embojournal.org

** Click here to be directed to your login page: https://emboj.msubmit.net

EMBO Press Author Checklist

Corresponding Author Name: R. Luke Wiseman	
Journal Submitted to: EMBO Journal	
Manuscript Number: EMBOJ-2023-113908	

USEFUL LINKS FOR COMPLETING THIS FORM
The EMBO Journal - Author Guidelines
EMBO Reports - Author Guidelines
Molecular Systems Biology - Author Guidelines
EMBO Molecular Medicine - Author Guidelines

Reporting Checklist for Life Science Articles (updated January
This checklist is adapted from Materials Design Analysis Reporting (MDAR) Checklist for Authors. MDAR establishes a minimum set of requirements in transparent reporting in the life sciences (see Statement of Task: 10.31222/osf.io/9sm4x). Please follow the journal's guidelines in preparing your manuscript.
Please note that a copy of this checklist will be published alongside your article.

Abridged guidelines for figures
1. Data
The data shown in figures should satisfy the following conditions:
- the data were obtained and processed according to the field's best practice and are presented to reflect the results of the experiments in an accurate and unbiased manner.
- ideally, figure panels should include only measurements that are directly comparable to each other and obtained with the same assay.
- plots include clearly labeled error bars for independent experiments and sample sizes. Unless justified, error bars should not be shown for technical
- if n<5, the individual data points from each experiment should be plotted. Any statistical test employed should be justified.
- Source Data should be included to report the data underlying figures according to the guidelines set out in the authorship guidelines on Data

2. Captions
Each figure caption should contain the following information, for each panel where they are relevant:
- a specification of the experimental system investigated (eg cell line, species name).
- the assay(s) and method(s) used to carry out the reported observations and measurements.
- an explicit mention of the biological and chemical entity(ies) that are being measured.
- an explicit mention of the biological and chemical entity(ies) that are altered/varied/perturbed in a controlled manner.
- the exact sample size (n) for each experimental group/condition, given as a number, not a range;
- a description of the sample collection allowing the reader to understand whether the samples represent technical or biological replicates (including how many animals, litters, cultures, etc.).
- a statement of how many times the experiment shown was independently replicated in the laboratory.
- definitions of statistical methods and measures:
  - common tests, such as t-test (please specify whether paired vs. unpaired), simple χ2 tests, Wilcoxon and Mann-Whitney tests, can be unambiguously identified by name only, but more complex techniques should be described in the methods section;
  - are tests one-sided or two-sided?
  - are there adjustments for multiple comparisons?
  - exact statistical test results, e.g., P values = x but not P values < x;
  - definition of 'center values' as median or average;
  - definition of error bars as s.d. or s.e.m.

Please complete ALL of the questions below.
Select "Not Applicable" only when the requested information is not relevant for your study.

Materials

Newly Created Materials	Information included in the manuscript?	In which section is the information available? (Reagents and Tools Table, Materials and Methods, Figures, Data Availability Section)
New materials and reagents need to be available; do any restrictions apply?	Not Applicable	

Antibodies	Information included in the manuscript?	In which section is the information available? (Reagents and Tools Table, Materials and Methods, Figures, Data Availability Section)
For **antibodies** provide the following information:<br>- Commercial antibodies: RRID (if possible) or supplier name, catalogue number and or/clone number<br>- Non-commercial: RRID or citation	Yes	Antibody information is included in the Materials and Methods and copied here: TIM17A (Thermo Scientific, PA5-21925; 1:1000), PRELID1 [aa27-54] (LS Bio, LC-C158729; 1:1000), YME1L (Proteintech, 11510-1-AP; 1:1000), ATF4 (Cell Signaling, 11815; 1:500), Tubulin [B-5-1-2] (Sigma, T6074; 1:5000), TIM23 (BD Transduction Labs, 611222; 1:1000), HSP60 [LK1] (Thermo Scientific, MA1-35434; 1:1000), PERK (Cell Signaling, 3192S; 1:1000), HA [Clone: 16B12] (Biolegend, 901501 1: 1000), GFP (B2) (Santa Crux, sc9996; 1:1000), OPA1 (BD Biosciences, 612606; 1:2000), Phospho-DRP1 (Ser616) (ThermoFisher, PA5-64821; 1:1000), Phospho-DNM1L (Ser637) (ThermoFisher, PA5; 101038; 1:1000), DNM1L (ThermoFisher, MA5-26255; 1:1000), p70 S6 Kinase p(Ser389) (Cell Signaling, 9206S; 1:1000), p70 S6 Kinase (Cell Signaling, 9202S; 1:1000)

DNA and RNA sequences	Information included in the manuscript?	In which section is the information available? (Reagents and Tools Table, Materials and Methods, Figures, Data Availability Section)
**Short novel DNA or RNA including primers, probes**: provide the sequences.	Yes	Primers used for qPCR in this manuscript are described in the Materials and Methods and copied here: Human ASNS: forward: GCAGCTGAAAGAAGCCCAAG; reverse: AGCCTGAATGCCTTCCTCAG Human CHOP/DDIT3: forward: ACCAAGGGAGAACCAGGAAACG; reverse TCACCATTCGGTCAATCAGAGC Human HSPA5/BIP: forward: GCCTGTATTTCTAGACCTGCC; reverse, TTCATCTTGCCAGCCAGTTG Human RIBOP: forward: CGT CGC CTC CTA CCT GCT; reverse, CCA TTC AGC TCA CTG ATA ACC TTG

Cell materials	Information included in the manuscript?	In which section is the information available? (Reagents and Tools Table, Materials and Methods, Figures, Data Availability Section)
**Cell lines:** Provide species information, strain. Provide accession number in repository **OR** supplier name, catalog number, clone number, and/**OR** RRID.	Yes	The cells used in this study were purchased from the ATCC or provided by the indicated researcher. Appropriate references are included. These are described in the materials and methods and again here: MEFmtGFP (a kind gift from Peter Schultz, TSRI)85, Perk+/+ and Perk-/- MEFs25, Atf4+/+ and Atf4-/- MEFs70 (kind gifts from David Ron, Cambridge), HeLa cells (purchased from the ATCC), or HEK293T cells
**Primary cultures:** Provide species, strain, sex of origin, genetic modification status.	Not Applicable	
Report if the cell lines were recently **authenticated** (e.g., by STR profiling) and tested for mycoplasma contamination.	Yes	We perform mycoplasma testing on our cells lines every ~6 months.

Experimental animals	Information included in the manuscript?	In which section is the information available? (Reagents and Tools Table, Materials and Methods, Figures, Data Availability Section)
**Laboratory animals or Model organisms:** Provide species, strain, sex, age, genetic modification status. Provide accession number in repository **OR** supplier name, catalog number, clone number, **OR** RRID.	Not Applicable	
**Animal observed in or captured from the field:** Provide species, sex, and age where possible.	Not Applicable	
Please detail **housing and husbandry conditions**.	Not Applicable	

Plants and microbes	Information included in the manuscript?	In which section is the information available? (Reagents and Tools Table, Materials and Methods, Figures, Data Availability Section)
**Plants:** provide species and strain, ecotype and cultivar where relevant, unique accession number if available, and source (including location for collected wild specimens).	Not Applicable	
**Microbes:** provide species and strain, unique accession number if available, and source.	Not Applicable	

Human research participants	Information included in the manuscript?	In which section is the information available? (Reagents and Tools Table, Materials and Methods, Figures, Data Availability Section)

If collected and within the bounds of privacy constraints report on age, sex and gender or ethnicity for all study participants.	Not Applicable	

**Core facilities**	**Information included in the manuscript?**	**In which section is the information available?** (Reagents and Tools Table, Materials and Methods, Figures, Data Availability Section)
If your work benefited from core facilities, was their service mentioned in the acknowledgments section?	Not Applicable	

Design

**Study protocol**	**Information included in the manuscript?**	**In which section is the information available?** (Reagents and Tools Table, Materials and Methods, Figures, Data Availability Section)
If study protocol has been **pre-registered, provide DOI in the manuscript**. For clinical trials, provide the trial registration number **OR** cite DOI.	Not Applicable	
Report the **clinical trial registration number** (at ClinicalTrials.gov or equivalent), where applicable.	Not Applicable	

**Laboratory protocol**	**Information included in the manuscript?**	**In which section is the information available?** (Reagents and Tools Table, Materials and Methods, Figures, Data Availability Section)
Provide DOI OR other citation details if **external detailed step-by-step protocols** are available.	Not Applicable	

**Experimental study design and statistics**	**Information included in the manuscript?**	**In which section is the information available?** (Reagents and Tools Table, Materials and Methods, Figures, Data Availability Section)
Include a statement about **sample size** estimate even if no statistical methods were used.	Not Applicable	
Were any steps taken to minimize the effects of subjective bias when allocating animals/samples to treatment (e.g. **randomization procedure**)? If yes, have they been described?	Not Applicable	
Include a statement about **blinding** even if no blinding was done.	Yes	For determination of mitochondrial morphology in various expeirments described in this manuscript, we blinded the samples to the researcher prior to analysis. This is described in the Materials and Methods section titled "Fluorescence Microscopy"
Describe **inclusion/exclusion criteria** if samples or animals were excluded from the analysis. Were the criteria pre-established?<br><br>If sample or data points were omitted from analysis, report if this was due to attrition or intentional exclusion and provide justification.	Not Applicable	
For every figure, are **statistical tests** justified as appropriate? Do the data meet the assumptions of the tests (e.g., normal distribution)? Describe any methods used to assess it. Is there an estimate of variation within each group of data? Is the variance similar between the groups that are being statistically compared?	Yes	The statistics tests employed are described in the individual figure legends and the Statistical Analysis section of the Materials and Methods.

**Sample definition and in-laboratory replication**	**Information included in the manuscript?**	**In which section is the information available?** (Reagents and Tools Table, Materials and Methods, Figures, Data Availability Section)
In the figure legends: state number of times the experiment was **replicated** in laboratory.	Yes	Individual figure legends.
In the figure legends: define whether data describe **technical or biological replicates**.	Yes	Individual figure legends.

Ethics

**Ethics**	**Information included in the manuscript?**	**In which section is the information available?** (Reagents and Tools Table, Materials and Methods, Figures, Data Availability Section)
Studies involving **human participants**: State details of **authority granting ethics approval** (IRB or equivalent committee(s), provide reference number for approval.	Not Applicable	
Studies involving **human participants**: Include a statement confirming that **informed consent** was obtained from all subjects and that the experiments conformed to the principles set out in the WMA Declaration of Helsinki and the Department of Health and Human Services Belmont Report.	Not Applicable	
Studies involving **human participants:** For publication of **patient photos**, include a statement confirming that consent to publish was obtained.	Not Applicable	
Studies involving experimental **animals**: State details of **authority granting ethics approval** (IRB or equivalent committee(s), provide reference number for approval. Include a statement of compliance with ethical regulations.	Not Applicable	
Studies involving **specimen and field samples:** State if relevant **permits** obtained, provide details of authority approving study; if none were required, explain why.	Not Applicable	

**Dual Use Research of Concern (DURC)**	**Information included in the manuscript?**	**In which section is the information available?** (Reagents and Tools Table, Materials and Methods, Figures, Data Availability Section)
Could your study fall under dual use research restrictions? Please check biosecurity documents and list of **select agents and toxins** (CDC): https://www.selectagents.gov/sat/list.htm	Not Applicable	
If you used a select agent, is the security level of the lab appropriate and reported in the manuscript?	Not Applicable	
If a study is subject to dual use research of concern regulations, is the name of the **authority granting approval and reference number** for the regulatory approval provided in the manuscript?	Not Applicable	

Reporting

The MDAR framework recommends adoption of discipline-specific guidelines, established and endorsed through community initiatives. Journals have their own policy about requiring specific guidelines and recommendations to complement MDAR.

**Adherence to community standards**	**Information included in the manuscript?**	**In which section is the information available?** (Reagents and Tools Table, Materials and Methods, Figures, Data Availability Section)
State if relevant guidelines or checklists (e.g., **ICMJE, MIBBI, ARRIVE, PRISMA**) have been followed or provided.	Not Applicable	
For **tumor marker prognostic studies,** we recommend that you follow the **REMARK** reporting guidelines (see link list at top right). See author guidelines, under 'Reporting Guidelines'. Please confirm you have followed these guidelines.	Not Applicable	
For **phase II and III randomized controlled trials**, please refer to the **CONSORT** flow diagram (see link list at top right) and submit the CONSORT checklist (see link list at top right) with your submission. See author guidelines, under 'Reporting Guidelines'. Please confirm you have submitted this list.	Not Applicable	

Data Availability

**Data availability**	**Information included in the manuscript?**	**In which section is the information available?** (Reagents and Tools Table, Materials and Methods, Figures, Data Availability Section)
Have **primary datasets** been deposited according to the journal's guidelines (see 'Data Deposition' section) and the respective accession numbers provided in the Data Availability Section?	Not Applicable	
Were **human clinical and genomic datasets** deposited in a public access-controlled repository in accordance to ethical obligations to the patients and to the applicable consent agreement?	Not Applicable	
Are **computational models** that are central and integral to a study available without restrictions in a machine-readable form? Were the relevant accession numbers or links provided?	Not Applicable	
If publicly available data were reused, provide the respective **data citations in the reference list**.	Not Applicable	
